# Enantioselective synthesis of (–)-chloramphenicol *via* silver-catalysed asymmetric isocyanoacetate aldol reaction†
†Electronic supplementary information (ESI) available: Experimental procedures, characterisation data, copies of ^1^H and ^13^C NMR spectra, and HPLC traces. See DOI: 10.1039/c5ob02141c



**DOI:** 10.1039/c5ob02141c

**Published:** 2015-10-29

**Authors:** Allegra Franchino, Pavol Jakubec, Darren J. Dixon

**Affiliations:** a Chemistry Research Laboratory , Department of Chemistry , University of Oxford , 12 Mansfield Road , Oxford , OX1 3TA , UK . Email: darren.dixon@chem.ox.ac.uk

## Abstract

The highly enantio- and diastereoselective aldol reaction of isocyanoacetates catalysed by Ag_2_O and cinchona-derived amino phosphines applied to the synthesis of (–)- and (+)-chloramphenicol is described. The concise synthesis showcases the utility of this catalytic asymmetric methodology for the preparation of bioactive compounds possessing α-amino-β-hydroxy motifs.

## Abstract 2

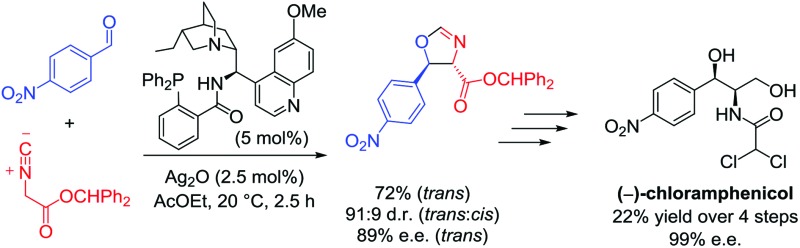
A concise synthesis of (–)-chloramphenicol, based on the catalytic asymmetric aldol reaction between 4-nitrobenzaldehyde and benzhydryl isocyanoacetate, is reported.

Chiral vicinal amino alcohols represent a very important class of compounds, which are of interest to synthetic chemists not only as valuable building blocks and chiral auxiliaries, but also by virtue of their pharmacological properties.^1^ Bioactive vicinal amino alcohols of differing complexity include the broad spectrum antibiotics chloramphenicol^2^ (**1**) and thiamphenicol^3^ (**2**), the protease inhibitor for HIV treatment saquinavir^4^ (**3**) and the antihypertensive drug aliskiren^5^ (**4**, Fig. 1). Among others,^1,6^ a privileged access to these structures is offered by the aldol reaction of glycine equivalents,^7^ including isocyanoacetates,^8^ followed by reduction of the carboxylic group. Recently, our group developed a cooperative catalytic system consisting of a Lewis acid (Ag^+^) and a cinchona-derived amino phosphine ligand, bearing both Brønsted and Lewis basic sites, for the activation of isocyanoacetate pronucleophiles towards electrophiles, such as aldehydes,^9^ ketimines^10^ and ketones.^11^

**Fig. 1:**
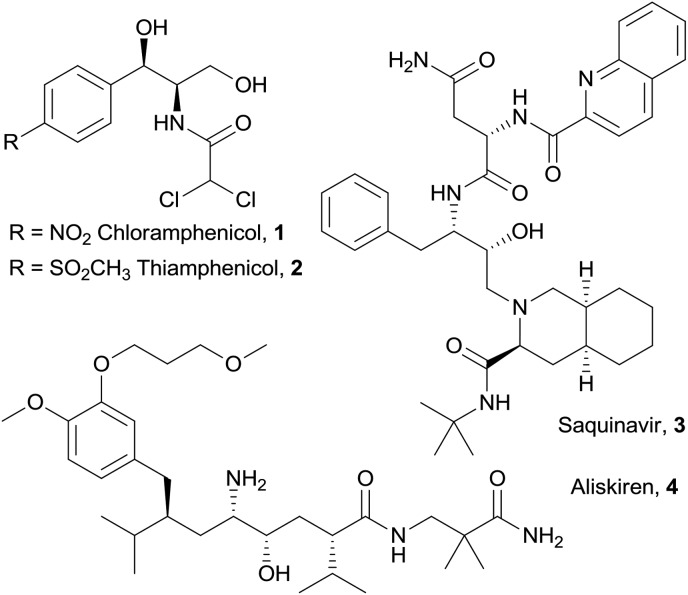
Selected pharmaceuticals containing vicinal amino alcohol fragments.

As a direct demonstration of the utility of this asymmetric methodology, herein we report a short asymmetric synthesis of (–)-chloramphenicol,^12^ which is the first one relying on a catalytic enantio- and diastereoselective aldol reaction.^13^ According to our retrosynthetic plan, outlined in Scheme 1, (–)-chloramphenicol would be derived through standard chemical manipulations from the *trans* oxazoline (4*S*,5*R*)-**6**.^14^ It was envisioned that the latter could be obtained *via* the Ag-catalysed asymmetric isocyanoacetate aldol reaction (IAR) between a suitable isocyanoacetate ester^15^ **7** and 4-nitrobenzaldehyde (**8**). Specifically, on the basis of our previous work^9^ it was anticipated that the use of cinchonine-derived amino phosphine **L-1** as chiral ligand in the IAR would provide oxazoline **6** with the desired absolute configuration for the preparation of (–)-chloramphenicol (Table 1).

**Scheme 1:**
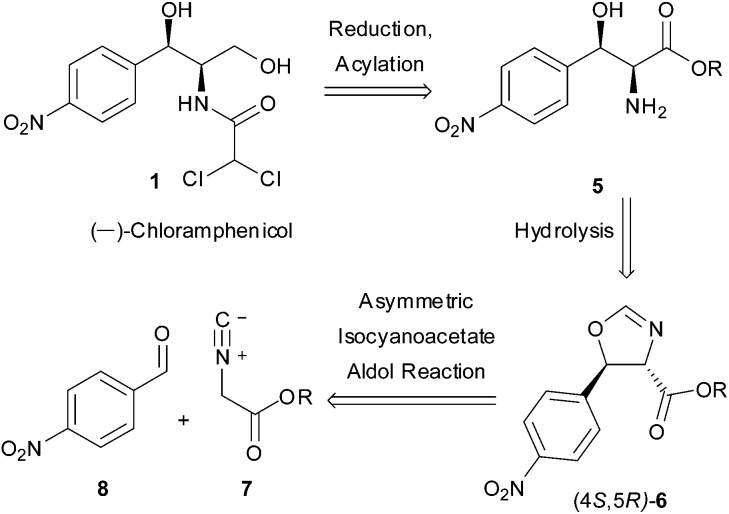
Retrosynthetic approach to (–)-chloramphenicol.

**Table 1:** Temperature and concentration screening in the isocyanoacetate aldol reaction between **8** and **7a** ^*a*^

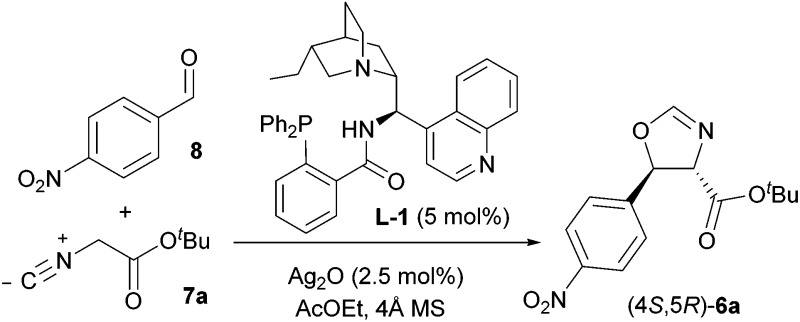						
Entry	*T* (°C)	[**7a**] (M)	Time (h)	Yield^*b*^ (%)	d.r.^*c*^ (*trans* : *cis*)	e.e.^*d*^ (%)
1^*e*^	–20	0.3	21^*f*^	45	91 : 9	44
2^*e*^	20	0.3	0.25	59	91 : 9	53
3	20	0.05	0.5	73	91 : 9	69
4	20	0.01	0.5	70	91 : 9	78
5	20	0.0025	17^*g*^	70	90 : 10	80
6	0	0.01	16^*f*^	72	90 : 10	72
7	50	0.01	2	61	88 : 12	65

^*a*^Reaction performed on 0.25 mmol of **7a** using 1.1 eq. of **8**. Configuration of **6a** assigned by analogy with previous work.^9^

^*b*^Isolated yield of *trans* diastereomer after FCC.

^*c*^d.r. determined by ^1^H NMR analysis of the crude reaction mixture.

^*d*^e.e. of *trans* diastereomer determined by HPLC on chiral stationary phase.

^*e*^0.50 mmol of **7a**.

^*f*^Stirred overnight, as TLC control after 3 hours indicated that the reaction was progressing.

^*g*^Stirred overnight, as TLC control after 6 hours indicated that the reaction was progressing.

Our investigation thus began by performing the reaction between **8** and *tert*-butyl isocyanoacetate **7a** under the conditions that had already been optimised for a range of aldehydes, namely in AcOEt at –20 °C and 0.3 M concentration, employing 5 mol% **L-1** and 2.5 mol% Ag_2_O. To our surprise, the desired *trans* oxazoline **6a** was obtained with modest yield (45%) and low enantioselectivity (44% e.e., Table 1, entry 1), pointing out the need for optimisation of the IAR. Screening of several reaction parameters (concentration, temperature, solvent, nature of the isocyanoacetate ester group, structure of the ligand and Ag/ligand ratio) was therefore undertaken, and the main findings are reported below.

Adjustment of the temperature to 20 °C was beneficial both for yield (59%) and enantiocontrol (53% e.e., entry 2). At this temperature, dilution of the reaction mixture to 0.01 M isocyanoacetate concentration afforded the desired product **6a** in 30 minutes with good yield and stereoselectivity (70% yield, 91 : 9 d.r., 78% e.e., entry 4). These conditions improved solubility, diminishing a competitive non-asymmetric background reaction catalysed by Ag_2_O only, which we hypothesized was responsible for the poor enantioselectivity observed at lower temperature and higher concentration. At the same time, dilution of the reaction mixture increased the yield of the product by reducing the amount of undesired double aldol side product. Further dilution to 0.0025 M resulted in slightly improved enantiocontrol (80% e.e., entry 5) over longer reaction time, but it was discarded for practical scale-up reasons. A temperature screen at 0.01 M (entries 6 and 7) revealed that 20 °C was the optimal temperature. A quick solvent survey confirmed AcOEt to be optimal (see ESI, Table S1†).

With the optimised reaction conditions established, the performance of isocyanoacetates with different ester groups was then investigated (Table 2). Methyl isocyanoacetate **7b** (entry 2) performed better than its *tert*-butyl analogue **7a** (entry 1), suggesting that excessive steric bulk hampered the transmission of stereochemical information. However, the presence of a benzyl or benzhydryl group was well-tolerated: from isocyanoacetates **7c** and **7f** the desired oxazolines could be obtained with the highest enantioselectivity (87% e.e., entries 3 and 7). Isocyanoacetates **7d** and **7e**, possessing 4-methoxybenzyl and 3,5-bis(trifluoromethyl)benzyl groups respectively, provided enantioenriched oxazolines with similar e.e. (86% and 84% e.e. respectively, entries 5 and 6), suggesting that electronic factors didn't play a major role in enantiocontrol.

**Table 2:** Pronucleophile and ligand screening in the isocyanoacetate aldol reaction between **8** and **7** ^*a*^

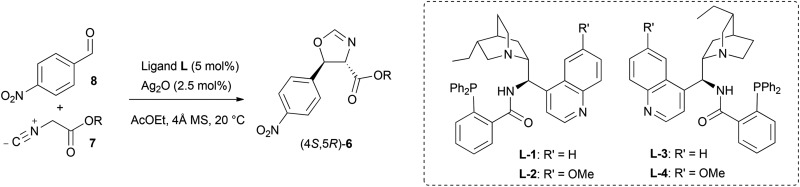							
Entry	R	**7**	Ligand	Time (min)	Yield^*b*^ (%)	d.r.^*c*^ (*trans* : *cis*)	e.e.^*d*^ (%)
1	(CH_3_)_3_C	**7a**	**L-1**	30	70	91 : 9	78
2	CH_3_	**7b**	**L-1**	100	80	88 : 12	82
3	PhCH_2_	**7c**	**L-1**	180	61	90 : 10	87
4	PhCH_2_	**7c**	**L-2**	80	64	90 : 10	87
5	4-(OCH_3_) C_6_H_4_CH_2_	**7d**	**L-2**	60	63	89 : 11	86
6	3,5-(CF_3_)_2_ C_6_H_3_CH_2_	**7e**	**L-2**	60	56	90 : 10	84
7	Ph_2_CH	**7f**	**L-1**	100	81	93 : 7	87
8	Ph_2_CH	**7f**	**L-2**	45	78	91 : 9	89
9	Ph_2_CH	**7f**	**L-3**	200	82	93 : 7	88^*e*^
10	Ph_2_CH	**7f**	**L-4**	60	68	92 : 8	93^*e*^

^*a*^Reaction performed on 0.25 mmol of **7** (0.01 M in AcOEt) using 1.1 eq. of **8**. Configuration of **6** assigned by analogy with previous work.^9^

^*b*^Isolated yield of *trans* diastereomer after FCC.

^*c*^d.r. determined by ^1^H NMR analysis of the crude reaction mixture.

^*d*^e.e. of *trans* diastereomer determined by HPLC on chiral stationary phase.

^*e*^Opposite enantiomer obtained.

Next the effect of fine tuning of the ligand was taken into account by testing four amino phosphines prepared from 9-amino(9-deoxy) epicinchona alkaloids (Table 2). The use of quinidine-derived **L-2** in the reaction between the benzhydryl isocyanoacetate **7f** and **8** resulted in further improved enantiocontrol (89% e.e., entry 8), whereas no boost in enantioselectivity was observed starting from benzyl isocyanoacetate **7c** (87% e.e., entry 4). The pseudoenantiomeric catalytic system comprising quinine-derived **L-4** afforded the enantiomeric oxazoline (4*R*,5*S*)-**6f** in slightly lower yield (68%) and better stereocontrol (92 : 8 d.r., 93% e.e., entry 10).

Finally catalyst loading studies confirmed the ideal Ag/ligand ratio to be 1 : 1, specifically with 2.5 mol% Ag_2_O and 5 mol% **L-2** (see ESI, Table S2†).

After having successfully improved yield and stereocontrol for the isocyanoacetate aldol reaction, oxazoline (4*S*,5*R*)-**6f** was prepared on 2.5 mmol scale with 72% yield and 89% e.e.,^16^ and then was readily elaborated to the target molecule (Scheme 2). Ring opening of **6f** using thionyl chloride in methanol proceeded with 75% yield to afford amino alcohol **5**, whose enantiomeric purity could be improved to 98% e.e. by a single recrystallisation from toluene (61% yield, first crop). The amino alcohol was then acylated with dichloroacetyl chloride to provide dichloroacetamide **9** in 83% yield. Finally, chemoselective reduction of the ester group with excess sodium borohydride delivered (–)-chloramphenicol in 80% yield and 99% e.e.^17^ (+)-Chloramphenicol was prepared in an analogous manner from oxazoline (4*R*,5*S*)-**6f**.^17^

**Scheme 2:**
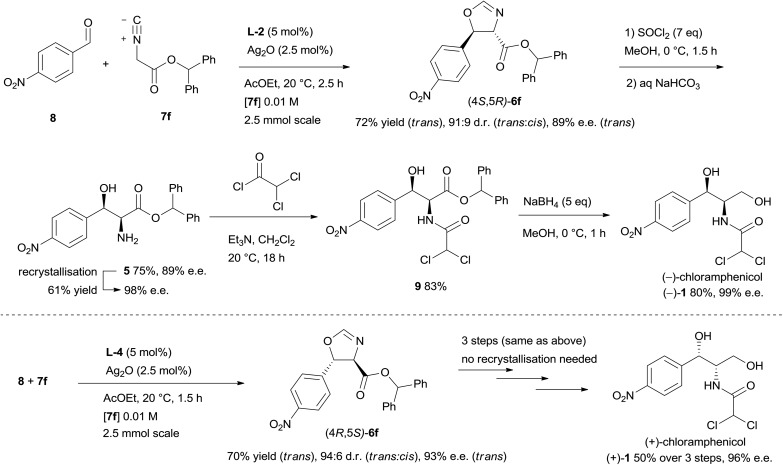
Synthesis of (–)- and (+)-chloramphenicol.

In summary, a catalytic asymmetric synthesis of (–)-chloramphenicol has been accomplished, delivering the target molecule in 4 steps and 22% yield calculated from 4-nitrobenzaldehyde. The concise synthetic route relies on the enantio- and diastereoselective aldol reaction of isocyanoacetates catalysed by Ag_2_O and cinchona-derived amino phosphine ligands. Extensive screening of the reaction parameters has been undertaken to optimise the key step, eventually achieving the formation of the two contiguous stereocentres of the target molecule with good enantiocontrol. The present work demonstrates the utility of this asymmetric methodology for the preparation of bioactive molecules bearing an α-amino-β-hydroxy motif.

The authors gratefully acknowledge the EPSRC (leadership fellowship to D.J.D. and postdoctoral fellowship to P.J.) and the People Programme (Marie Curie Actions) of the European Union's Seventh Framework Programme (A.F., FP7/2007-2013, REA grant agreement no. 316955).
