# Supplementary material for: Enantioselective synthesis of (–)-chloramphenicol via silver-catalysed asymmetric isocyanoacetate aldol reaction
Source: Org Biomol Chem. 2015 Oct 29;14(1):93–6. doi: 10.1039/c5ob02141c (PMC4756631; doi:10.1039/c5ob02141c)
Supplement: Supplementary file 1 [file OB-014-C5OB02141C-s001.pdf]

Electronic Supplementary Material (ESI) for Organic & Biomolecular Chemistry.
This journal is © The Royal Society of Chemistry 2015

Supporting Information

Enantioselective Synthesis of (–)-Chloramphenicol via Silver-Catalysed Asymmetric Isocyanoacetate Aldol Reaction

Allegra Franchino, Pavol Jakubec and Darren J. Dixon

Chemistry Research Laboratory, Department of Chemistry, University of Oxford,
12 Mansfield Road, Oxford, OX1 3TA, United Kingdom

Table of Contents

1. Supplementary tables **S3**

2. Practical experimental **S4**

2.1. General remarks S4

2.2. Synthesis of 2-formamidoacetates **10** S5

2.3. Synthesis of 2-isocyanoacetates **7** S8

2.4. Synthesis and characterisation of oxazolines **6** S10

2.5. Synthesis and characterisation of **5** S14

2.6. Synthesis and characterisation of **9** S15

2.7. Synthesis and characterisation of (–)-chloramphenicol and (+)-chloramphenicol (**1**) S16

3. ^{1}H and ^{13}C NMR spectra **S17**

4. HPLC traces **S30**

5. References **S43**

1. Supplementary tables

Table S1. Solvent screening in the isocyanoacetate aldol reaction between 8 and 7a[a]

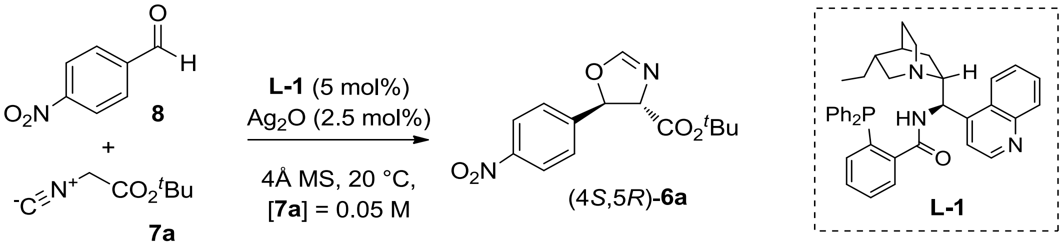

Entry	Solvent	Time (min)	Yield[b] (%)	Dr[c] (*trans*:*cis*)	Ee[d] (%)
1	AcOEt	30	73	91:9	69
2	toluene	40	69	91:9	60
3	CH_3CN	30	65	82:18	61
4	MTBE	60	78	90:10	52
5[e]	AcOEt	30	44	92:8	45
6[e]	CH_2Cl_2	30	47	82:18	37

[a] 0.25 mmol, **7a**; 1.2 equiv, **8**. [b] Isolated yield of *trans* diastereomer after FCC. [c] Dr determined by ^{1}H NMR analysis of the crude reaction mixture. [d] Ee of *trans* diastereomer determined by HPLC on chiral stationary phase. [e] Reaction performed with [**7a**] = 0.3 M, starting at −78 °C and then allowing the reaction mixture to warm up to 20 °C over 30 minutes.

Table S2. Catalyst loading studies in the isocyanoacetate aldol reaction between 8 and 7f

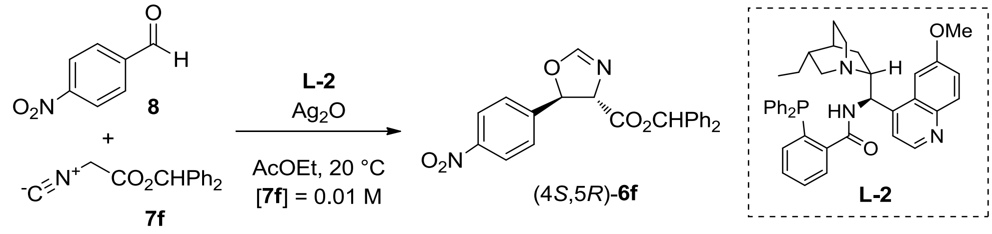

Entry	**7f** (mmol)	**L-2** (mol %)	Ag_2O (mol %)	Time (h)	Yield[a] (%)	Dr[b] (*trans*:*cis*)	Ee[c] (%)
1	2.5	5	2.5	2.5	72	91:9	90
2	2.5	0	2.5	24	51	91:9	0
3	0.5	10	5	0.75	67	91:9	89
4	5.0	5	1.25	2.5	70	91:9	88

[a] Isolated yield of *trans* diastereomer after FCC. [b] Dr determined by ^{1}H NMR analysis of the crude reaction mixture. [c] Ee of *trans* diastereomer determined by HPLC on chiral stationary phase.

2. Practical experimental

2.1. General Remarks

Reactions were performed with magnetic stirring under a positive pressure of nitrogen, unless otherwise stated. Chemicals were obtained from commercial suppliers and used as received.

Bulk solutions were evaporated under reduced pressure using a Büchi rotary evaporator. All solvents were commercially supplied or dried by filtration through activated alumina (powder ~150 mesh, pore size 58 Å, basic, Sigma-Aldrich) columns. Petrol ether (PE) refers to distilled light petroleum of fraction (30 - 40 °C).

Flash column chromatography (FCC) was carried out using Merck silica gel 60 (40-63 μm) as stationary phase.[1] All reactions were followed by thin-layer chromatography (TLC) when practical, using Merck Kieselgel 60 F_{254} fluorescent treated silica. Visualisation was accomplished under UV light (λ_{max}= 254 nm) and by staining with aqueous potassium permanganate alkaline solution or vanillin staining dip (prepared by adding 2.5 mL of concentrated H_2SO_4 to a solution of 15 g of vanillin in 250 mL EtOH 95%). Enantiomeric excesses were determined by HPLC analysis on an Agilent 1200 Series instrument using the chiral stationary phase column specified in the individual experiment and by comparing the sample with the appropriate racemic mixture.

NMR spectra were recorded on Bruker spectrometers operating at 400 or 500 MHz (^{1}H resonance). Proton chemical shifts (*δ*) are given in parts per million (ppm) relative to tetramethylsilane (TMS) with the solvent resonance as internal standard:[2] $CDCl_3$, δ = 7.26 ppm; CD_2Cl_2, δ = 5.32 ppm; $(CD_3)_2SO$, δ = 2.50 ppm; $(CD_3)_2CO$, δ = 2.05 ppm. ^{1}H NMR spectra are reported as follows: ppm (number of protons, multiplicity, coupling constants). The following abbreviations are used to describe spin multiplicity: s = singlet, d = doublet, t = triplet, q = quartet, dd = doublet of doublets, dt = doublet of triplets, m = multiplet, br = broad signal, app = apparent. Coupling constants (*J*) are given in Hertz (Hz) and are rounded to the nearest integer or half integer. ^{13}C-NMR spectra were recorded with complete proton decoupling. Carbon chemical shifts are reported in ppm (δ) relative to TMS with the solvent resonance as internal standard:[2] $CDCl_3$, δ = 77.16 ppm; CD_2Cl_2, δ = 53.84 ppm; $(CD_3)_2SO$, δ = 39.52 ppm; $(CD_3)_2CO$, δ = 29.84 ppm. Two-dimensional NMR spectroscopy experiments (COSY, HSQC and HMBC) were used where appropriate to assist in the assignment of signals in ^{1}H and ^{13}C spectra and data are not reported.

High resolution mass spectra (HRMS) were recorded by the University of Oxford mass spectrometry staff on a Bruker MicroTOF mass spectrometer equipped with an ESI source or on a Micromass GCT equipped with an EI source.

Infrared absorption spectra (IR) were recorded on a Bruker Tensor 27 FT-IR spectrometer from a compressed sample of the solid or from a thin film (the sample was dissolved in $CHCl_3$ or acetone and the solvent evaporated) on a diamond ATR module. Bands (ν_{max}) are reported in wavenumbers (cm^{-1}) and their intensity is indicated as strong (s), medium (m) or weak (w).

Optical rotations were recorded using a Perkin Elmer 341 polarimeter. $[\alpha]_D^T$ values, reported in 10^{-1} deg cm^2 g^{-1}, are calculated on the average value of at least six consecutive readings. Concentrations (c) are quoted in g/100 mL with the appropriate number of significant figures; D refers to the D-line of sodium (589 nm); temperatures (T) are given in degrees Celsius (°C).

Melting points (m.p.) were recorded using a Reichert hot-stage microscope apparatus equipped with an analogic thermometer (2 °C graduation marks) or a Leica Galen III hot-stage microscope apparatus with digital thermometer and are reported uncorrected.

Compound names are those generated by Reaxys following the IUPAC nomenclature.

N-formyl glycine was synthesized following literature procedures.[3] Amino phosphine **L-1** and **L-3** were prepared from cinchonine and cinchonidine respectively following literature procedures.[4] Ligands **L-2** and **L-4** were prepared in an analogous fashion from quinidine and quinine respectively.[5] Methyl isocyanoacetate (**7b**) is commercially available.

Scheme S1. Overview of the synthesis of isocyanoacetates 7

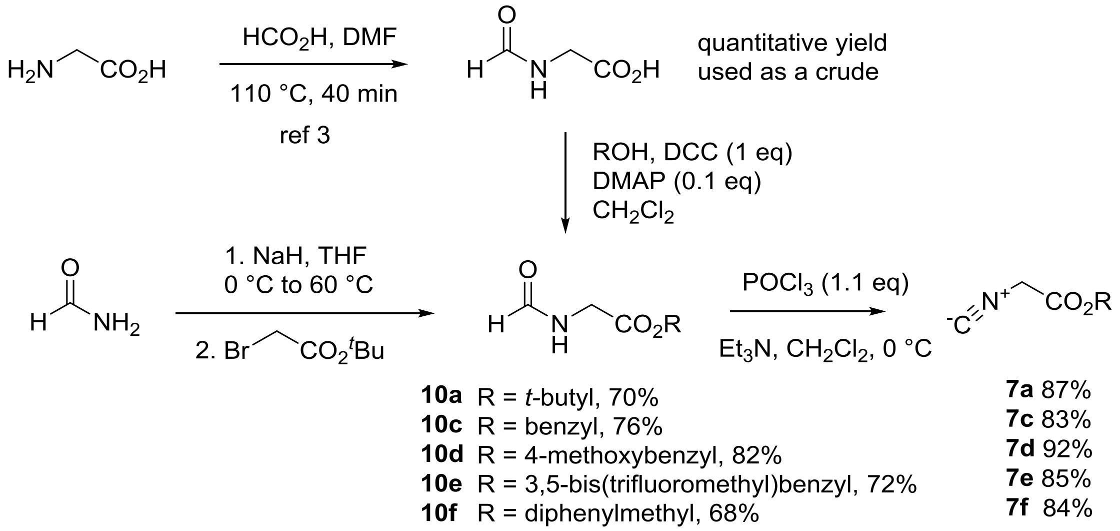

2.2. Synthesis of 2-formamidoacetates 10

tert-Butyl 2-formamidoacetate[6] (10a)

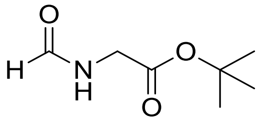

Formamide (45.0 mL, 1.1 mol, 7.4 eq) was added dropwise over 45 minutes to a stirred suspension of NaH (60% dispersion in mineral oil, 6.11 g, 153 mmol, 1.0 eq) in THF (300 mL) at 0 °C. After the addition, the reaction mixture was stirred for 15 minutes at rt, then solvents were removed under reduced pressure. The resulting yellow oil was warmed to 60 °C and *tert*-butyl bromoacetate (23.0 mL, 153 mmol, 1.0 eq) was added. The reaction mixture was stirred for 16 hours at rt, then saturated Na_2CO_3 aqueous solution (100 mL) was added and the aqueous layer was extracted with CH_2Cl_2 (3 × 150 mL). The combined organic phases were washed with water, brine, dried over Na_2SO_4, filtered and evaporated. The crude mixture was purified by FCC (from PE/AcOEt 4:1 to AcOEt), obtaining the title compound **10a** as a pale yellow oil (17.1 g, 70%).

Analytical data are in accordance with those previously reported for this compound.[6]

^{1}H NMR (CD_2Cl_2, 400 MHz): δ 8.18 (1H, s, C<u>H</u>=O, major rotamer), 7.98 (1H, d, *J* = 12.0 Hz, C<u>H</u>=O, minor rotamer), 6.59 (1H, br s, N<u>H</u>), 3.91 (2H, d, *J* = 5.5 Hz, C<u>H</u>$_2$, major rotamer), 3.85 (2H, d, *J* = 6.5 Hz, C<u>H</u>$_2$, minor rotamer), 1.45 (9H, s, C<u>H</u>$_3$).

^{13}C NMR (CD_2Cl_2, 100 MHz): δ 169.1 (<u>C</u>=O), 161.4 (<u>C</u>H=O), 82.5 (<u>C</u>(CH$_3$)), 44.2 (<u>C</u>H$_2$, minor rotamer) 40.9 (<u>C</u>H$_2$, major rotamer), 28.1 (<u>C</u>H$_3$).

Benzyl 2-formamidoacetate (10c)

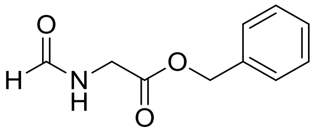

DCC (3.64 g, 17.6 mmol, 1.0 eq) and DMAP (215 mg, 1.76 mmol, 0.1 eq) were added in sequence to a stirred suspension of *N*-formyl glycine (2.00 g, 19.4 mmol, 1.1 eq) and benzyl alcohol (1.8 mL, 18 mmol, 1.0 eq) in CH_2Cl_2 (60 mL) at 0 °C. The reaction mixture was stirred for 16 hours at rt, then the solids were filtered off and solvents were evaporated. Purification of the crude mixture by FCC (PE/AcOEt 1:2) afforded the title compound **10c** as a white solid (2.60 g, 76%).
Analytical data are in accordance with those previously reported for this compound.[7]
^{1}H NMR ($CDCl_3$, 400 MHz): δ 8.26 (1H, s, C$\underline{H}$=O), 7.40-7.34 (5H, m, Ph), 6.08 (1H, br s, N$\underline{H}$), 5.21 (2H, s, C$\underline{H}_2$Ph), 4.14 (2H, d, J = 5.5 Hz, NC$\underline{H}_2$).
^{13}C NMR ($CDCl_3$, 100 MHz): δ 169.5 ($\underline{C}$=O), 161.0 ($\underline{C}$H=O), 135.1 ($\underline{C}_{Ar}$), 128.9 ($\underline{C}_{Ar}$H), 128.8 ($\underline{C}_{Ar}$H), 128.6 ($\underline{C}_{Ar}$H), 67.6 ($\underline{C}H_2$Ph), 40.2 (N$\underline{C}H_2$).
M.p.: 37-39 °C.

(4-Methoxyphenyl)methyl 2-formamidoacetate (10d)

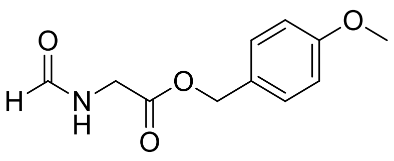

DCC (2.48 g, 12.0 mmol, 1.0 eq) and DMAP (150 mg, 1.20 mmol, 0.1 eq) were added in sequence to a stirred suspension of *N*-formyl glycine (1.36 g, 13.2 mmol, 1.1 eq) and 4-methoxybenzyl alcohol (1.5 mL, 12 mmol, 1.0 eq) in CH_2Cl_2 (60 mL) at 0 °C. The reaction mixture was stirred for 24 hours at rt, then filtered through Celite®. The filtrate was washed with water (15 mL) and brine (15 mL), dried over Na_2SO_4, filtered and concentrated. Purification of the crude mixture by FCC (PE/AcOEt 1:2) afforded the title compound **10d** as a white solid (2.20 g, 82%).
^{1}H NMR ($CDCl_3$, 400 MHz): δ 8.21 (1H, s, C$\underline{H}$=O, major rotamer), 8.01 (1H, d, J = 12.0 Hz, C$\underline{H}$=O, minor rotamer), 7.28 (2H, d, J = 8.5 Hz, $C_{Ar}\underline{H}$), 6.88 (2H, d, J = 8.5 Hz, $C_{Ar}\underline{H}$), 6.38 (1H, br s, N$\underline{H}$, major rotamer),), 6.21 (1H, br s, N$\underline{H}$, minor rotamer), 5.12 (2H, s, OC$\underline{H}_2$), 4.08 (2H, d, J = 5.5 Hz, NC$\underline{H}_2$, major rotamer), 3.98 (2H, d, J = 6.0 Hz, NC$\underline{H}_2$, minor rotamer), 3.80 (3H, s, OC$\underline{H}_3$).
^{13}C NMR ($CDCl_3$, 100 MHz): δ 169.5 ($\underline{C}$=O), 164.7 ($\underline{C}$H=O, minor rotamer), 161.3 ($\underline{C}$H=O, major rotamer), 160.0 ($\underline{C}_{Ar}$), 130.5 ($\underline{C}_{Ar}$H), 127.1 ($\underline{C}_{Ar}$), 114.1 ($\underline{C}_{Ar}$H), 67.4 (O$\underline{C}H_2$), 55.4 (O$\underline{C}H_3$), 43.3 (N$\underline{C}H_2$, minor rotamer), 40.1 (N$\underline{C}H_2$, major rotamer).
HRMS (ESI, MeOH): calcd. for $[M+Na]^+$ ($C_{11}H_{13}NNaO_4$) 246.0737, found 246.0735.
M.p.: 56-57 °C.
IR: 3319 (w, br), 1744 (s, OC=O stretch), 1666 (s, HC=O stretch), 1613 (m), 1515 (s), 1463 (w), 1386 (m), 1355 (w), 1247 (s), 1173 (s), 1031 (m), 822 (m).

[3,5-Bis(trifluoromethyl)phenyl]methyl 2-formamidoacetate (10e)

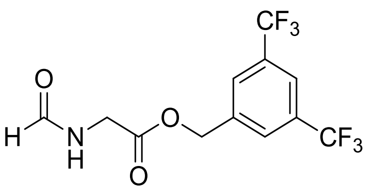

DCC (2.10 g, 10.0 mmol, 1.0 eq) and DMAP (122 mg, 1.00 mmol, 0.1 eq) were added in sequence to a stirred suspension of *N*-formyl glycine (1.13 g, 11.0 mmol, 1.1 eq) and 3,5-bis(trifluoromethyl) benzyl alcohol (2.49 g, 10.0 mmol, 1.0 eq) in CH_2Cl_2 (50 mL) at 0 °C. The reaction mixture was stirred for 16 hours at rt, then filtered

through Celite®. The filtrate was washed with water (15 mL) and brine (15 mL), dried over Na_2SO_4, filtered and evaporated to afford a white solid. Purification of the crude mixture by FCC (PE/AcOEt 2:1) afforded the title compound **10e** as a white solid (2.37 g, 72%).

^{1}H NMR ($CDCl_3$, 400 MHz): δ 8.27 (1H, s, CHO, major rotamer), 8.06 (1H, d, J = 11.5 Hz, CHO, minor rotamer), 7.86 (1H, s, $C_{Ar}H$), 7.82 (2H, s, $C_{Ar}H$), 6.22 (1H, br s, NH), 5.30 (2H, s, OCH_2), 4.17 (2H, d, J = 5.5 Hz, NCH_2, major rotamer), 4.10 (2H, d, J = 5.5 Hz, NCH_2, minor rotamer).

^{13}C NMR ($CDCl_3$, 100 MHz): δ 169.3 (C=O), 164.4 (CH=O, minor rotamer), 161.2 (CH=O, major rotamer), 137.7 (C_{Ar}), 132.3 (C_{Ar}, q, $^2J_{CF}$ = 34.0 Hz), 128.4 ($C_{Ar}H$, app d, $^3J_{CF}$ = 3.0 Hz), 123.3 (CF_3, q, $^1J_{CF}$ = 273.0 Hz), 122.7 ($C_{Ar}H$, app quint, $^3J_{CF}$ = 3.5 Hz), 65.7 (OCH_2), 43.2 (NCH_2, minor rotamer), 40.0 (NCH_2, major rotamer).

^{19}F NMR ($CDCl_3$, 376.5 MHz): δ −63.0.

HRMS (ESI, MeOH): calcd. for $[M+Na]^+$ ($C_{12}H_9F_6NNaO_3$) 352.0379, found 352.0370.

M.p.: 79-81 °C.

IR: 3300 (w, br), 1757 (s, OC=O stretch), 1665 (s, HC=O stretch), 1540 (w), 1391 (w), 1358 (w), 1289 (s), 1232 (m), 1169 (m), 1118 (s), 887 (w), 705 (w), 684 (w).

Diphenylmethyl 2-formamidoacetate (10f)

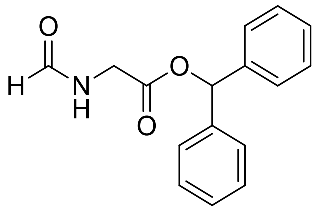

DCC (6.81 g, 33.0 mmol, 1.1 eq) and DMAP (4.03 g, 33.0 mmol, 1.1 eq) were added in sequence to a stirred suspension of *N*-formylglycine (3.74 g, 36.0 mmol, 1.2 eq) and diphenylmethanol (5.53 g, 30.0 mmol, 1.0 eq) in CH_2Cl_2 (150 mL) at rt. The reaction mixture was stirred for 65 hours, then filtered through Celite®. The filtrate was washed with water (50 mL) and brine (50 mL), dried over Na_2SO_4, filtered and concentrated. Purification of the crude mixture by FCC (PE/AcOEt 1:1) afforded the title compound **10f** as a white solid (5.51 g, 68%).
Analytical data are in accordance with those previously reported for this compound.[8]

^{1}H NMR ($CDCl_3$, 400 MHz): δ 8.17 (1H, s, CH=O, major rotamer), 7.99 (1H, s, s, CH=O, minor rotamer), 7.38-7.28 (10H, m, $C_{Ar}H$), 6.93 (1H, s, $CHPh_2$), 6.40 (1H, br s, NH, major rotamer), 6.24 (1H, br s, NH, minor rotamer) 4.17 (2H, d, J = 5.5 Hz, CH_2, major rotamer), 4.02 (2H, d, J = 6.5 Hz, CH_2, minor rotamer).

^{13}C NMR ($CDCl_3$, 100 MHz): δ 168.8 (C=O), 161.5 (CH=O), 139.4 (C_{Ar}), 128.6 ($C_{Ar}H$), 128.2 ($C_{Ar}H$), 127.0 ($C_{Ar}H$), 78.2 ($CHPh_2$), 40.0 (CH_2).

M.p.: 96-98 °C.

2.3. Synthesis of 2-isocyanoacetates 7

tert-Butyl 2-isocyanoacetate[6] (7a)

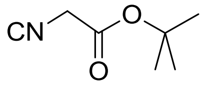

$POCl_3$ (7.48 mL, 80.2 mmol, 1.1 eq) was added dropwise over 20 minutes to a stirred solution of *tert*-butyl 2-formamidoacetate (**10a**, 11.6 g, 72.9 mmol, 1.0 eq) and Et_3N (24.4 mL, 175 mmol, 2.4 eq) in CH_2Cl_2 (150 mL) at 0 °C. Upon addition the colorless reaction mixture became turbid and turned orange, then brown. After stirring at 0 °C for 15 minutes, saturated $NaHCO_3$ aqueous solution (100 mL) was added and the phases were separated. The aqueous layer was extracted twice with CH_2Cl_2 (150 mL), then the collected organic phases were washed with brine, dried over K_2CO_3 and filtered. Evaporation of solvents afforded a brown oil, which was purified by FCC (PE/AcOEt 4:1). The title compound **7a** was obtained as a pale yellow liquid (8.92 g, 87%).

Analytical data match the ones previously reported for this compound.[6]

^{1}H-NMR ($CDCl_3$, 400 MHz): δ 4.08 (2H, s, C<u>H</u>$_2$), 1.43 (9H, s, C<u>H</u>$_3$).

^{13}C-NMR ($CDCl_3$, 100 MHz): δ 162.9 (<u>C</u>=O), 160.3 (N<u>C</u>), 83.9 (<u>C</u>(CH_3)$_3$), 44.2 (<u>C</u>H$_2$), 27.7 (<u>C</u>H$_3$).

Benzyl 2-isocyanoacetate[9] (7c)

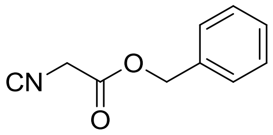

$POCl_3$ (1.33 mL, 14.2 mmol, 1.1 eq) was added dropwise over 5 minutes to a stirred solution of benzyl 2-formamidoaceate (**10c**, 2.50 g, 12.9 mmol, 1.0 eq) and Et_3N (6.1 mL, 44 mmol, 3.4 eq) in CH_2Cl_2 (40 mL) at 0 °C. Upon addition the pale yellow solution turned pink, then orange. After stirring at 0 °C for 1.5 hours, a Na_2CO_3 aqueous solution (4 g in 20 mL of water) was added, the biphasic reaction mixture was stirred at rt for 5 minutes, water (20 mL) was then added and the phases were separated. The aqueous layer was extracted twice with CH_2Cl_2 (60 mL), then the combined organic phases were washed with brine, dried over K_2CO_3 and filtered. Evaporation of solvents afforded a brown oil, which was purified by FCC (PE/AcOEt 3:1). The title compound **7c** was obtained as a yellow solid (1.88 g, 83%).

Analytical data are in accordance with those previously reported for this compound.[9]

^{1}H NMR ($CDCl_3$, 400 MHz): δ 7.39 (5H, s, Ph), 5.23 (2H, s, C<u>H</u>$_2$Ph), 4.22 (2H, s, NC<u>H</u>$_2$).

^{13}C NMR ($CDCl_3$, 100 MHz): δ 163.9 (<u>C</u>=O), 161.1 (N<u>C</u>), 134.4 (<u>C</u>$_{Ar}$), 128.7 (<u>C</u>$_{Ar}$H), 128.6 (<u>C</u>$_{Ar}$H), 128.5 (<u>C</u>$_{Ar}$H), 68.1 (<u>C</u>H$_2$Ph), 43.4 (N<u>C</u>H$_2$).

M.p.: 31-33 °C.

(4-Methoxyphenyl)methyl 2-isocyanoacetate (7d)

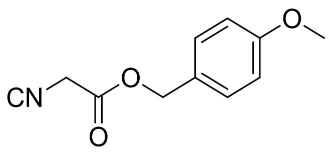

$POCl_3$ (920 µL, 9.86 mmol, 1.1 eq) was added dropwise to a stirred solution of 2-formamidoacetate **10d** (2.00 g, 8.96 mmol, 1.0 eq) and Et_3N (3.0 mL, 22 mmol, 2.4 eq) in CH_2Cl_2 (45 mL) at 0 °C. After stirring at 0 °C for 2.5 hours, the reaction mixture was quenched by addition of saturated $NaHCO_3$ aqueous solution (15 mL). The phases were separated and the aqueous layer was extracted with CH_2Cl_2 (25 mL). The combined organic phases were dried over Na_2SO_4, filtered and concentrated. The crude mixture was purified by FCC (CH_2Cl_2), affording the title compound **7d** as a yellow solid (1.70 g, 92%).

^{1}H NMR ($CDCl_3$, 400 MHz): δ 7.31 (2H, d, J = 8.5 Hz, $C_{Ar}H$), 6.90 (2H, d, J = 8.5 Hz, $C_{Ar}H$) 5.18 (2H, s, OCH_2), 4.22 (2H, s, NCH_2), 3.81 (3H, s, OCH_3).
^{13}C NMR ($CDCl_3$, 100 MHz): δ 164.0 (C=O), 161.4 (NC), 160.2 (C_{Ar}), 130.7 ($C_{Ar}H$), 126.6 (C_{Ar}), 114.2 ($C_{Ar}H$), 68.4 (OCH_2), 55.4 (OCH_3), 43.7 (NCH_2).
HRMS (ESI, MeOH): calcd. for $[M+H]^+$ ($C_{11}H_{12}NO_3$) 206.0812, found 206.0810.
M.p.: 39-41 °C.
IR: 2162 (m, N≡C stretch), 1754 (s, C=O stretch), 1613 (m), 1515 (s), 1462 (w), 1383 (w), 1350 (w), 1245 (s), 1196 (s), 1174 (s), 1010 (m), 821 (m), 705 (w).

[3,5-Bis(trifluoromethyl)phenyl]methyl 2-isocyanoacetate (7e)

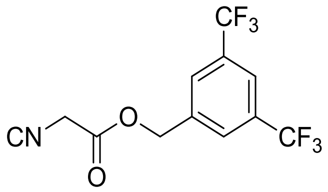

$POCl_3$ (0.62 mL, 6.7 mmol, 1.1 eq) was added dropwise to a stirred solution of 2-formamidoacetate **10e** (2.00 g, 6.07 mmol, 1.0 eq) and Et_3N (2.0 mL, 15 mmol, 2.4 eq) in CH_2Cl_2 (30 mL) at 0 °C. After stirring at 0 °C for 1.5 hours, the reaction mixture was quenched by addition of saturated $NaHCO_3$ aqueous solution (15 mL). The phases were separated and the aqueous layer was extracted with CH_2Cl_2 (30 mL). The combined organic phases were dried over Na_2SO_4, filtered and evaporated to afford an orange oil. The crude mixture was purified by FCC (CH_2Cl_2), obtaining the title compound **7e** as a pale yellow solid (1.60 g, 85%).
^{1}H NMR ($CDCl_3$, 400 MHz): δ 7.89 (1H, s, $C_{Ar}H$), 7.84 (2H, s, $C_{Ar}H$), 5.35 (2H, s, OCH_2), 4.33 (2H, s, NCH_2).
^{13}C NMR ($CDCl_3$, 100 MHz): δ 163.8 (C=O), 162.3 (NC), 136.9 (C_{Ar}), 132.4 (C_{Ar}, q, $^2J_{CF}$ = 34.0 Hz), 128.6 ($C_{Ar}H$, app d, $^3J_{CF}$ = 2.0 Hz), 123.1 (CF_3, q, $^1J_{CF}$ = 273.0 Hz), 123.0 ($C_{Ar}H$, app quint, $^3J_{CF}$ = 3.5 Hz), 66.6 (OCH_2), 43.5 (NCH_2).
^{19}F NMR ($CDCl_3$, 376.5 MHz): δ −63.0.
M.p.: 61-62 °C.
IR: 2165 (w, N≡C stretch), 1766 (m, C=O), 1613 (m), 1394 (w), 1350 (w), 1278 (s), 1173 (s), 1129 (s), 1022 (w), 890 (w), 844 (w), 795 (w), 683 (m).
HRMS (EI): calcd. for $[M]^+$ ($C_{12}H_7F_6NO_2$) 311.0381, found 311.0390.

Diphenylmethyl 2-isocyanoacetate[8] (7f)

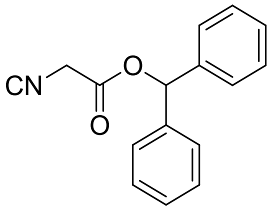

A solution of 2-formamidoacetate **10f** (4.00 g, 14.8 mmol, 1.0 eq) and Et_3N (5.0 mL, 36 mmol, 2.4 eq) in CH_2Cl_2 (75 mL) was cooled to 0 °C, then $POCl_3$ (1.5 mL, 16 mmol, 1.1 eq) was added dropwise. During the addition the reaction mixture turned yellow. The reaction mixture was stirred at 0 °C for 1.5 hours, then saturated Na_2CO_3 aqueous solution was added (30 mL) and the phases were separated. The organic layer was washed with water (30 mL), brine (30 mL), dried over Na_2SO_4, filtered and concentrated under reduced pressure. The crude mixture was purified by FCC (PE/AcOEt 4:1) affording the title compound **7f** as a pale orange solid (3.13 g, 84%).
Analytical data are in accordance with those previously reported for this compound.[8,10]
^{1}H-NMR ($CDCl_3$, 400 MHz): δ 7.39-7.33 (10H, m, $C_{Ar}H$), 6.99 (1H, s, $CHPh_2$), 4.30 (2H, s, CH_2).

^{13}C-NMR ($CDCl_3$, 100 MHz): δ 163.1 (C=O), 161.7 (NC), 138.9 (C_{Ar}), 128.8 (C_{Ar}H), 128.5 (C_{Ar}H), 127.1 (C_{Ar}H), 79.3 ($CHPh_2$), 43.7 (CH_2).
M.p.: 54 °C.

2.4. Synthesis and characterisation of oxazolines 6

General procedure A for the racemic synthesis of oxazolines 6

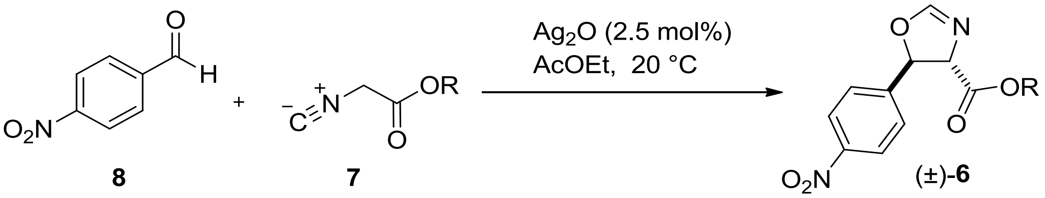

Ag_2O (1.4 mg, 0.00625 mmol, 0.025 eq) was added to a stirred solution of isocyanoacetate **7** (0.250 mmol, 1.0 eq) and 4-nitrobenzaldehyde (**8**, 42.0 mg, 0.275 mmol, 1.1 eq) in AcOEt (25 mL). The resulting mixture was stirred at rt until the isocyanoacetate was consumed (as judged by TLC, PE/AcOEt 4:1, developed with vanillin staining dip). The reaction mixture was quickly filtered through Celite® eluting with AcOEt. The filtrate was concentrated under reduced pressure and purified by FCC (PE/AcOEt) obtaining (±)-**6**.

General procedure B for the enantioselective synthesis of oxazolines 6

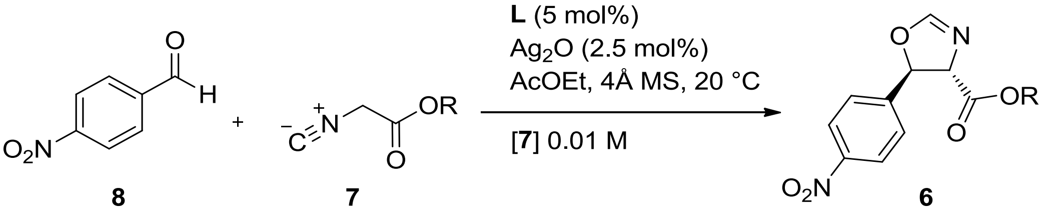

The desired ligand **L** (0.0125 mmol, 0.05 eq), Ag_2O (1.4 mg, 0.00625 mmol, 0.025 eq) and powdered 4Å MS (72 mg per 0.25 mmol of **7**) were stirred in AcOEt (15 mL) at rt for 15 minutes. A solution of isocyanoacetate **7** (0.250 mmol, 1.0 eq) in AcOEt (5 mL) was then added. After approximately 5 minutes, a solution of 4-nitrobenzaldehyde (**8**, 42.0 mg, 0.275 mmol, 1.1 eq) in AcOEt (5 mL) was added, then the reaction mixture was stirred at rt until the isocyanoacetate was consumed (as judged by TLC, PE/AcOEt 4:1, revealed with vanillin staining dip). The reaction mixture was quickly filtered through Celite® eluting with AcOEt. The filtrate was concentrated under reduced pressure and purified by FCC with the eluent indicated for each compound.

tert-Butyl (4*S*,5*R*)-5-(4-nitrophenyl)-4,5-dihydro-1,3-oxazole-4-carboxylate (6a)

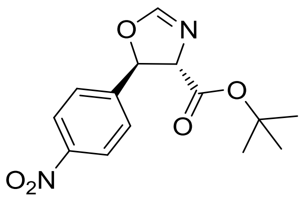

Obtained according to general procedure B, using **L-1** (7.3 mg, 0.0125 mmol, 0.05 eq) and *tert*-butyl isocyanoacetate **7a** (35.3 mg, 0.250 mmol, 1.0 eq). The crude mixture was purified by FCC (from PE/ AcOEt 4:1 to 1:1) obtaining the title compound **6a** as an off-white solid (51.1 mg, 70%). Enantiomeric excess (78% ee) was determined by chiral HPLC analysis under the following conditions: 0.5 mg in 1 mL *i*-PrOH, Chiralpak OD column, hexane/*i*-PrOH 90:10, flow 1.0 mL/min, λ 254 nm, t (major) = 13.6 min, t (minor) = 16.8 min.

^{1}H NMR (DMSO-d$_6$, 500 MHz): δ 8.29 (2H, d, *J* = 8.5 Hz, C$_{Ar}$H), 7.61 (2H, d, *J* = 8.5 Hz, C$_{Ar}$H), 7.54 (1H, d, *J* = 2.0 Hz, N=CH), 5.75 (1H, d, *J* = 7.5 Hz, OCHAr), 4.49 (1H, dd, *J* = 7.5, 2.0 Hz, NCHCO$_2$), 1.46 (9H, s, CH$_3$).
^{13}C NMR (DMSO-d$_6$, 125 MHz): δ 168.8 (C=O), 156.4 (C=N), 147.5 (C$_{Ar}$), 146.7 (C$_{Ar}$), 127.0 (C$_{Ar}$H), 124.0 (C$_{Ar}$H), 81.9 (C(CH$_3$)$_3$), 80.1 (OCHAr), 75.6 (NCHCO$_2$), 27.6 (CH$_3$).
HRMS (ESI, MeOH): calcd. for $[M+Na]^+$ ($C_{14}H_{16}N_2NaO_5$) 315.0951, found 315.0957.
M.p.: 65-70 °C.
IR: 2980 (w), 2927 (w), 1747 (s, C=O stretch), 1637 (m, C=N stretch), 1603 (w), 1511 (s), 1346 (s), 1315 (w), 1229 (m), 1151 (s), 1109 (m), 1081 (s), 1051 (s), 1014 (w), 968 (m), 841 (s), 787 (m), 751 (w), 721 (m), 697 (m), 667 (m).
$[\alpha]_D^{20}$: +119 (c 1.43, AcOEt, sample with 65% ee).

Methyl (4*S*,5*R*)-5-(4-nitrophenyl)-4,5-dihydro-1,3-oxazole-4-carboxylate (6b)

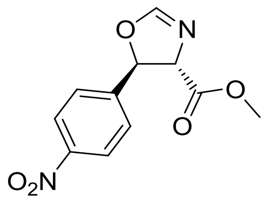

Obtained according to general procedure B, using **L-1** (7.3 mg, 0.0125 mmol, 0.05 eq) and methyl isocyanoacetate (26.1 mg, 0.250 mmol, 1.0 eq). The crude mixture was purified by FCC (PE/AcOEt 3:2 to 1:2) obtaining the title compound **6b** as a pale yellow oil (50.0 mg, 80%).

Enantiomeric excess (82% ee) was determined by chiral HPLC analysis under the following conditions: 0.5 mg in 1 mL *i*-PrOH, Chiralpak AD column, hexane/*i*-PrOH 85:15, flow 1.0 mL/min, λ 240 nm, t (minor) = 17.3 min, t (major) = 26.6 min.
^{1}H NMR is in accordance with that previously reported for this compound.[11]
^{1}H NMR (CDCl$_3$, 400 MHz): δ 8.26 (2H, d, *J* = 8.5 Hz, C$_{Ar}$H), 7.52 (2H, d, *J* = 8.5 Hz, C$_{Ar}$H), 7.13 (1H, d, *J* = 2.0 Hz, N=CH), 5.80 (1H, d, *J* = 8.0 Hz, OCHAr), 4.58 (1H, dd, *J* = 8.0, 2.0 Hz, NCHCO$_2$), 3.87 (3H, s, CH$_3$).
^{13}C NMR (CDCl$_3$, 100 MHz): δ 170.5 (C=O), 156.1 (C=N), 148.2 (C$_{Ar}$), 146.1 (C$_{Ar}$), 126.4 (C$_{Ar}$H), 124.4 (C$_{Ar}$H), 80.9 (OCHAr), 75.7 (NCHCO$_2$), 53.3 (CH$_3$).
HRMS (ESI, MeOH): calcd. for $[M+Na]^+$ ($C_{11}H_{10}N_2NaO_5$) 273.0482, found 273.0481.
IR: 1739 (m, C=O stretch), 1628 (m, C=N stretch), 1606 (w), 1520 (s), 1437 (w), 1346 (s), 1205 (m), 1097 (s), 973 (w), 852 (m), 736 (m), 696 (m).
$[\alpha]_D^{20}$: +198 (c 0.15, AcOEt, sample with 82% ee).

Benzyl (4*S*,5*R*)-5-(4-nitrophenyl)-4,5-dihydro-1,3-oxazole-4-carboxylate (6c)

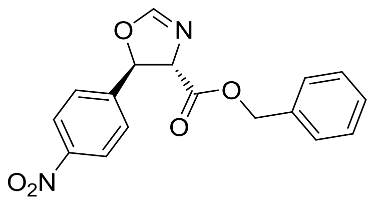

Obtained according to general procedure B, using **L-1** (7.3 mg, 0.0125 mmol, 0.05 eq) and benzyl isocyanoacetate (**7c**, 43.8 mg, 0.250 mmol, 1.0 eq). The crude mixture was purified by FCC (PE/AcOEt 4:1 to 3:1) obtaining the title compound **6c** as a pale yellow solid (49.8 mg, 61%).

Enantiomeric excess (87% ee) was determined by chiral HPLC analysis under the following conditions: 0.5 mg in 1 mL *i*-PrOH/EtOH 1:1, Chiralpak AD column, hexane/*i*-PrOH 85:15, flow 1.0 mL/min, λ 254 nm, t (minor) = 25.3 min, t (major) = 28.4 min.
^{1}H NMR (DMSO-d$_6$, 400 MHz): δ 8.26 (2H, d, *J* = 8.5 Hz, C$_{ArNO2}$H), 7.61 (2H, d, *J* = 8.5 Hz, C$_{ArNO2}$H), 7.57 (1H, d, *J* = 2.0 Hz, N=CH), 7.42-7.35 (5H, m, Ph), 5.88 (1H, d, *J* = 7.5 Hz, OCHAr), 5.23 (2H, s, OCH$_2$Ph), 4.71 (1H, dd, *J* = 7.5, 2.0 Hz, NCHCO$_2$).

^{13}C NMR (DMSO-d$_6$, 100 MHz): δ 169.6 ($\underline{C}$=O), 156.7 ($\underline{C}$=N), 147.5 ($\underline{C}_{ArNO2}$), 146.4 ($\underline{C}_{ArNO2}$), 135.5 ($\underline{C}_{Ph}$), 128.5 ($\underline{C}_{Ar}$H), 128.2 ($\underline{C}_{Ar}$H), 128.1 ($\underline{C}_{Ar}$H), 127.1 ($\underline{C}_{Ar}$H), 124.0 ($\underline{C}_{Ar}$H), 80.0 (O$\underline{C}$HAr), 74.7 (N$\underline{C}$HCO$_2$), 66.7 (O$\underline{C}$H$_2$Ph).
HRMS (ESI, MeOH): calcd. for [M+Na]$^+$ ($C_{17}H_{14}N_2NaO_5$) 349.0795, found 349.0796.
M.p.: 85-87 °C.
IR: 3090 (w), 2962 (w), 2837 (w), 1728 (s, C=O stretch), 1628 (m, C=N stretch), 1606 (w), 1519 (s), 1459 (w), 1380 (w), 1348 (s), 1315 (w), 1242 (s), 1213 (s), 1079 (s), 1039 (w), 1014 (w), 964 (m), 912 (w), 890 (w), 856 (m), 845 (m), 770 (m), 759 (s), 717 (m), 699 (s).
$[\alpha]_D^{20}$: +103 (c 0.75, AcOEt, sample with 87% ee).

(4-Methoxyphenyl)methyl (4*S*,5*R*)-5-(4-nitrophenyl)-4,5-dihydro-1,3-oxazole-4-carboxylate (6d)

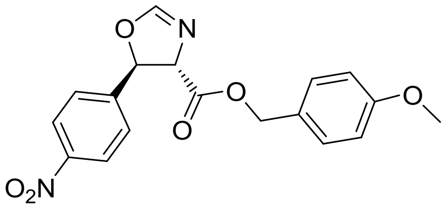

Obtained according to general procedure B, using **L-2** (7.7 mg, 0.0125 mmol, 0.05 eq) and isocyanoacetate **7d** (51.3 mg, 0.250 mmol, 1.0 eq). The crude mixture was purified by FCC (PE/AcOEt 3:1 to 1:1) obtaining the title compound **6d** as a pale yellow oil (56.1 mg, 63%).
Enantiomeric excess (86% ee) was determined by chiral HPLC analysis under the following conditions: 0.5 mg in 1 mL *i*-PrOH/EtOH 1:1, Chiralpak AD column, hexane/*i*-PrOH 80:20, flow 1.0 mL/min, λ 254 nm, t (minor) = 26.2 min, t (major) = 30.5 min.
^{1}H NMR (DMSO-d$_6$, 400 MHz): δ 8.26 (2H, d, *J* = 8.5 Hz, C$_{ArNO2}\underline{H}$), 7.60 (2H, d, *J* = 8.5 Hz, C$_{ArNO2}\underline{H}$), 7.57 (1H, d, *J* = 2.0 Hz, N=C$\underline{H}$), 7.35 (2H, d, *J* = 9.0 Hz, C$_{ArOMe}\underline{H}$), 6.95 (2H, d, *J* = 9.0 Hz, C$_{ArOMe}\underline{H}$), 5.85 (1H, d, *J* = 7.5 Hz, OC$\underline{H}$Ar), 5.15 (2H, s, OC$\underline{H}_2$Ar), 4.66 (1H, dd, *J* = 7.5, 2.0 Hz, NC$\underline{H}$CO$_2$), 3.75 (3H, s, OC$\underline{H}_3$).
^{13}C NMR (DMSO-d$_6$, 100 MHz): δ 169.6 ($\underline{C}$=O), 159.3 ($\underline{C}_{ArOMe}$), 156.7 ($\underline{C}$=N), 147.5 ($\underline{C}_{ArNO2}$), 146.4 ($\underline{C}_{ArNO2}$), 130.1 ($\underline{C}_{ArOMe}$H), 127.4 ($\underline{C}_{ArOMe}$), 127.0 ($\underline{C}_{ArNO2}$H), 124.0 ($\underline{C}_{ArNO2}$H), 113.9 ($\underline{C}_{ArOMe}$H), 80.0 (OC$\underline{H}$Ar), 74.7 (N$\underline{C}$HCO$_2$), 66.6 (O$\underline{C}$H$_2$Ar), 55.1 (O$\underline{C}$H$_3$).
HRMS (ESI, MeOH): calcd. for [M+Na]$^+$ ($C_{18}H_{16}N_2NaO_6$) 379.0890, found 379.0901.
IR: 3359 (w, br), 2935 (w), 2840 (w), 1739 (m, C=O stretch), 1684 (m), 1629 (w, C=N stretch), 1611 (w), 1516 (s), 1462 (w), 1347 (s), 1248 (s), 1175 (m), 1108 (m), 1032 (m), 968 (w), 835 (w), 824 (w), 720 (w).
$[\alpha]_D^{20}$: +142 (c 0.33, AcOEt, sample with 86% ee).

[3,5-Bis(trifluoromethyl)phenyl]methyl (4*S*,5*R*)-5-(4-nitrophenyl)-4,5-dihydro-1,3-oxazole-4-carboxylate (6e)

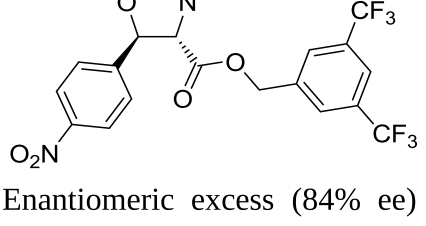

Obtained according to general procedure B, using **L-2** (7.7 mg, 0.0125 mmol, 0.05 eq) and isocyanoacetate **7e** (77.8 mg, 0.250 mmol, 1.0 eq). The crude mixture was purified by FCC (PE/AcOEt 3:1) obtaining the title compound **6e** as a clear oil (64.7 mg, 56%).
Enantiomeric excess (84% ee) was determined by chiral HPLC analysis under the following conditions: 0.5 mg in 1 mL *i*-PrOH/EtOH 1:1, Chiralpak AD column, hexane/*i*-PrOH 80:20, flow 1 mL/min, λ 254 nm, t (minor) = 8.5 min, t (major) = 11.2 min.

^{1}H NMR (DMSO-d$_6$, 500 MHz): δ 8.24 (2H, d, *J* = 9.0 Hz, $C_{ArNO2}\underline{H}$), 8.15 (2H, s, $C_{ArCF3}\underline{H}$), 8.08 (1H, s, $C_{ArCF3}\underline{H}$), 7.63 (2H, d, *J* = 9.0 Hz, $C_{ArNO2}\underline{H}$), 7.60 (1H, d, *J* = 2.0 Hz, N=C$\underline{H}$), 5.94 (1H, d, *J* = 7.5 Hz, OC$\underline{H}$Ar), 5.41 (2H, s, OC$\underline{H}_2$Ar), 4.81 (1H, dd, *J* = 7.5, 2.0 Hz, NC$\underline{H}$CO$_2$).
^{13}C NMR (DMSO-d$_6$, 125 MHz): δ 169.5 ($\underline{C}$=O), 156.9 ($\underline{C}$=N), 147.5 ($\underline{C}_{ArNO2}$), 146.4 ($\underline{C}_{ArNO2}$), 139.2 ($\underline{C}_{ArCF3}$), 130.4 ($\underline{C}_{Ar}CF_3$, q, $^{2}J_{CF}$ = 33.0 Hz), 128.7 ($\underline{C}_{ArCF3}$H, app d, $^{3}J_{CF}$ = 3.0 Hz), 127.2 ($\underline{C}_{ArNO2}$H), 123.9 ($\underline{C}_{ArNO2}$H), 123.3 ($\underline{C}F_3$, q, $^{1}J_{CF}$ = 273.0 Hz), 121.9 ($\underline{C}_{ArCF3}$H, m), 79.9 (O$\underline{C}$HAr), 74.6 (N$\underline{C}$HCO$_2$), 65.2 (O$\underline{C}$H$_2$Ar).
^{19}F NMR (DMSO-d$_6$, 470.6 MHz): δ −61.3.
HRMS (ESI, MeOH): calcd. for $[M+H]^+$ ($C_{19}H_{13}F_6N_2O_5$) 463.0723, found 463.0713.
IR: 1749 (w), 1712 (m, C=O stretch), 1628 (w, C=N stretch), 1526 (m), 1349 (m), 1278 (s), 1222 (m), 1172 (s), 1131 (s), 1108 (s), 890 (w), 843 (m), 705 (m), 602 (m).
$[\alpha]_D^{20}$: +135 (c 0.29, AcOEt, sample with 84% ee).

Diphenylmethyl (4*S*,5*R*)-5-(4-nitrophenyl)-4,5-dihydro-1,3-oxazole-4-carboxylate (6f)

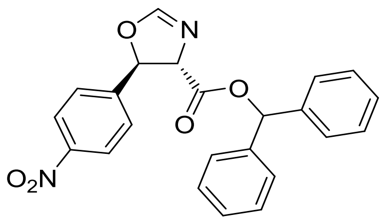

Obtained according to general procedure B, using **L-2** (7.7 mg, 0.0125 mmol, 0.05 eq) and isocyanoacetate **7f** (62.8 mg, 0.250 mmol, 1.0 eq). The crude mixture was purified by FCC (from PE/AcOEt 4:1 to PE/AcOEt 3:1) obtaining the title compound (4*S*,5*R*)**-6f** as an off-white solid (78.4 mg, 78%).

Enantiomeric excess (89% ee) was determined by chiral HPLC analysis under the following conditions: 0.5 mg in 1 mL *i*-PrOH/EtOH 1:1, Chiralpak OD column, hexane/*i*-PrOH 85:15, flow 1.0 mL/min, λ 254 nm, t (major) = 26.1 min, t (minor) = 31.6 min.
^{1}H NMR (DMSO-d$_6$, 400 MHz): δ 8.27 (2H, d, *J* = 8.5 Hz, $C_{ArNO2}\underline{H}$), 7.62-7.60 (3H, m, $C_{ArNO2}\underline{H}$ and N=C$\underline{H}$), 7.45-7.30 (10H, m, Ph), 6.90 (1H, s, OC$\underline{H}$Ph$_2$), 5.87 (1H, d, *J* = 7.5 Hz, OC$\underline{H}$Ar), 4.86 (1H, dd, *J* = 7.5, 2.0 Hz, NC$\underline{H}$CO$_2$).
^{13}C NMR (DMSO-d$_6$, 100 MHz): δ 168.8 ($\underline{C}$=O), 156.9 ($\underline{C}$=N), 147.5 ($\underline{C}_{ArNO2}$), 146.3 ($\underline{C}_{ArNO2}$), 140.0 ($\underline{C}_{Ar}$), 139.9 ($\underline{C}_{Ar}$), 128.6 ($\underline{C}_{Ar}$H), 128.5 ($\underline{C}_{Ar}$H), 128.0 ($\underline{C}_{Ar}$H), 127.9 ($\underline{C}_{Ar}$H), 127.2 ($\underline{C}_{Ar}$H), 126.7 ($\underline{C}_{Ar}$H), 126.4 ($\underline{C}_{ArNO2}$H), 124.0 ($\underline{C}_{ArNO2}$H), 80.0 (O$\underline{C}$HAr), 77.7 (O$\underline{C}$HPh$_2$), 74.8 (N$\underline{C}$HCO$_2$).
HRMS (ESI, MeOH): calcd. for $[M+Na]^+$ ($C_{23}H_{18}N_2NaO_5$) 425.1108, found 425.1114.
M.p.: 39-41 °C.
IR: 1737 (m, C=O stretch), 1627 (m, C=N stretch), 1606 (w), 1522 (s), 1496 (w), 1454 (w), 1347 (s), 1222 (m), 1176 (s), 971 (m), 850 (m), 836 (m), 748 (m), 697 (s), 647 (w).
$[\alpha]_D^{20}$: +152 (c 0.37, AcOEt, sample with 89% ee).

Diphenylmethyl (4*R*,5*S*)-5-(4-nitrophenyl)-4,5-dihydro-1,3-oxazole-4-carboxylate (6f)

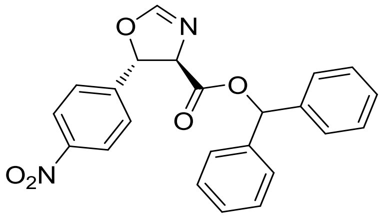

Obtained according to general procedure B using **L-4** (7.7 mg, 0.0125 mmol, 0.05 eq) and isocyanoacetate **7f** (62.8 mg, 0.250 mmol, 1.0 eq), then purified by FCC (from PE/AcOEt 4:1 to PE/AcOEt 3:1) obtaining (4*R*,5*S*)**-6f** as an off-white solid (68.4 mg, 68%).

Enantiomeric excess (93% ee) was determined by chiral HPLC analysis under the following conditions: 0.5 mg in 1 mL *i*-PrOH/EtOH 1:1, Chiralpak OD column, hexane/*i*-PrOH 85:15, flow 1 mL/min, λ 254 nm, t (minor) = 27.6 min, t (major) = 31.5 min.
$[\alpha]_D^{25}$: –156 (c 0.37, AcOEt, sample with 93% ee).

2.5. Synthesis and characterisation of 5

Diphenylmethyl (2*S*,3*R*)-2-amino-3-hydroxy-3-(4-nitrophenyl)propanoate (2*S*,3*R*)-(5)

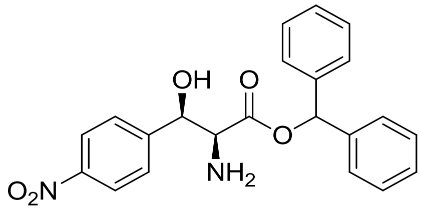

A solution of oxazoline (4*S*,5*R*)-**6f** (683 mg, 1.70 mmol, 1.0 eq) in MeOH (12 mL) was cooled to 0 °C. A solution of thionyl chloride (862 µL, 11.9 mmol, 7.0 eq) in MeOH (12 mL) was cooled to 0 ^{o}C and then added dropwise to the reaction flask. The resulting mixture was stirred at 0 °C for 1.5 hours, then quenched by addition of solid $NaHCO_3$. Volatiles were removed under reduced pressure and the residue was taken up in CH_2Cl_2 and water. The phases were separated and the aqueous layer was extracted with CH_2Cl_2 three times. The combined organic phases were washed with brine, dried over Na_2SO_4, filtered and evaporated to obtain a brown oil. Purification of the crude mixture by FCC (PE/AcOEt 3:2 to 1:2) gave the title compound (2*S*,3*R*)-**5** as a pale yellow solid (499 mg, 75%).

Crystallisation was performed as follow. Compound **5** (150.0 mg, 90% ee) was dissolved in toluene (1 mL) at 100 °C, then slowly allowed to cool down to rt. The resulting solution was left for 16 h at 0 °C, then for 4 days at –20 °C, during which a white solid formed. Toluene was removed with a pipette, then the solid was washed with toluene at rt (3 × 1 mL), each time decanting the solvent off. The solid was then dried (92.0 mg, 61% crystallisation yield, 98% ee).

Enantiomeric excess was determined by chiral HPLC analysis under the following conditions: 0.5 mg in 1 mL *i*-PrOH/EtOH 1:1, Chiralpak AD column, hexane/*i*-PrOH 70:30, flow 0.8 mL/min, λ 220 nm, t (major) = 12.4 min, t (minor) = 15.3 min.

^{1}H NMR (DMSO-d_6, 400 MHz): δ 8.10 (2H, d, *J* = 8.5 Hz, $C_{ArNO2}\underline{H}$), 7.60 (2H, d, *J* = 8.5 Hz, $C_{ArNO2}\underline{H}$), 7.45-7.22 (10H, m, Ph), 6.77 (1H, s, $C\underline{H}Ph_2$), 5.93 (1H, d, *J* = 5.0 Hz, $O\underline{H}$), 5.06 (1H, t, *J* = 4.5 Hz, $ArC\underline{H}OH$), 3.70 (1H, d, *J* = 4.0 Hz, $C\underline{H}NH_2$), 1.79 (2H, br s, $N\underline{H}_2$).

^{13}C NMR (DMSO-d_6, 100 MHz): δ 172.4 ($\underline{C}$=O), 150.7 ($\underline{C}_{ArNO2}$), 146.5 ($\underline{C}_{ArNO2}$), 140.6 ($\underline{C}_{Ar}$), 140.4 ($\underline{C}_{Ar}$), 128.5 ($\underline{C}_{Ar}H$), 128.3 ($\underline{C}_{Ar}H$), 127.8 ($\underline{C}_{Ar}H$), 127.6 ($\underline{C}_{Ar}H$), 126.7 ($\underline{C}_{Ar}H$), 126.4 ($\underline{C}_{ArNO2}H$), 122.8 ($\underline{C}_{ArNO2}H$), 76.7 ($\underline{C}HPh_2$), 73.7 ($Ar\underline{C}HOH$), 60.8 ($\underline{C}HNH_2$).

HRMS (ESI, MeOH): calcd. for $[M+H]^+$ ($C_{22}H_{21}N_2O_5$) 393.1445, found 393.1434.

M.p.: 116-118 °C.

IR: 2923 (w, br), 1716 (m, C=O stretch), 1600 (w), 1513 (m) 1345 (m), 1224 (m), 1197 (m), 998 (w), 920 (w), 771 (m), 742 (m), 701 (s).

$[\alpha]_D^{20}$: +22 (c 0.20, AcOEt, sample with 89% ee).

Diphenylmethyl (2*R*,3*S*)-2-amino-3-hydroxy-3-(4-nitrophenyl)propanoate (2*R*,3*S*)-(5)

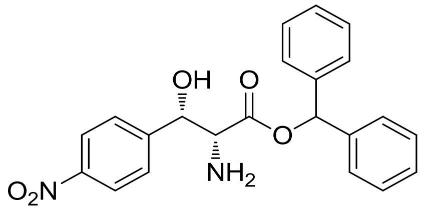

Synthesized and purified in an identical fashion starting from (4*R*,5*S*)-**6f**. The title compound (2*R*,3*S*)-**5** was obtained as an off-white solid (76%) and was not crystallised. Enantiomeric excess (94%) was determined by chiral HPLC analysis under the following conditions: 0.5 mg in 1 mL *i*-PrOH/EtOH 1:1, Chiralpak AD column, hexane/*i*-PrOH 70:30, flow 0.8 mL/min, λ 220 nm, t (minor) = 12.6 min, t (major) = 15.6 min.

$[\alpha]_D^{25}$: –31 (c 0.20, AcOEt, sample with 94% ee).

(±)-**5** was prepared in an identical fashion from (±)-**6f**.

2.6. Synthesis and characterisation of 9

Diphenylmethyl (2*S*,3*R*)-2-(2,2-dichloroacetamido)-3-hydroxy-3-(4-nitrophenyl)propanoate (2*S*,3*R*)-(9)

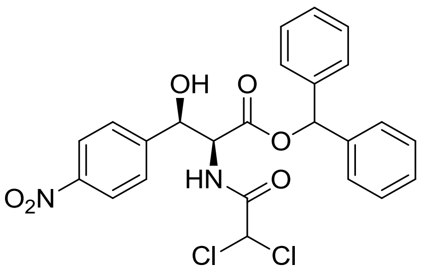

Dichloroacetyl chloride (25 μL, 0.25 mmol, 1.1 eq) was added dropwise to a stirred solution of (2*S*,3*R*)-**5** (90.0 mg, 0.223 mmol, 1.0 eq) and Et_3N (35 μL, 0.25 mmol, 1.1 eq) in CH_2Cl_2 (2.5 mL) at 0 °C. The reaction mixture was stirred at rt for 16 hours, then saturated $NaHCO_3$ aqueous solution (5 mL) was added. The phases were separated and the aqueous layer was extracted with CH_2Cl_2 (3 × 5 mL). The combined organic phases were washed with brine, dried over Na_2SO_4, filtered and evaporated. Purification of the crude mixture by FCC (PE/AcOEt 7:3) afforded the title compound (2*S*,3*R*)-**9** as a pale yellow solid (93.2 mg, 83%).

Enantiomeric excess (98% ee) was determined by chiral HPLC analysis under the following conditions: 0.5 mg in 1 mL *i*-PrOH/EtOH 1:1, Chiralpak AD column, hexane/*i*-PrOH 70:30, flow 0.8 mL/min, λ 220 nm, t (minor) = 20.1 min, t (major) = 26.5 min.

^{1}H NMR (DMSO-d_6, 400 MHz): δ 9.10 (1H, d, *J* = 9.0 Hz, N<u>H</u>), 8.15 (2H, d, *J* = 9.0 Hz, C_{ArNO2}<u>H</u>), 7.72 (2H, d, *J* = 9.0 Hz, C_{ArNO2}<u>H</u>), 7.50-7.22 (10H, m, Ph), 6.86 (1H, s, C<u>H</u>Ph_2), 6.59 (1H, s, C<u>H</u>Cl_2), 6.50 (1H, d, *J* = 4.5 Hz, O<u>H</u>), 5.49 (1H, t, *J* = 4.0 Hz, ArC<u>H</u>OH), 4.89 (1H, dd, *J* = 9.0, 3.0 Hz, C<u>H</u>NH).

^{13}C NMR (DMSO-d_6, 100 MHz): δ 168.0 (<u>C</u>O_2), 163.9 (<u>C</u>ONH), 149.0 (<u>C</u>$_{ArNO2}$), 146.8 (<u>C</u>$_{ArNO2}$), 140.3 (<u>C</u>$_{Ar}$), 140.2 (<u>C</u>$_{Ar}$), 128.5 (<u>C</u>$_{Ar}$H), 128.4 (<u>C</u>$_{Ar}$H), 127.9 (<u>C</u>$_{Ar}$H), 127.7 (<u>C</u>$_{Ar}$H), 127.6 (<u>C</u>$_{Ar}$H), 126.6 (<u>C</u>$_{Ar}$H), 126.3 (<u>C</u>$_{ArNO2}$H), 123.0 (<u>C</u>$_{ArNO2}$H), 77.6 (<u>C</u>HPh_2), 71.2 (Ar<u>C</u>HOH), 65.8 (<u>C</u>HCl_2), 58.4 (<u>C</u>HNH).

HRMS (ESI, MeOH): calcd. for $[M+Na]^+$ ($C_{24}H_{20}Cl_2N_2NaO_6$) 525.0591, found 525.0588.

M.p.: 130-136 °C.

IR: 3552 (w, br), 3332 (m, br), 2854 (w), 1741 (s, OC=O stretch), 1683 (s, NC=O stretch), 1543 (m), 1506 (s), 1454 (w), 1344 (s), 1299 (m), 1274 (m), 1203 (s), 1107 (m), 1068 (m), 1013 (w), 974 (w), 954 (w), 852 (w), 812 (m), 758 (s), 696 (s).

$[\alpha]_D^{25}$: –0.85 (c 1.00, AcOEt, sample with 87% ee).

Diphenylmethyl (2*R*,3*S*)-2-(2,2-dichloroacetamido)-3-hydroxy-3-(4-nitrophenyl)propanoate (2*R*,3*S*)-(9)

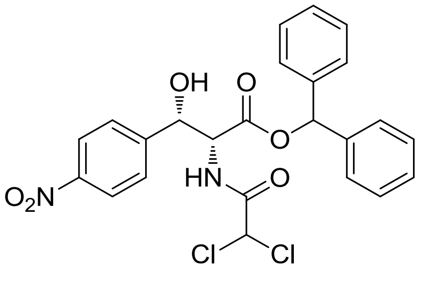

Synthesized and purified in identical fashion starting from (2*R*,3*S*)-**5**. The title compound (2*R*,3*S*)-**9** was obtained as pale yellow solid (82%). Enantiomeric excess (93% ee) was determined by chiral HPLC analysis under the following conditions: 0.5 mg in 1 mL *i*-PrOH/EtOH 1:1, Chiralpak AD column, hexane/*i*-PrOH 70:30, flow 0.8 mL/min, λ 220 nm, t (major) = 20.3 min, t (minor) = 26.9 min.

$[\alpha]_D^{25}$: +0.84 (c 1.00, AcOEt, sample with 93% ee).

(±)-**9** was prepared in an identical fashion from (±)-**5**.

2.7. Synthesis and characterisation of (–)-chloramphenicol and (+)chloramphenicol (1)

(−)-Chloramphenicol

2,2-Dichloro-*N*-[(1*R*,2*R*)-1,3-dihydroxy-1-(4-nitrophenyl)propan-2-yl]acetamide (−)-(1)

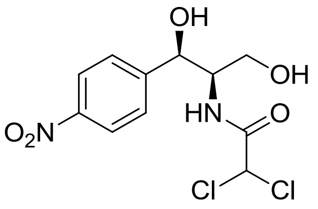

Sodium borohydride (28.2 mg, 0.745 mmol, 5.0 eq) was added in one portion to a stirred suspension of (2*S*,3*R*)-**9** (75.0 mg, 0.149 mmol, 1.0 eq) in methanol (0.8 mL) at 0 °C. The reaction mixture was stirred at 0 °C for 1 hour, then quenched by adding H_2O (15 mL). AcOEt (15 mL) was added and the phases separated. The aqueous layer was extracted with AcOEt (3 × 10 mL). The combined organic phases were dried over Na_2SO_4, filtered and evaporated. Purification of the crude mixture by FCC (PE/AcOEt 3:7 to 2:8) afforded the title compound **(−)-1** as a pale yellow solid (38.5 mg, 80%).

Enantiomeric excess (99%) was determined by chiral HPLC analysis under the following conditions: 0.5 mg in 1 mL *i*-PrOH/EtOH 1:1, Chiralpak AD-H column, hexane/*i*-PrOH 90:10, flow 1.0 mL/min, λ 220 nm, t (major) = 11.5 min, t (minor) = 19.7 min.

Spectroscopic data are in agreement with those reported in the literature.[12,13]

^{1}H NMR (acetone-d_6, 500 MHz): δ 8.19 (2H, d, *J* = 9.0 Hz, $C_{ArNO2}\underline{H}$), 7.71 (2H, d, *J* = 8.5 Hz, $C_{ArNO2}\underline{H}$), 7.52 (1H, d, *J* = 8.0 Hz, N$\underline{H}$), 6.36 (1H, s, C$\underline{H}$$Cl_2$), 5.34 (1H, m, ArC$\underline{H}$OH), 5.23 (1H, d, *J* = 4.5 Hz, ArCHO$\underline{H}$), 4.25 (1H, dd, *J* = 6.0, 5.0 Hz, $CH_2O\underline{H}$), 4.17 (1H, m, C$\underline{H}$NH), 3.81 (1H, app dt, *J* = 10.5, 6.5 Hz, $C\underline{H_A}H_BOH$), 3.71 (1H, app dt, *J* = 10.5, 5.0 Hz, $CH_A\underline{H_B}OH$).

^{13}C-NMR (acetone-d_6, 125 MHz): δ 164.4 ($\underline{C}$O), 151.4 ($\underline{C}_{ArNO2}$), 148.1 ($\underline{C}_{ArNO2}$), 128.2 ($\underline{C}_{ArNO2}$H), 123.9 ($\underline{C}_{ArNO2}$H), 71.2 (Ar$\underline{C}$HOH), 67.5 ($\underline{C}HCl_2$), 62.2 ($\underline{C}H_2OH$), 57.9 ($\underline{C}$HNH).

M.p.: 150-151 °C. Lit.: 149.7-150.7 °C;[14] 150-151 °C;[15] 150.5-151.5 °C.[16]

$[\alpha]_D^{25}$: −25.1 (c 1.00, AcOEt, sample with 99% ee). Lit.: $[\alpha]_D^{23}$ = −25.5 (c = 1, AcOEt).[14]

(+)-Chloramphenicol

2,2-Dichloro-*N*-[(1*S*,2*S*)-1,3-dihydroxy-1-(4-nitrophenyl)propan-2-yl]acetamide (+)-(1)

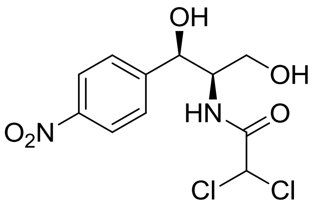

The enantiomer **(+)-1** was prepared from (2*R*,3*S*)-**9** in identical fashion, obtaining a pale yellow solid (80%). Enantiomeric excess (96%) was determined by chiral HPLC analysis under the following conditions: 0.5 mg in 1 mL *i*-PrOH/EtOH 1:1, Chiralpak AD-H column, hexane/*i*-PrOH 90:10, flow 1.0 mL/min, λ 220 nm, t (minor) = 11.8 min, t (major) = 18.8 min. $[\alpha]_D^{25}$: +24.4 (c 1.00, AcOEt, sample with 96% ee).

(±)-**1** was prepared mixing equimolar quantities (1.0 mg) of the two enantiomers.

3. ^{1}H and ^{13}C NMR spectra

(4-Methoxyphenyl)methyl 2-formamidoacetate (10d)

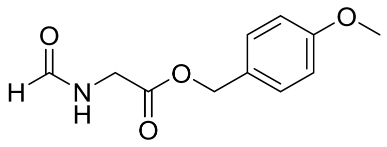

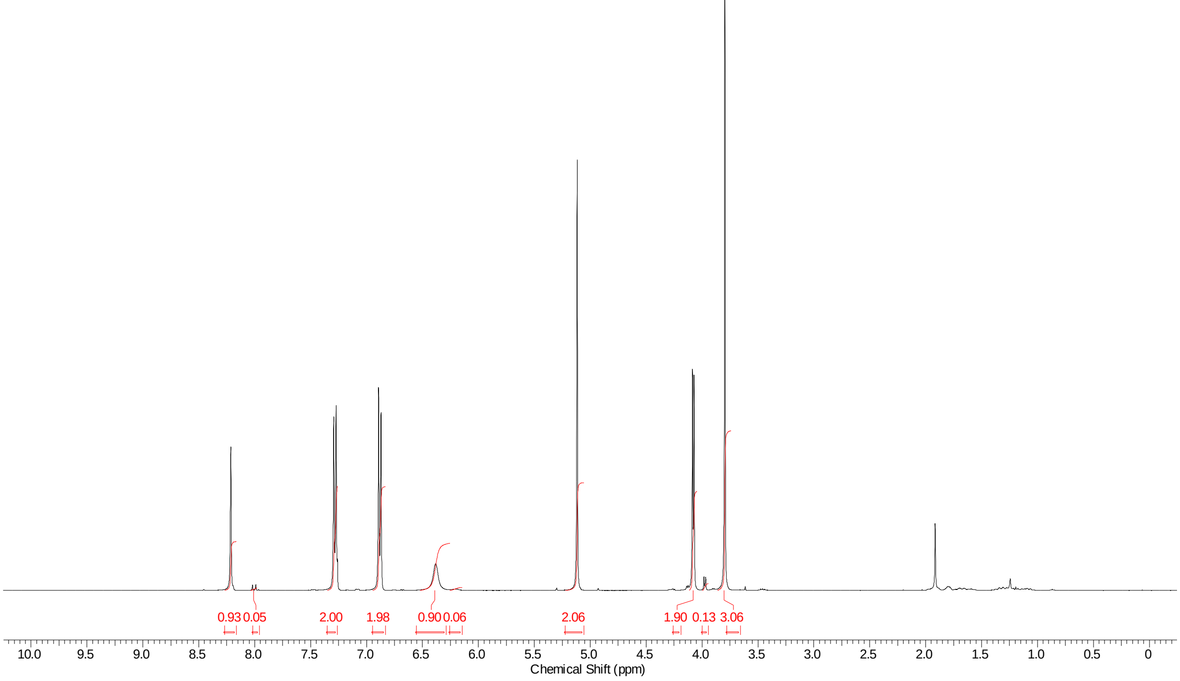

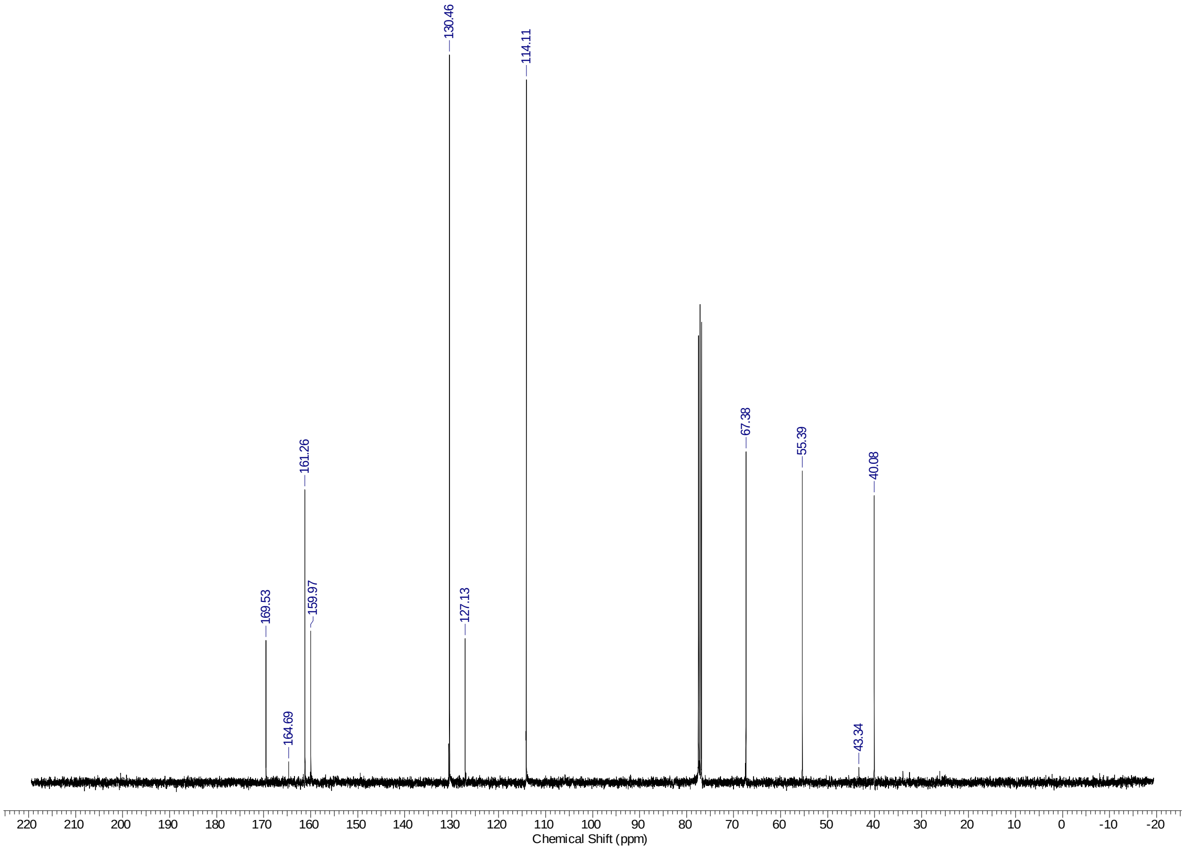

[3,5-Bis(trifluoromethyl)phenyl]methyl 2-formamidoacetate (10e)

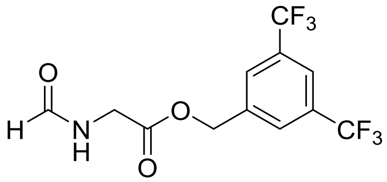

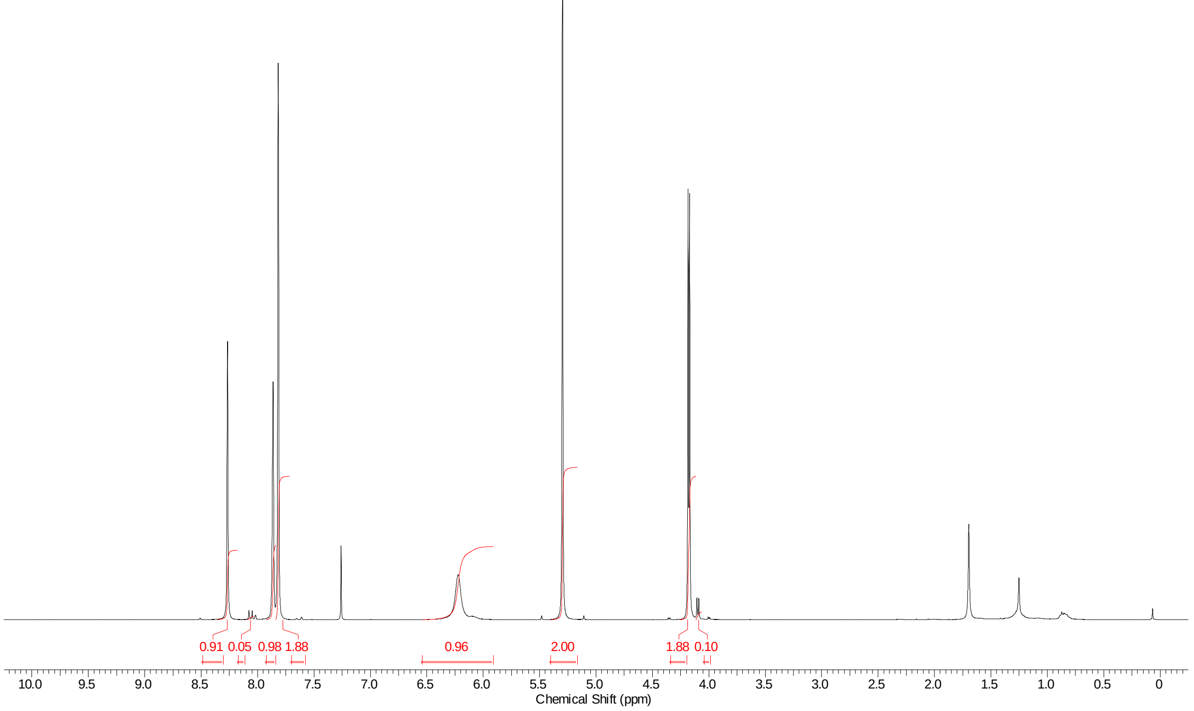

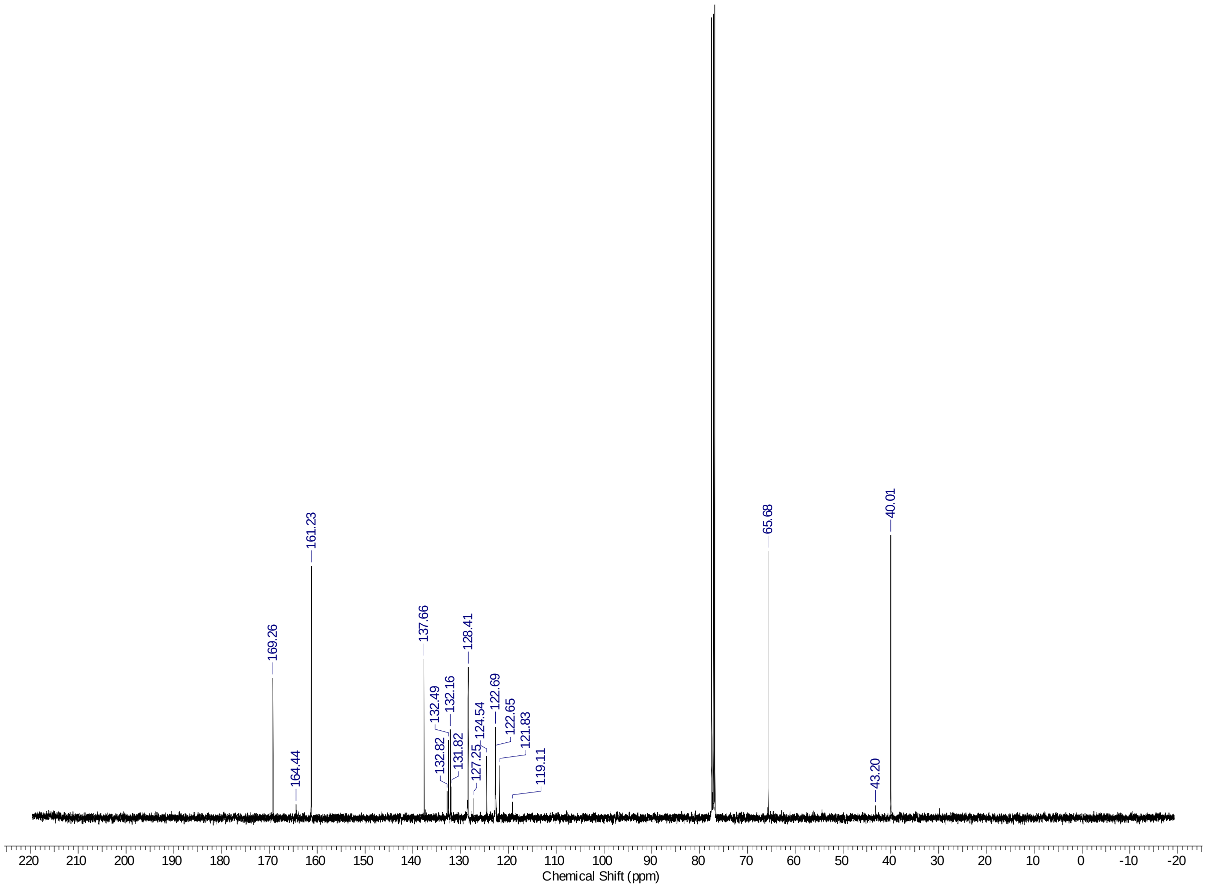

(4-Methoxyphenyl)methyl 2-isocyanoacetate (7d)

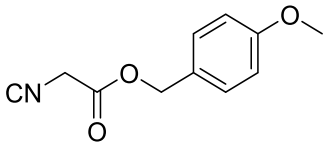

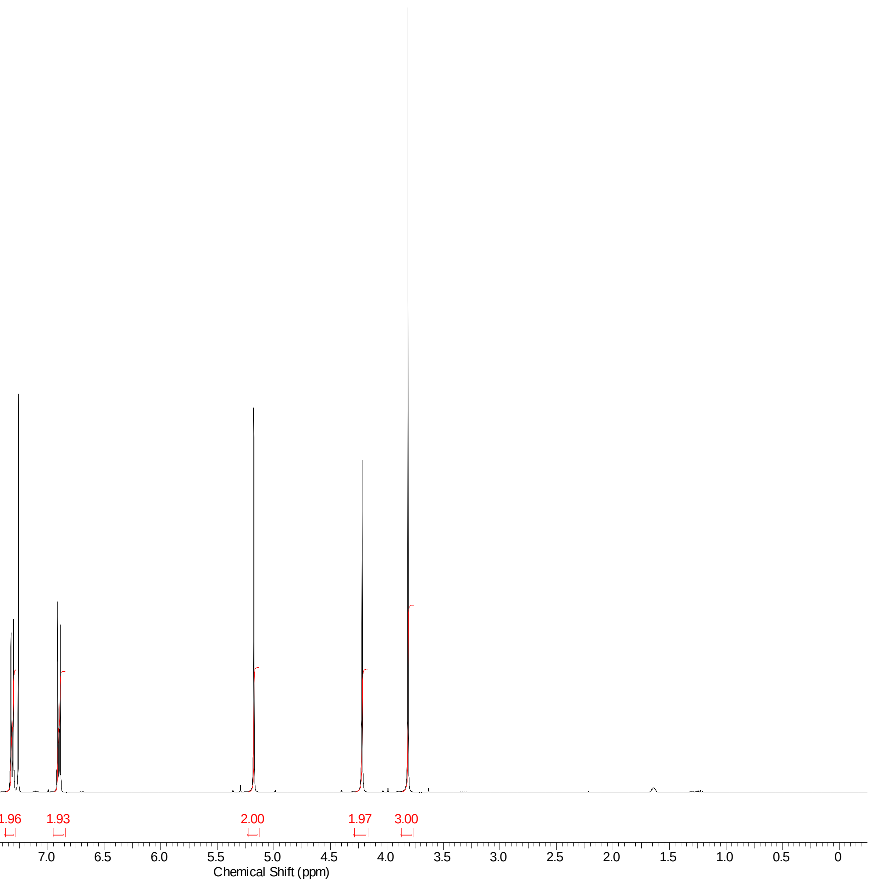

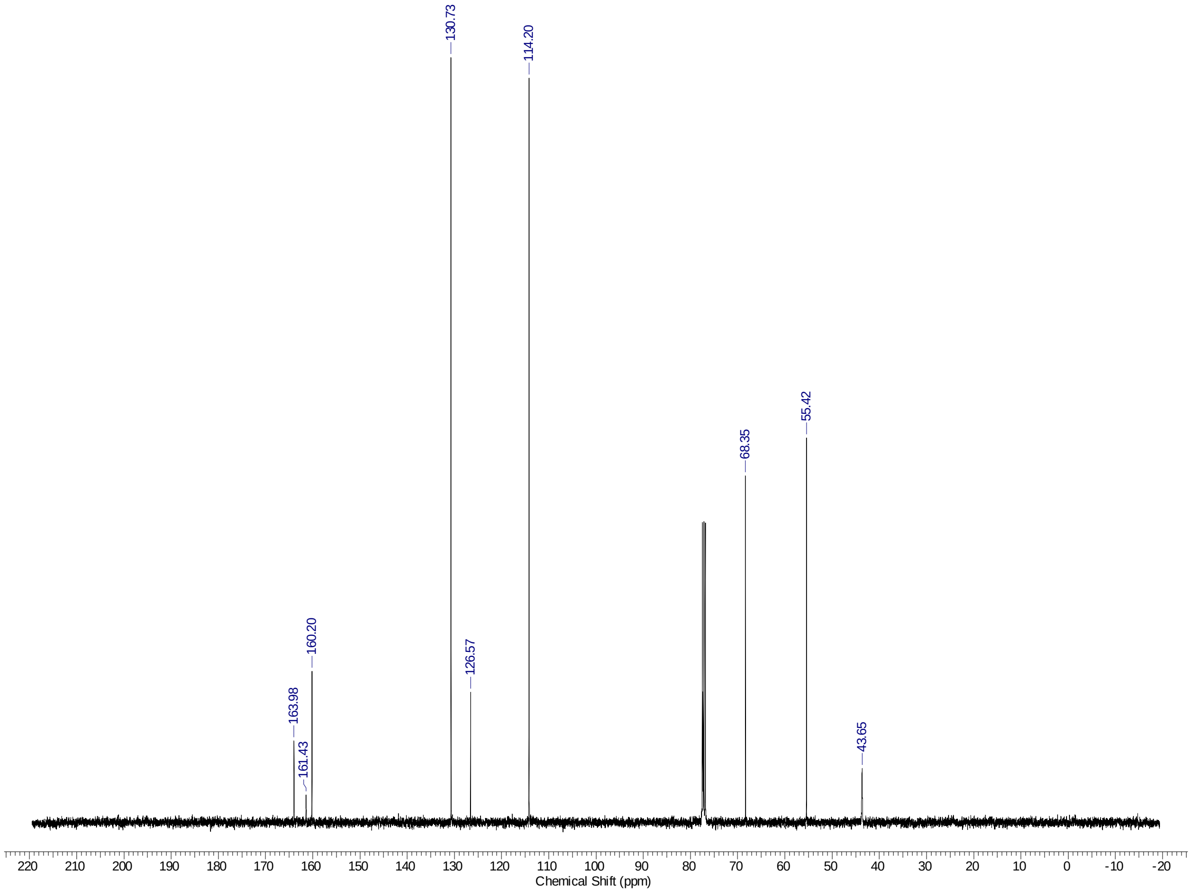

[3,5-Bis(trifluoromethyl)phenyl]methyl 2-isocyanoacetate (7e)

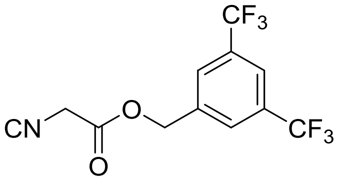

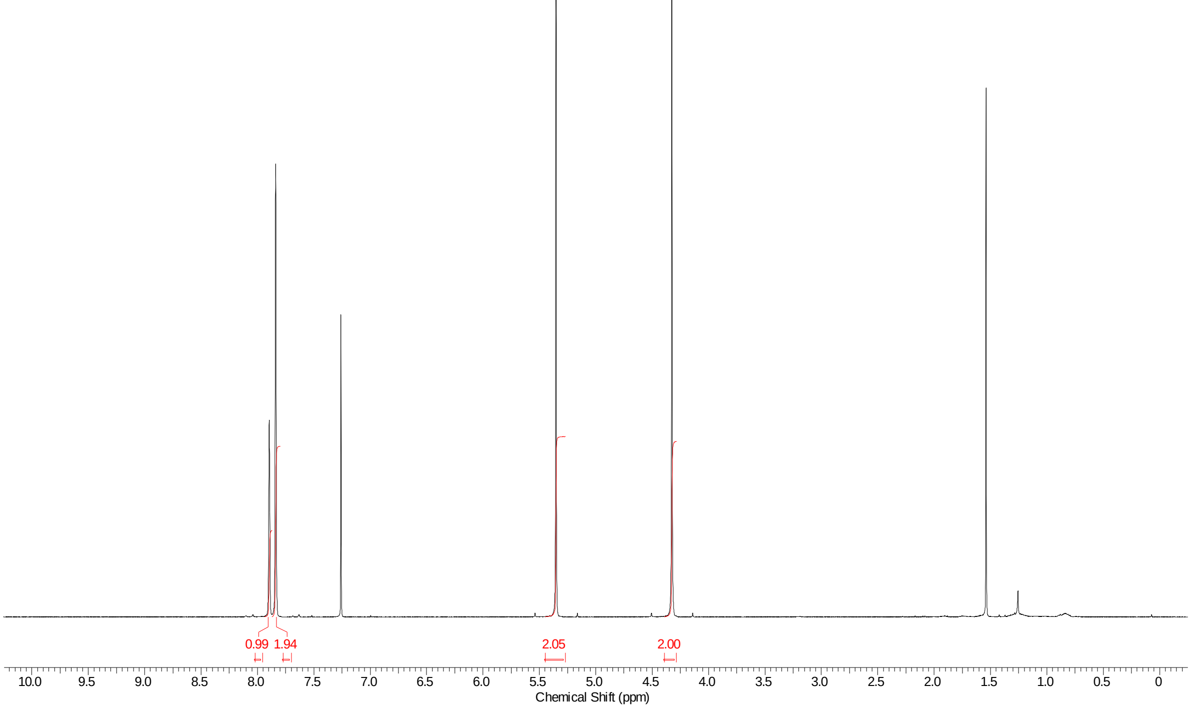

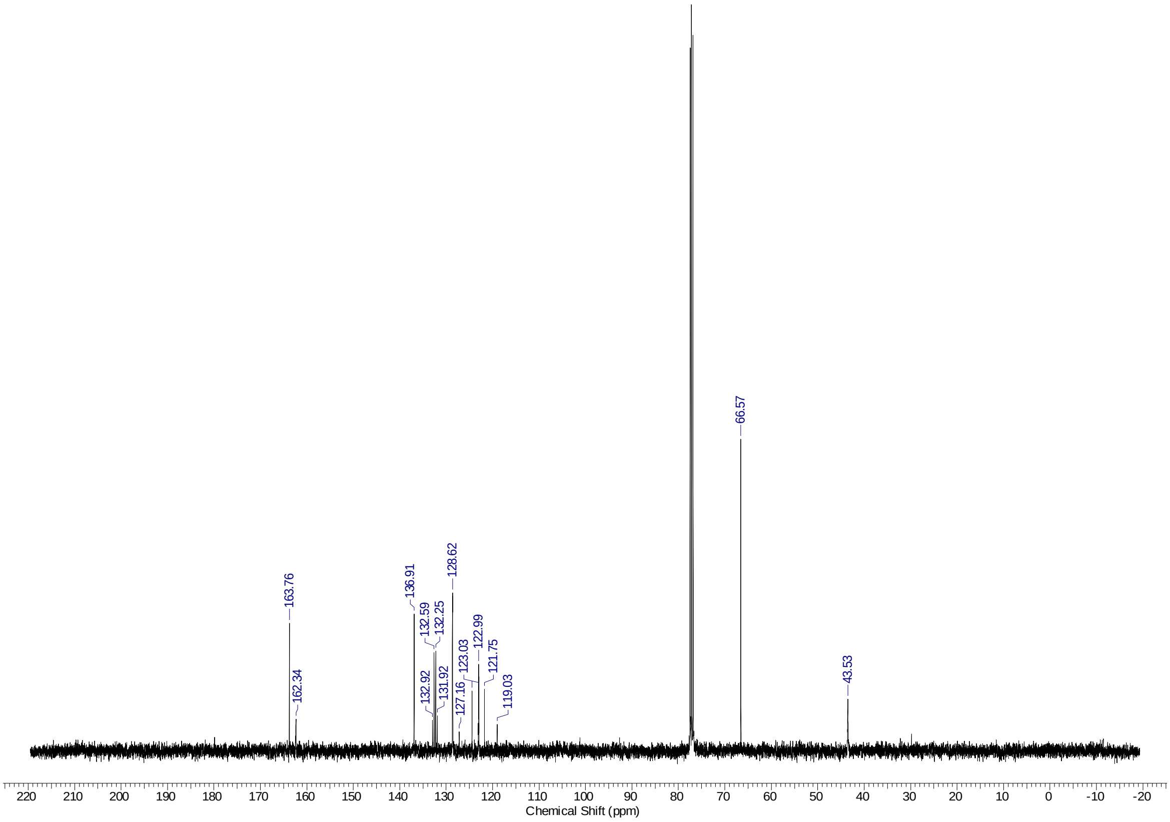

tert-Butyl (4_S_,5_R_)-5-(4-nitrophenyl)-4,5-dihydro-1,3-oxazole-4-carboxylate (6a)

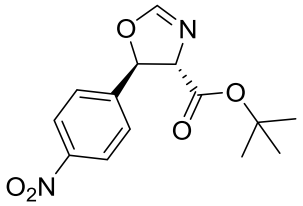

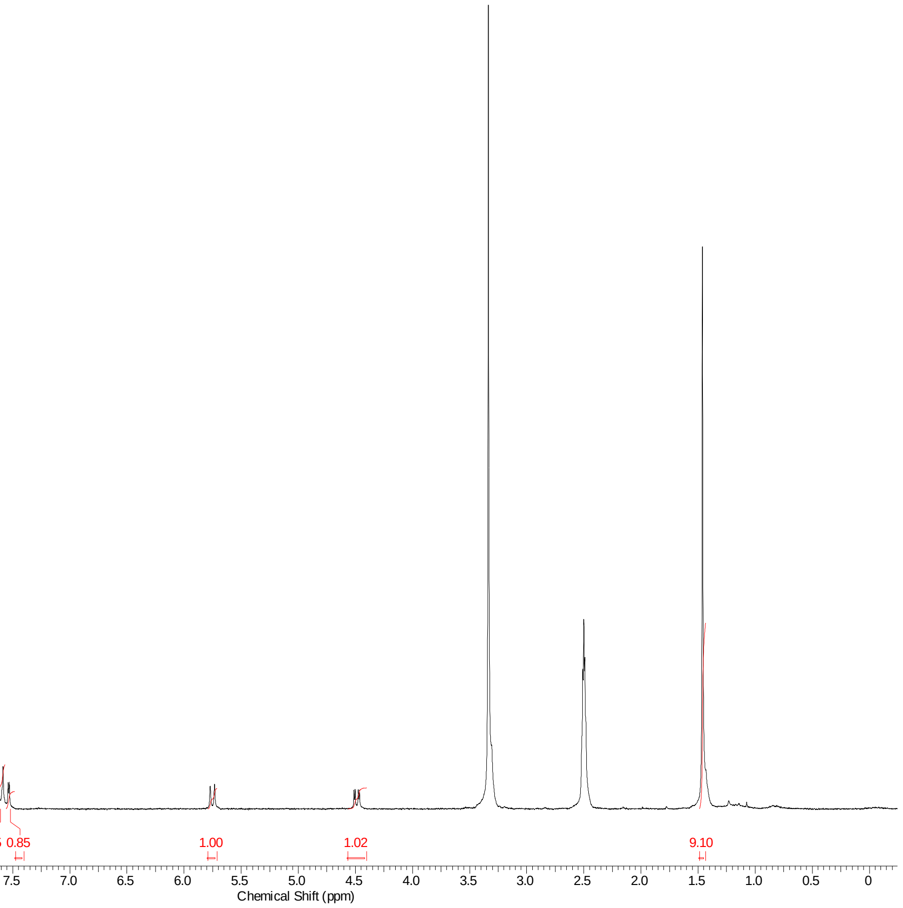

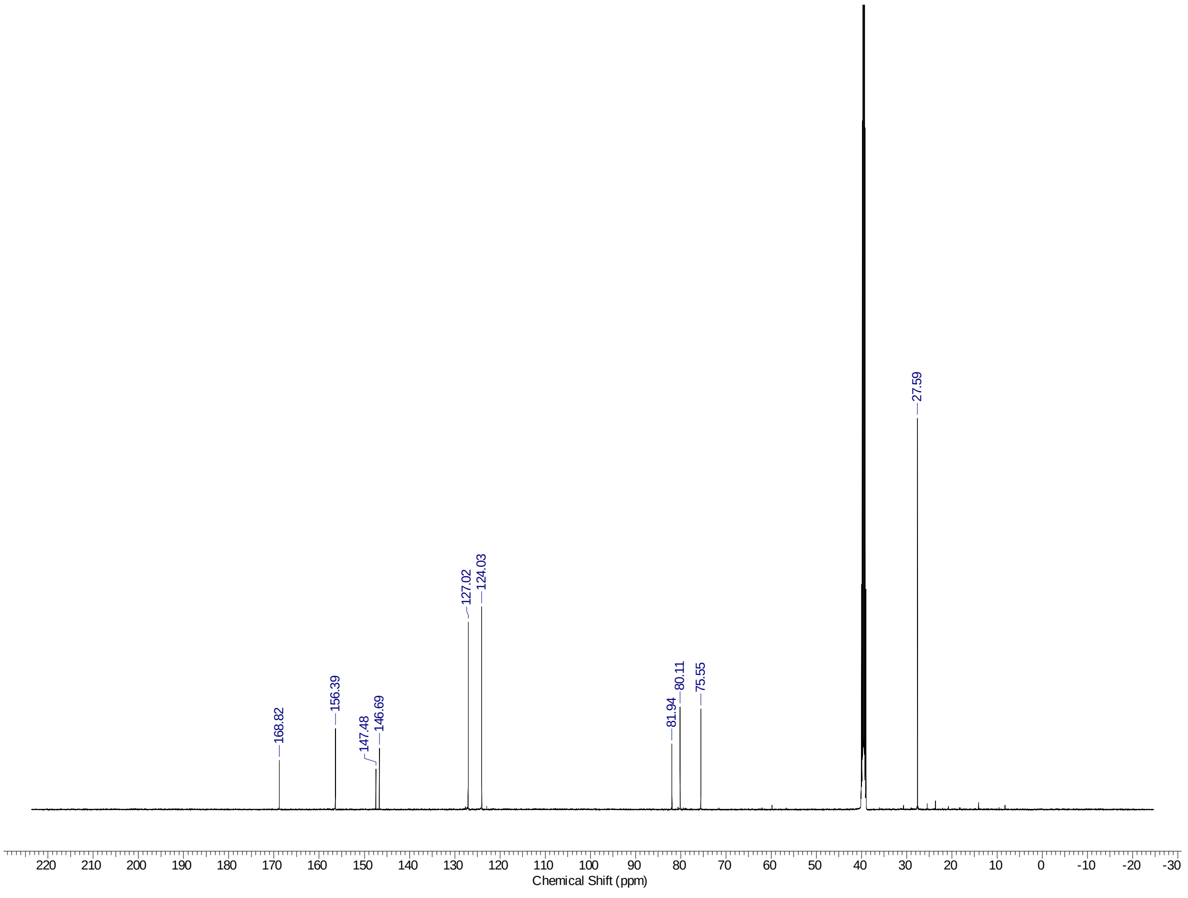

Methyl (4*S*,5*R*)-5-(4-nitrophenyl)-4,5-dihydro-1,3-oxazole-4-carboxylate (6b)

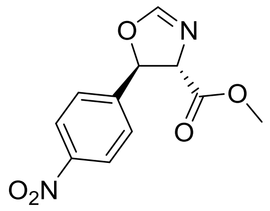

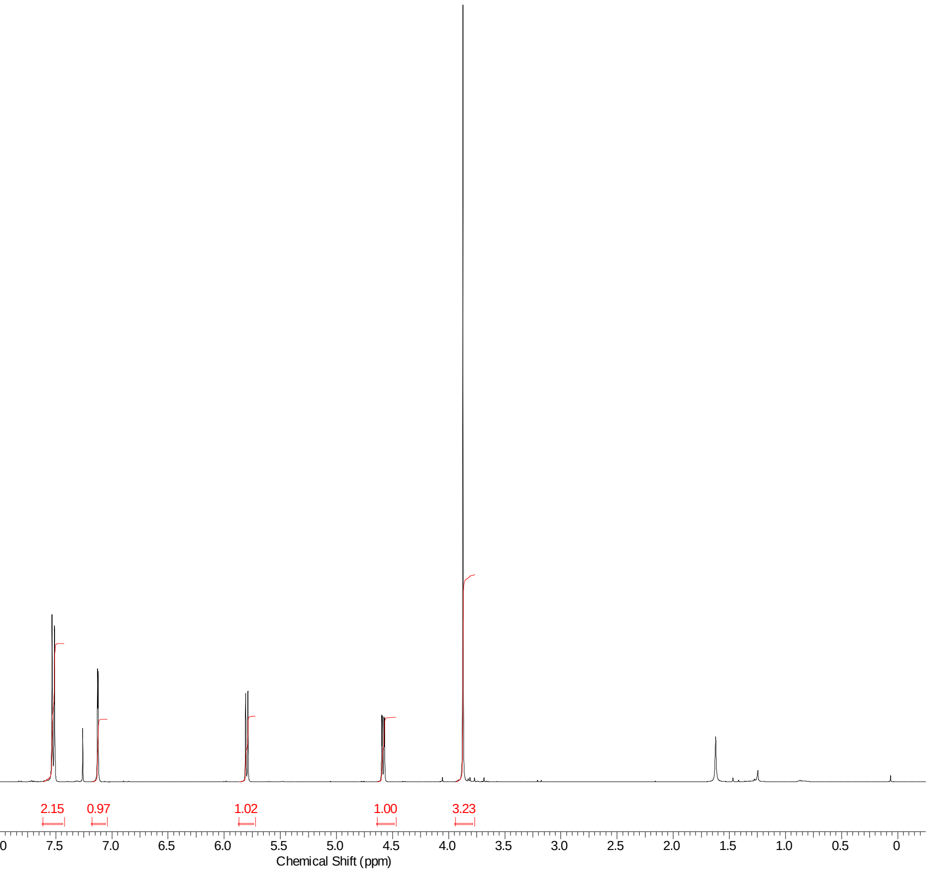

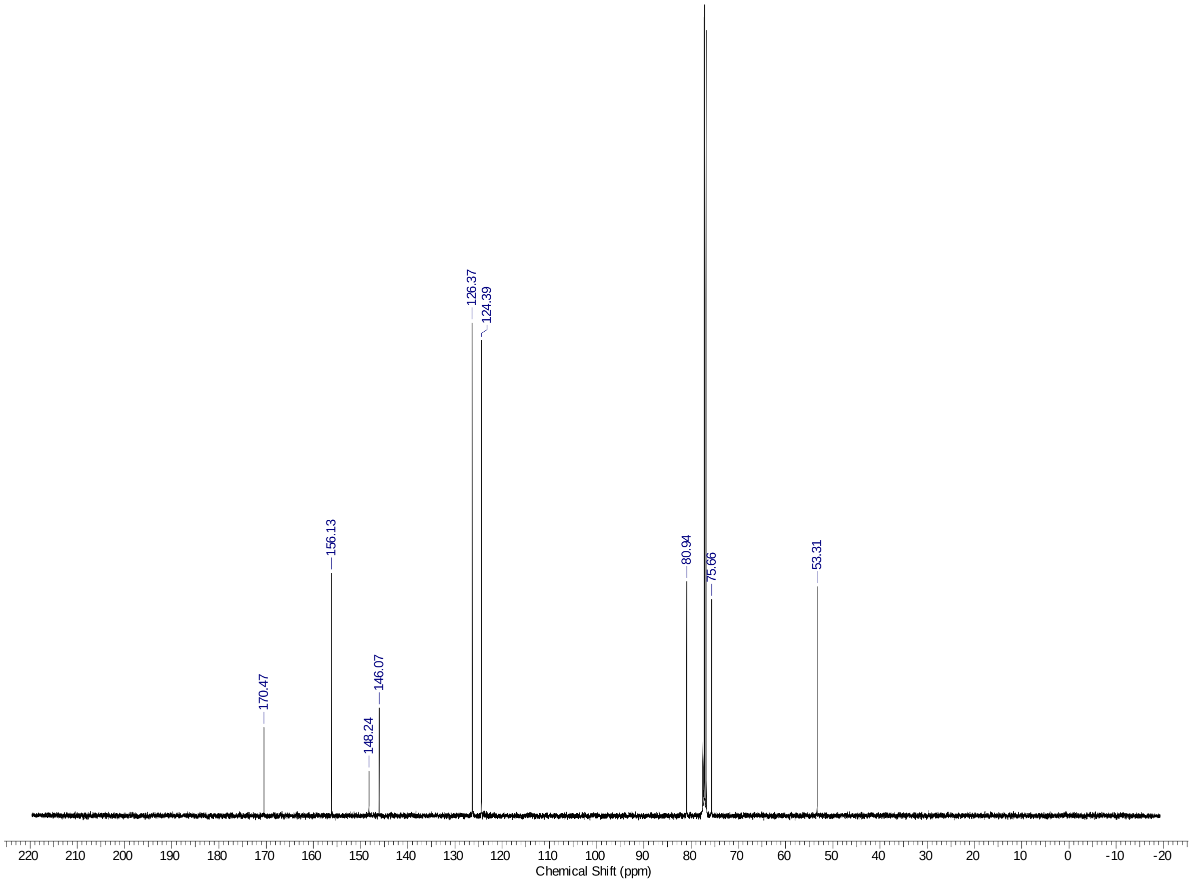

Benzyl (4*S*,5*R*)-5-(4-nitrophenyl)-4,5-dihydro-1,3-oxazole-4-carboxylate (6c)

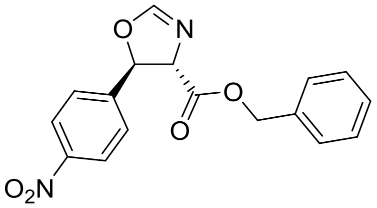

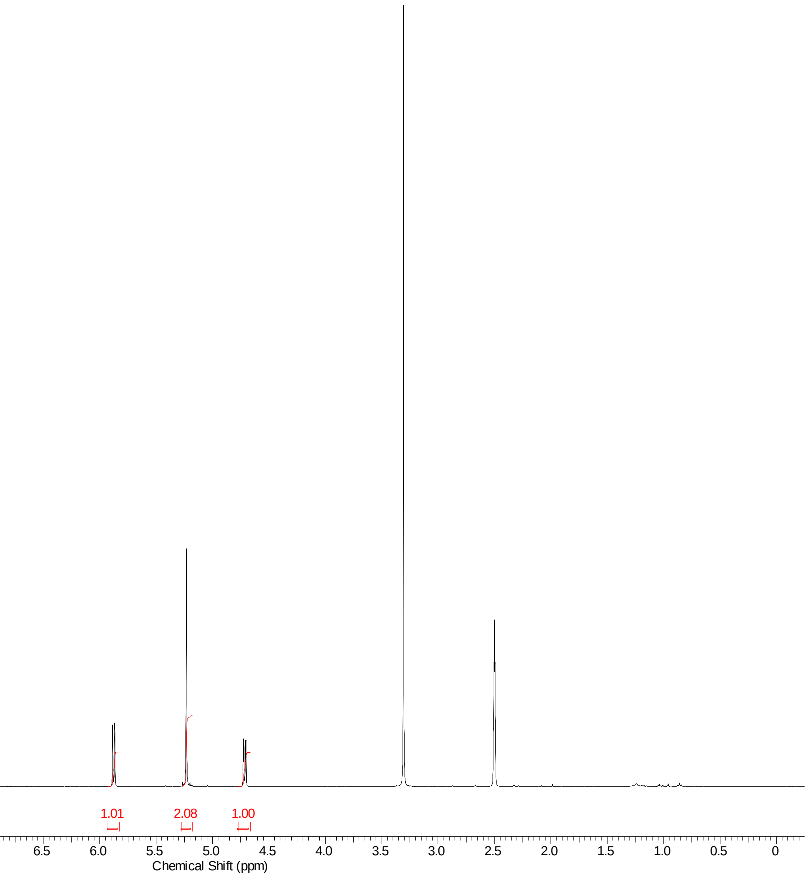

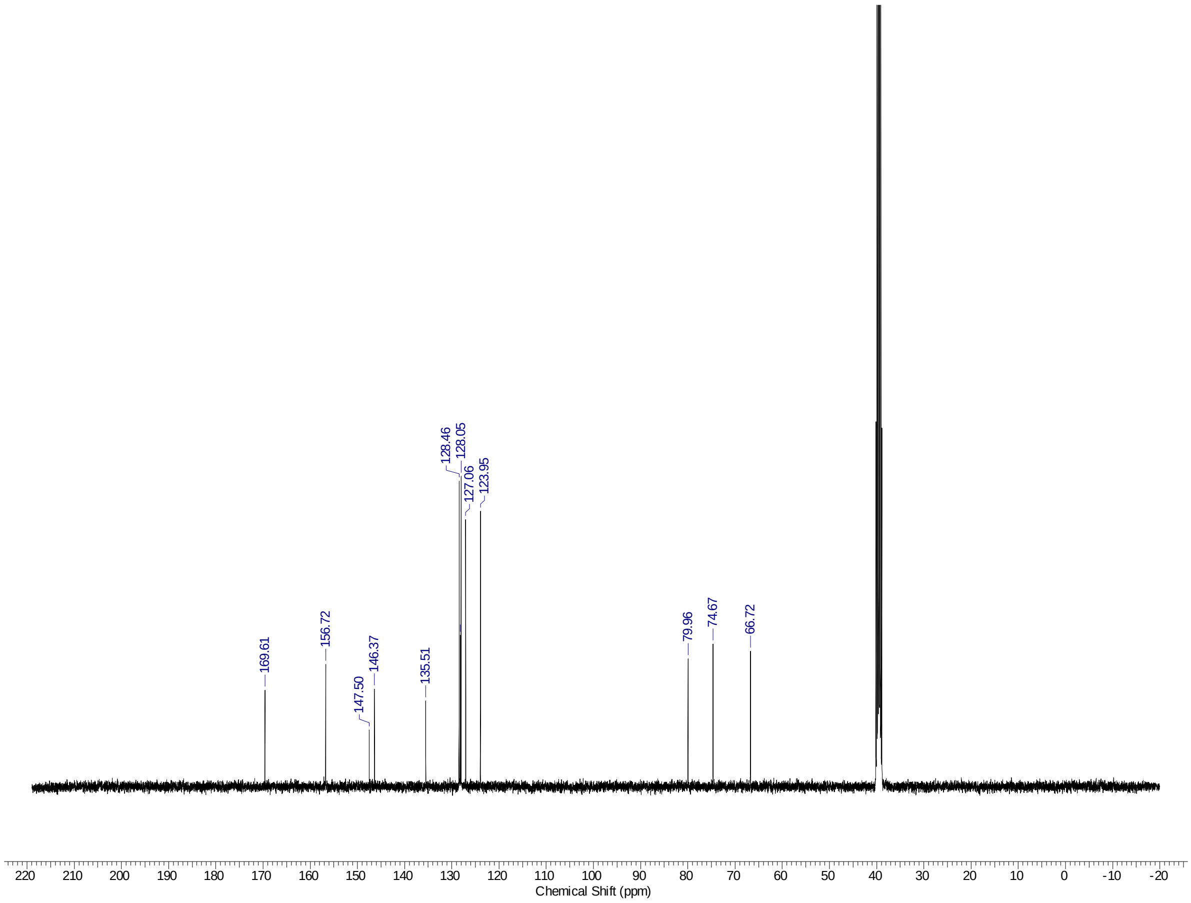

(4-Methoxyphenyl)methyl (4*S*,5*R*)-5-(4-nitrophenyl)-4,5-dihydro-1,3-oxazole-4-carboxylate (6d)

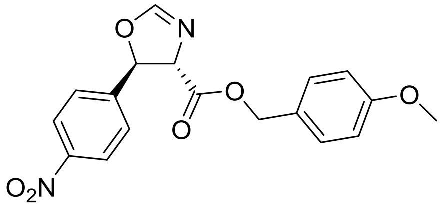

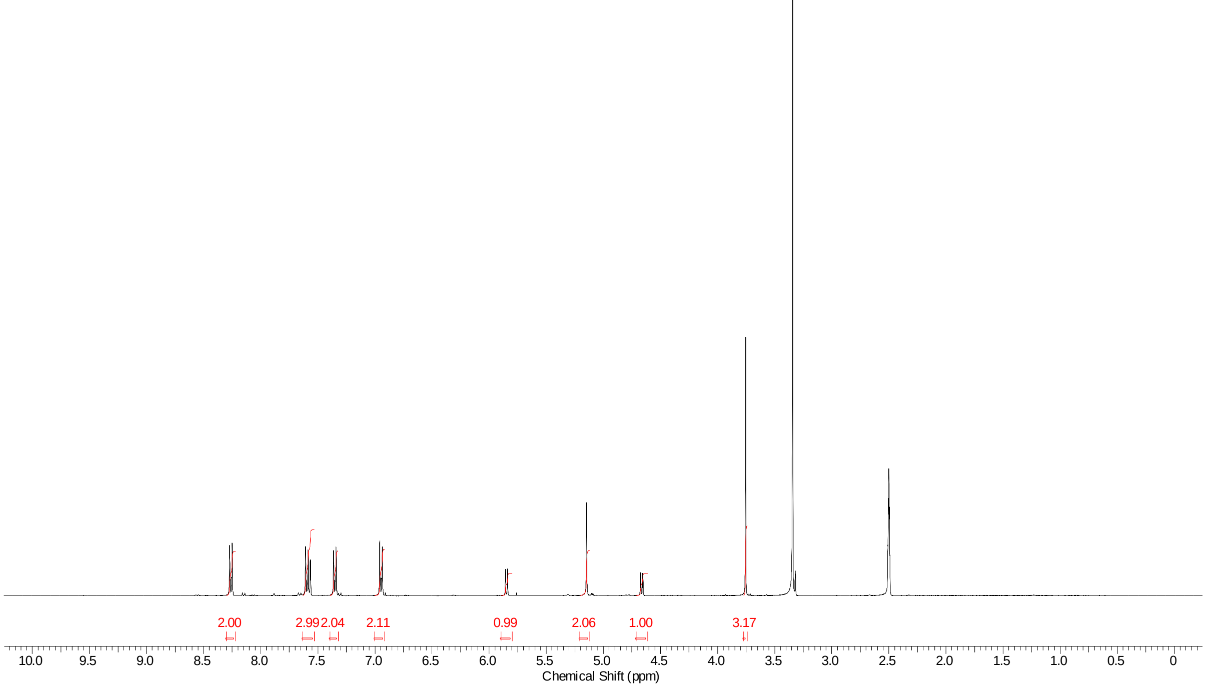

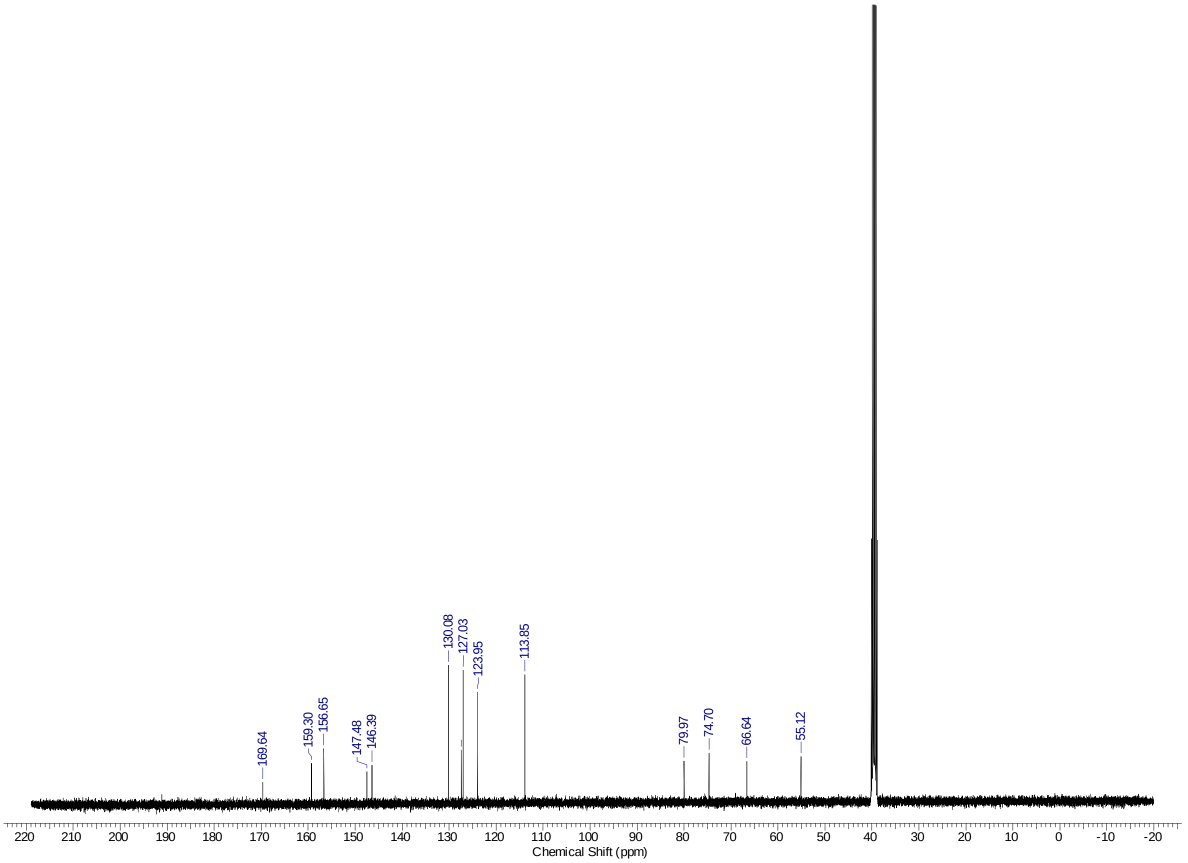

[3,5-Bis(trifluoromethyl)phenyl]methyl (4*S*,5*R*)-5-(4-nitrophenyl)-4,5-dihydro-1,3-oxazole-4-carboxylate (6e)

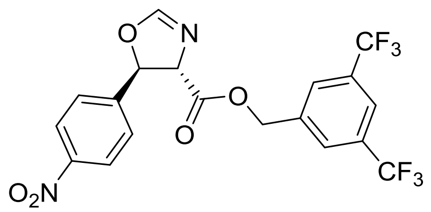

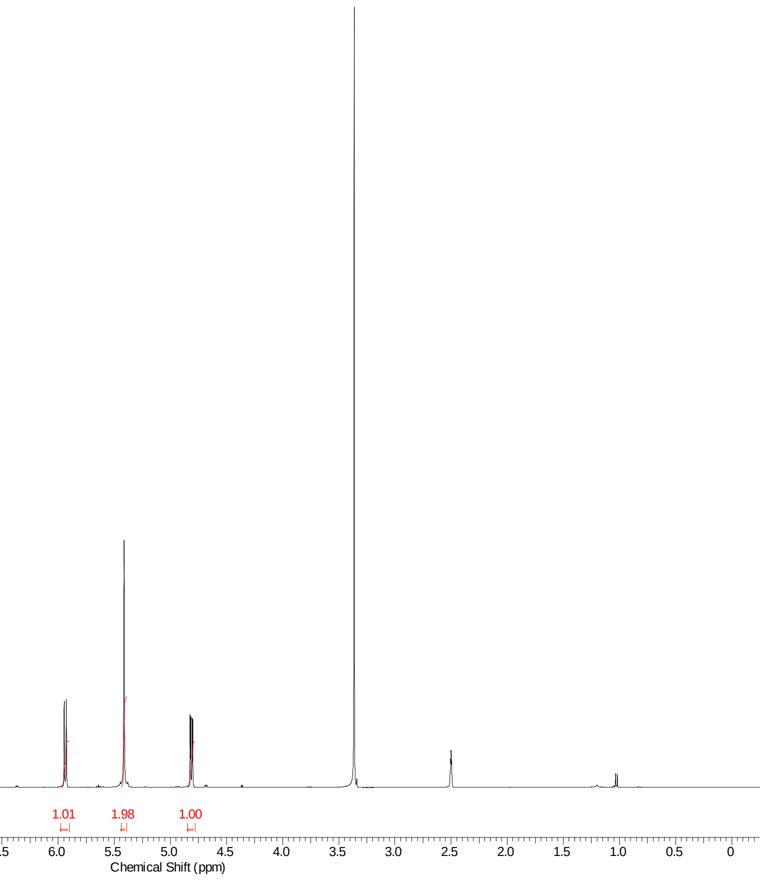

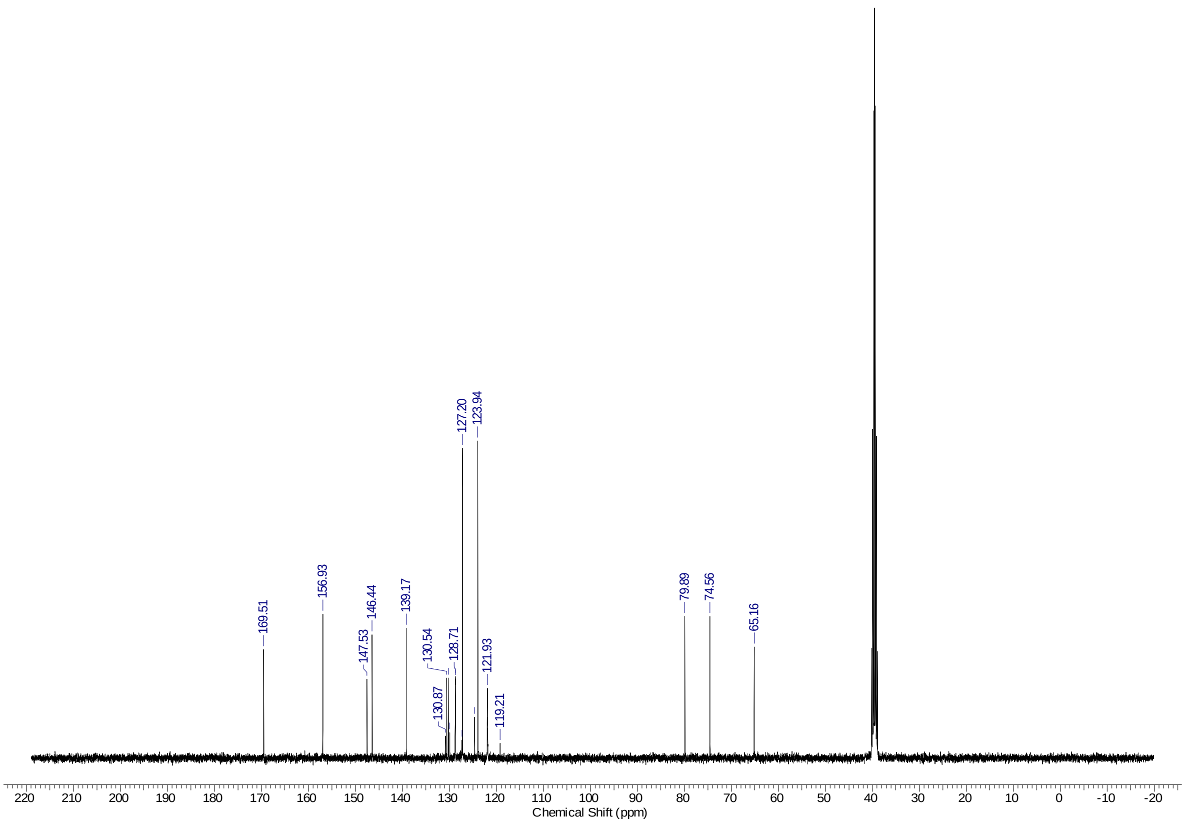

Diphenylmethyl (4*S*,5*R*)-5-(4-nitrophenyl)-4,5-dihydro-1,3-oxazole-4-carboxylate (6f)

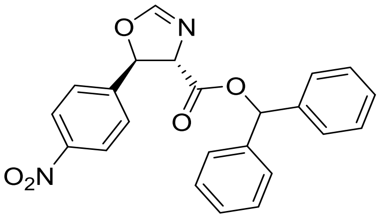

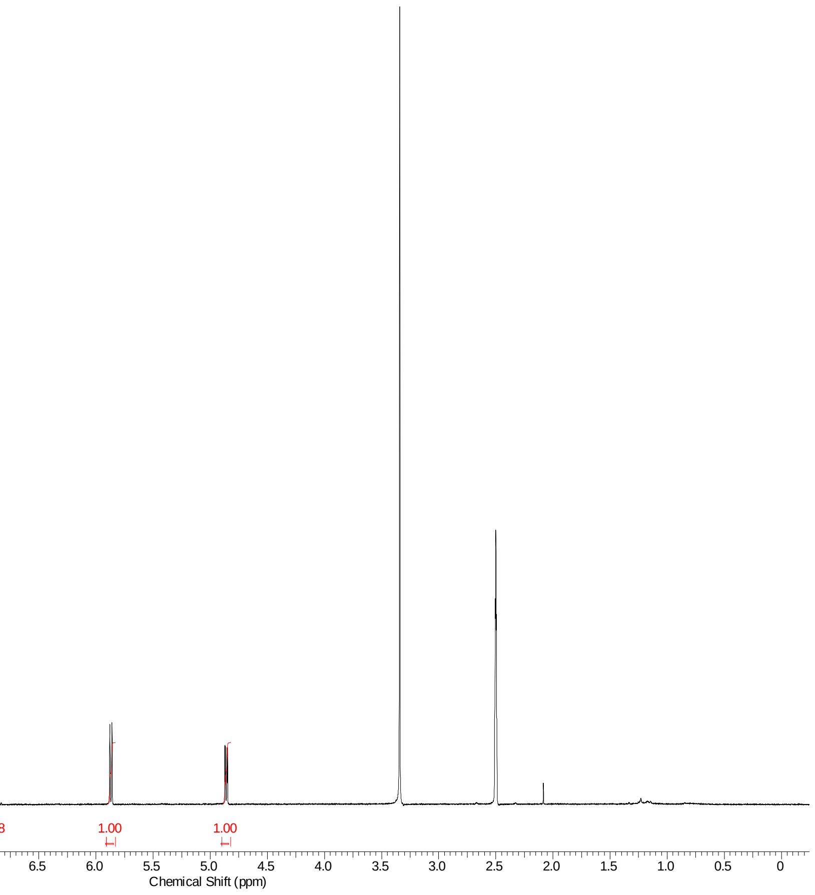

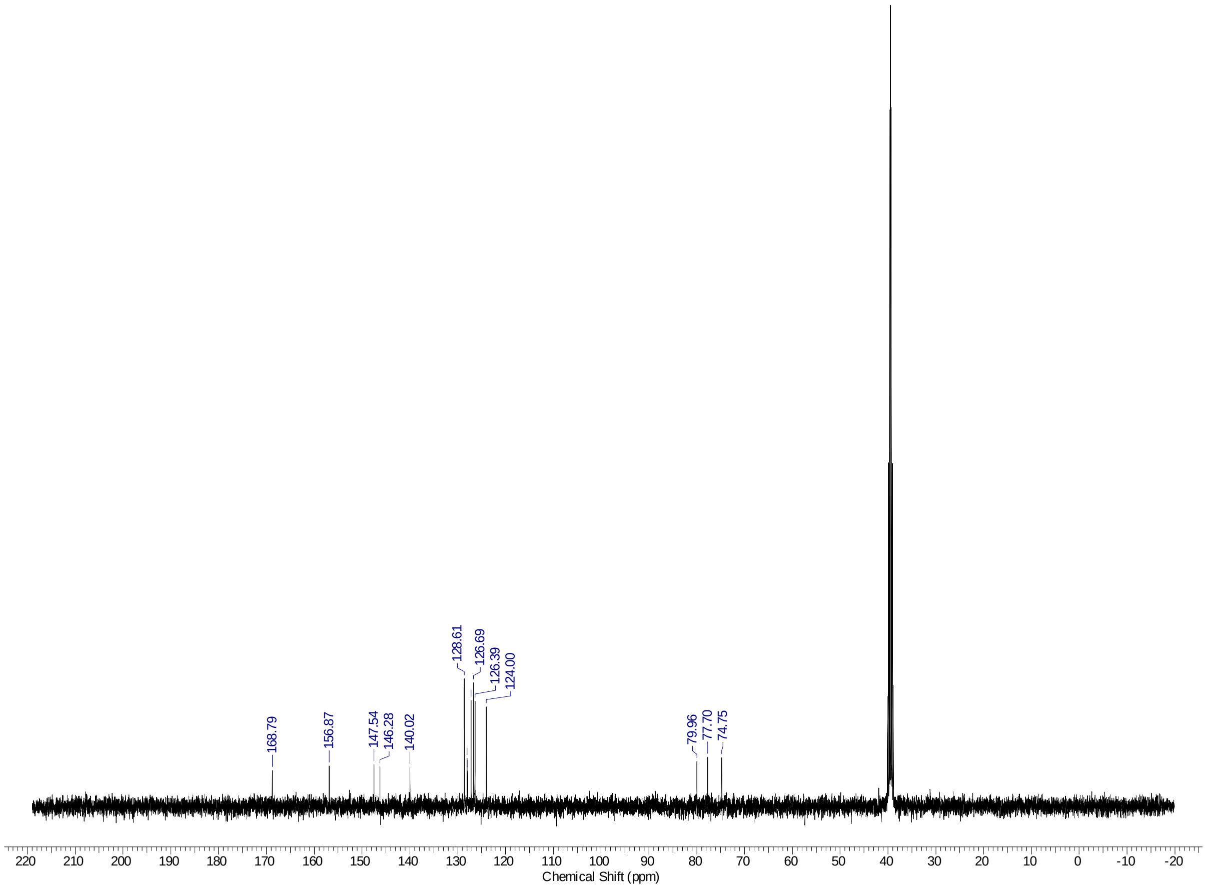

Diphenylmethyl (2*S*,3*R*)-2-amino-3-hydroxy-3-(4-nitrophenyl)propanoate (5)

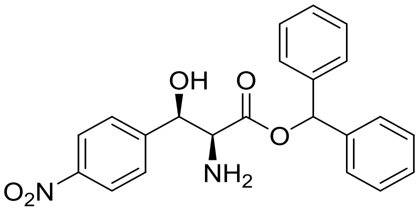

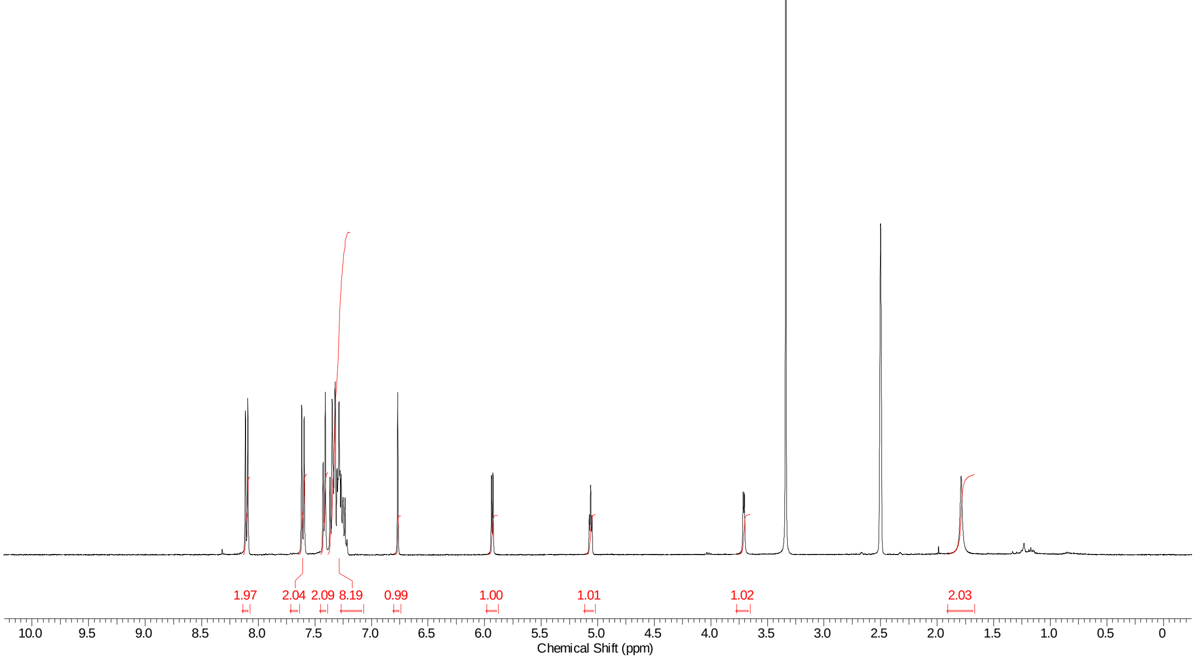

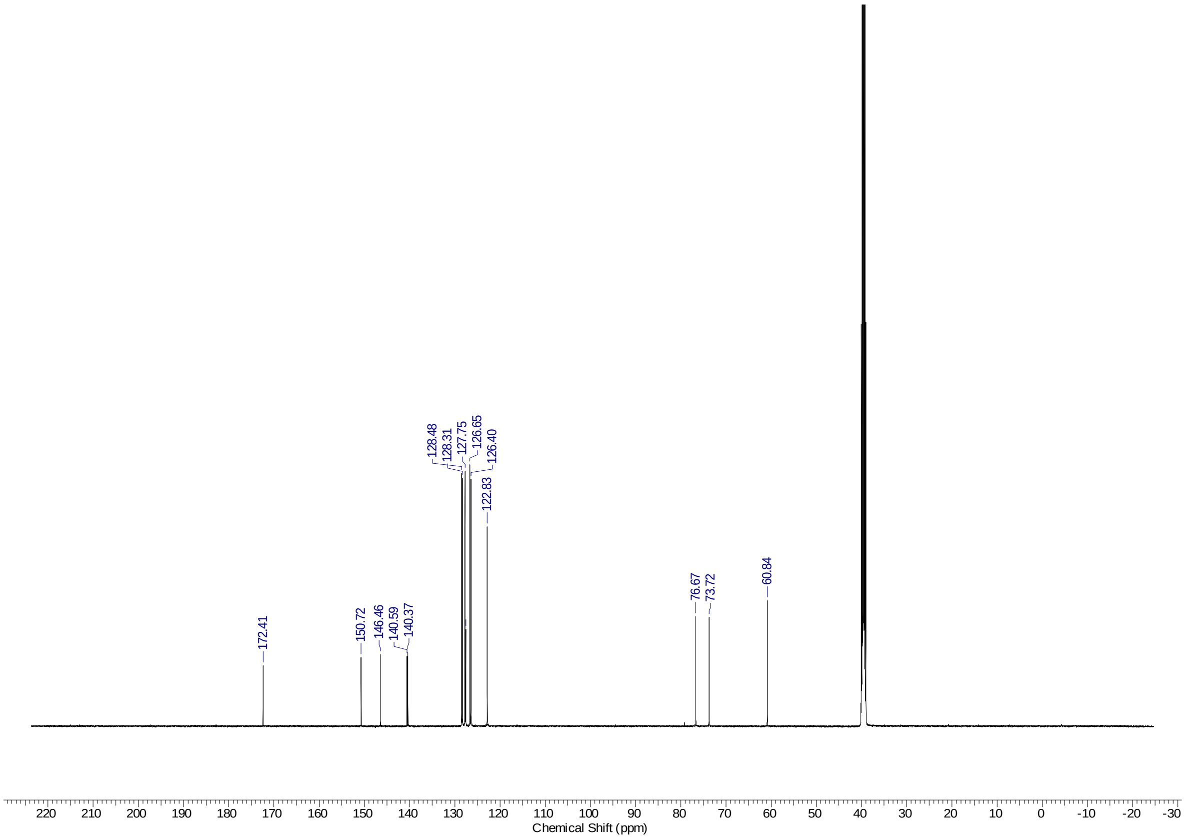

Diphenylmethyl (2*S*,3*R*)-2-(2,2-dichloroacetamido)-3-hydroxy-3-(4-nitrophenyl)propanoate (9)

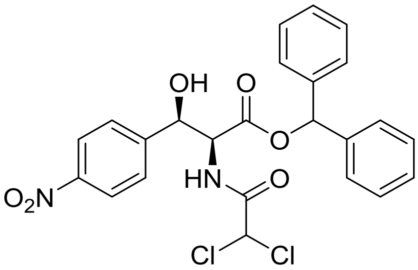

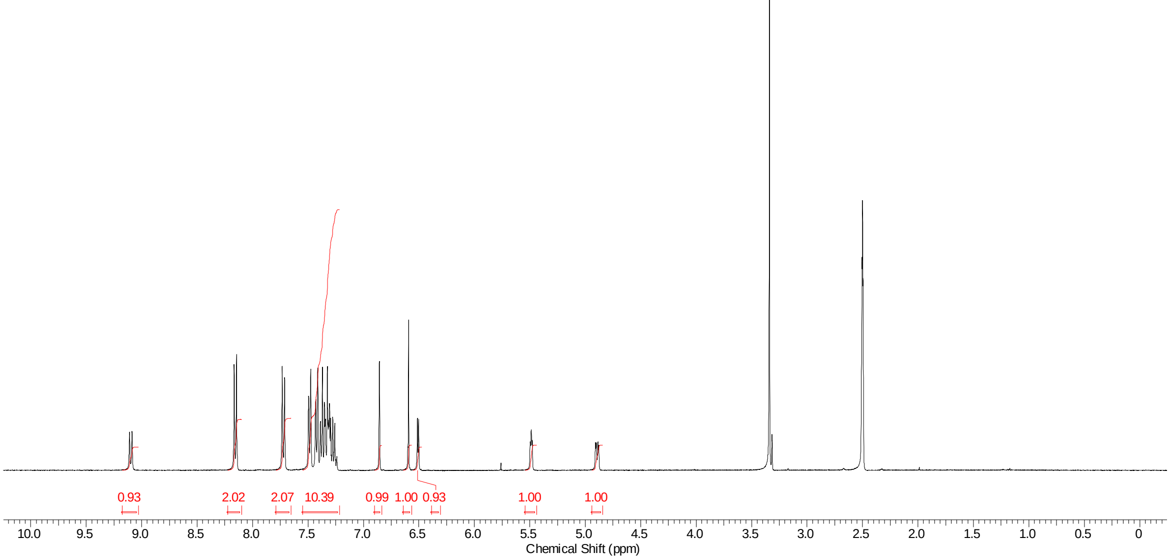

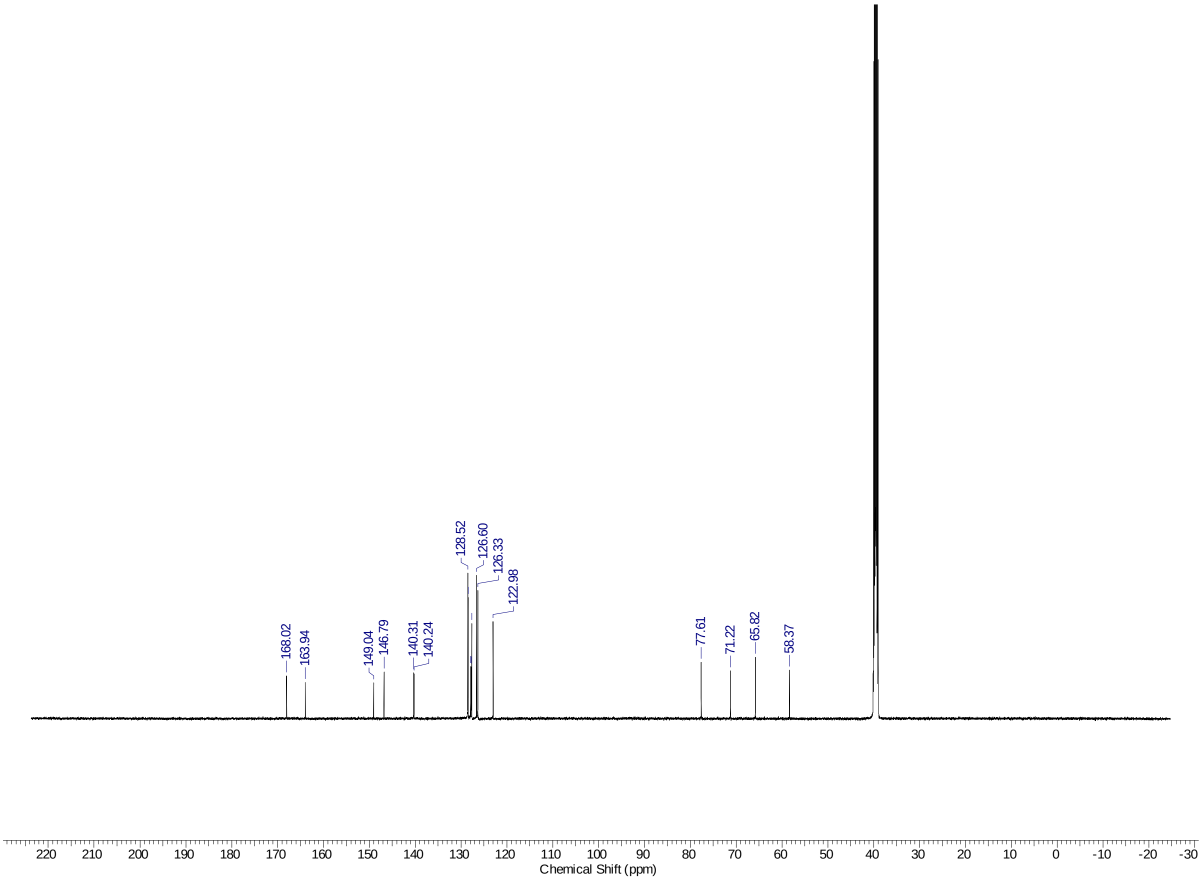

(−)-Chloramphenicol (−)-(1)

2,2-dichloro-*N*-[(1*R*,2*R*)-1,3-dihydroxy-1-(4-nitrophenyl)propan-2-yl]acetamide

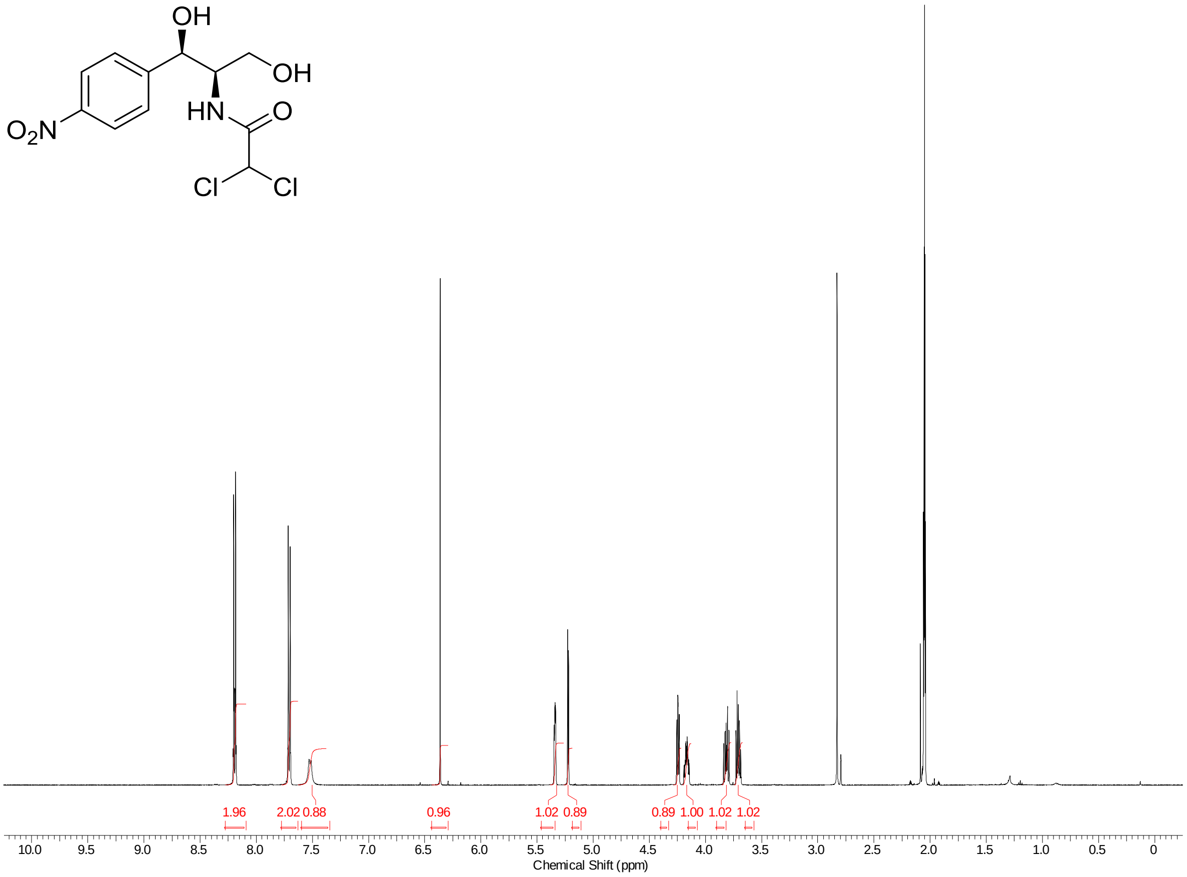

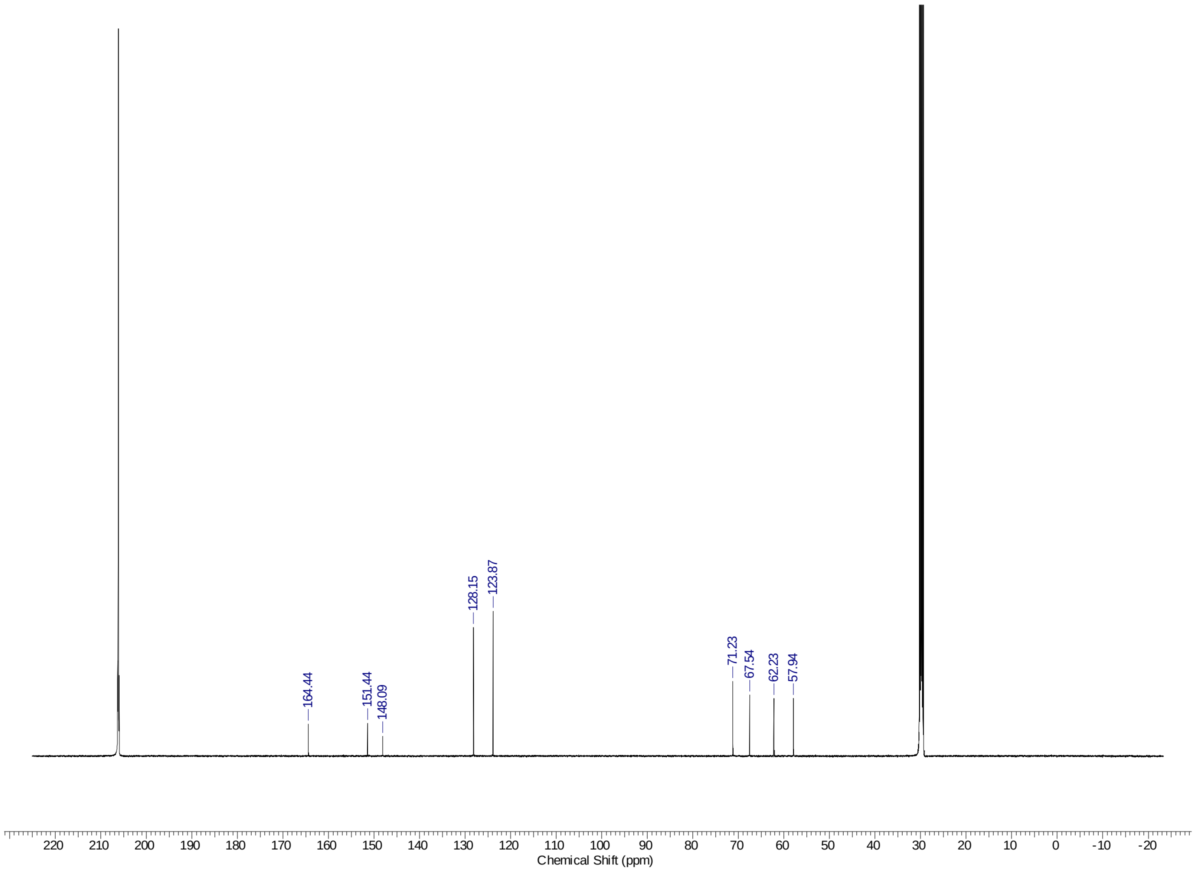

4. HPLC traces

tert-Butyl (4*S*,5*R*)-5-(4-nitrophenyl)-4,5-dihydro-1,3-oxazole-4-carboxylate (6a)

Chiralpak OD column, hexane/*i*-PrOH 90:10, 1.0 mL/min

Racemic

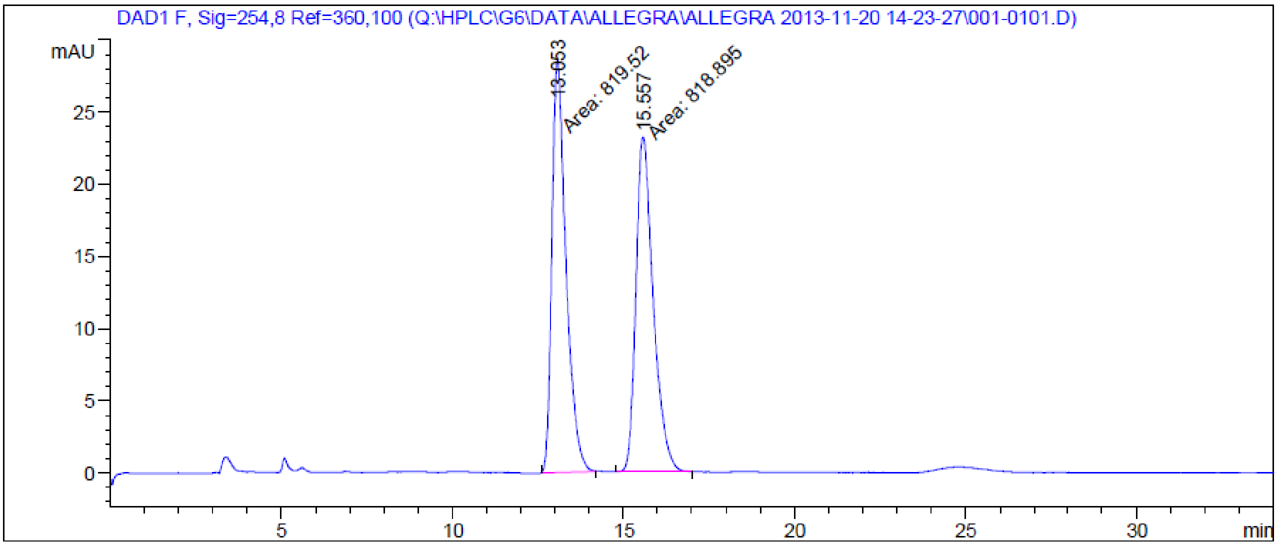

Peak #	RetTime [min]	Type	Width [min]	Area [mAU*s]	Height [mAU]	Area %
1	13.053	MM	0.4782	819.51959	28.55988	50.0190
2	15.557	MM	0.5880	818.89539	23.21058	49.9810

Enantiomerically enriched (78% ee)

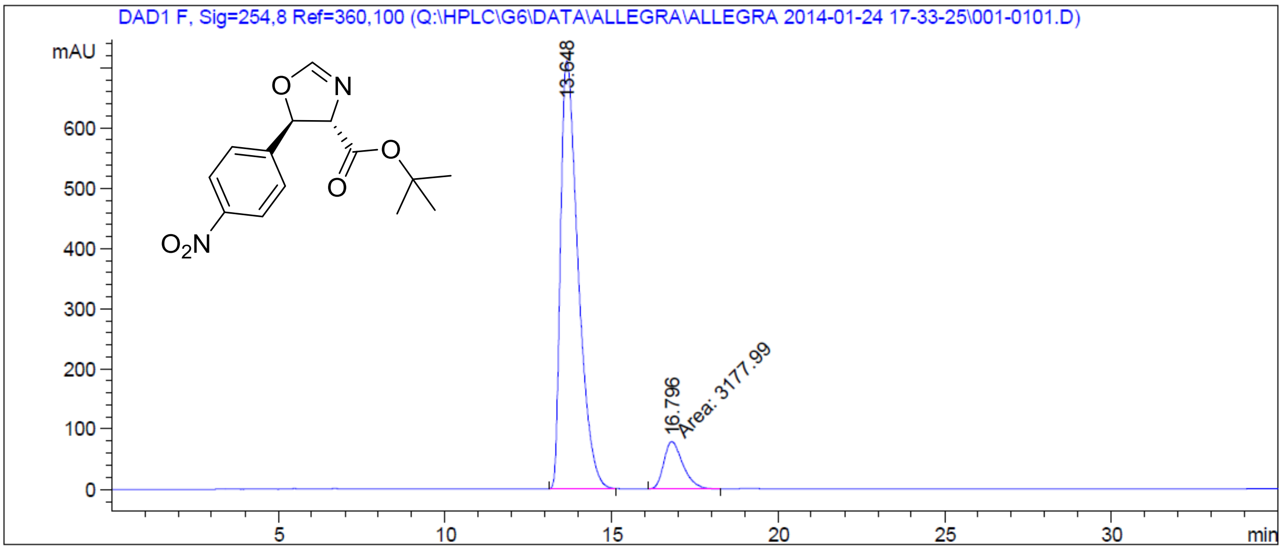

Peak #	RetTime [min]	Type	Width [min]	Area [mAU*s]	Height [mAU]	Area %
1	13.648	BB	0.5609	2.60332e4	711.43793	89.1206
2	16.796	MM	0.6717	3177.99292	78.84939	10.8794

Methyl (4*S*,5*R*)-5-(4-nitrophenyl)-4,5-dihydro-1,3-oxazole-4-carboxylate (6b)

Chiralpak AD column, hexane/*i*-PrOH 85:15, 1.0 mL/min

Racemic

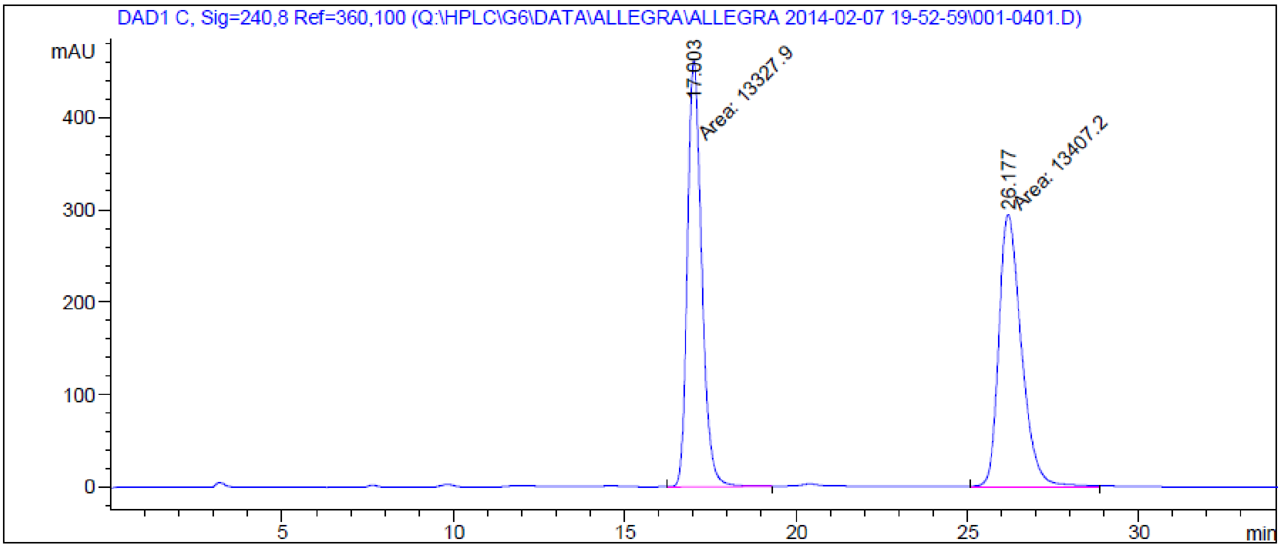

Peak #	RetTime [min]	Type	Width [min]	Area [mAU*s]	Height [mAU]	Area %
1	17.003	MM	0.4821	1.33279e4	460.78815	49.8517
2	26.177	MM	0.7596	1.34072e4	294.16309	50.1483

Enantiomerically enriched (82% ee)

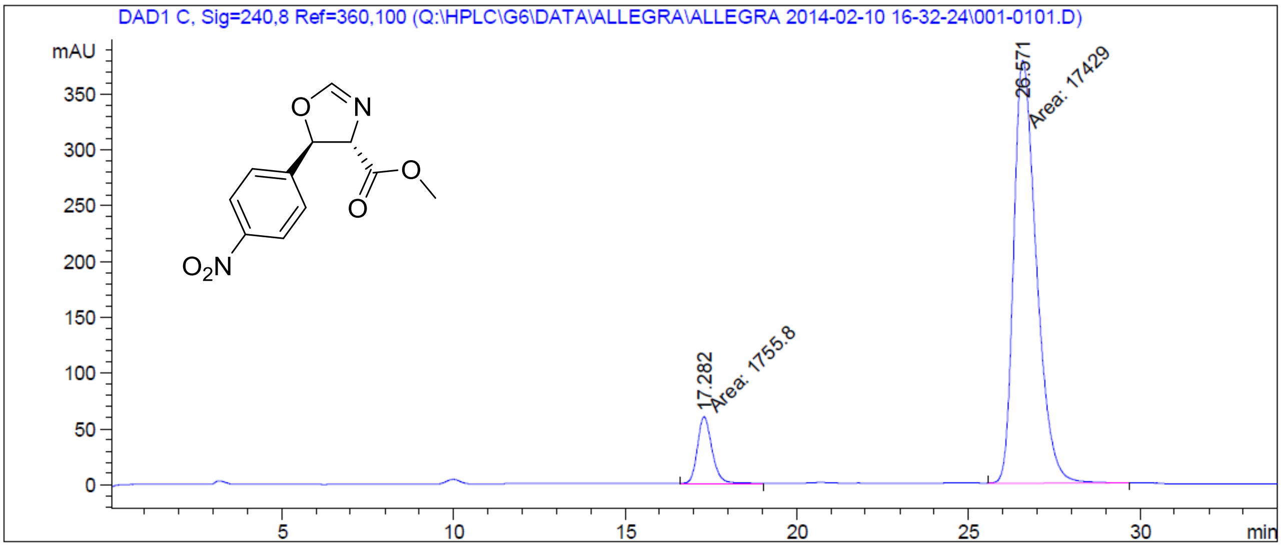

Peak #	RetTime [min]	Type	Width [min]	Area [mAU*s]	Height [mAU]	Area %
1	17.282	MM	0.4898	1755.80188	59.74990	9.1520
2	26.571	MM	0.7665	1.74290e4	378.96262	90.8480

Benzyl (4*S*,5*R*)-5-(4-nitrophenyl)-4,5-dihydro-1,3-oxazole-4-carboxylate (6c)

Chiralpak AD column, hexane/*i*-PrOH 85:15, 1.0 mL/min

Racemic

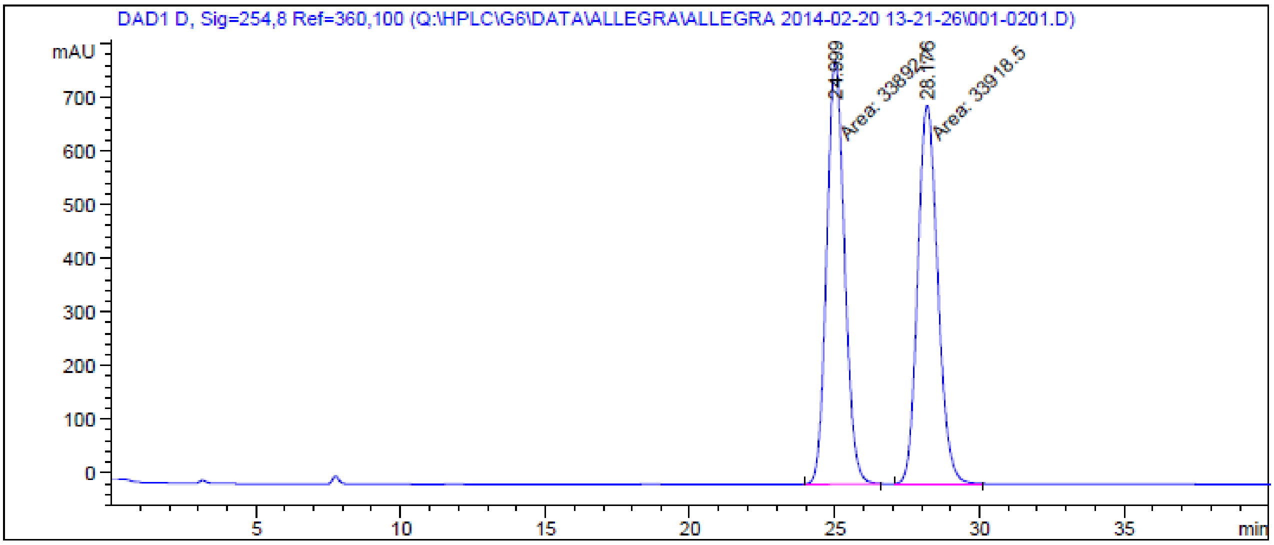

Peak #	RetTime [min]	Type	Width [min]	Area [mAU*s]	Height [mAU]	Area %
1	24.999	MM	0.7164	3.38921e4	788.47339	49.9805
2	28.176	MM	0.8026	3.39185e4	704.35901	50.0195

Enantiomerically enriched (87% ee)

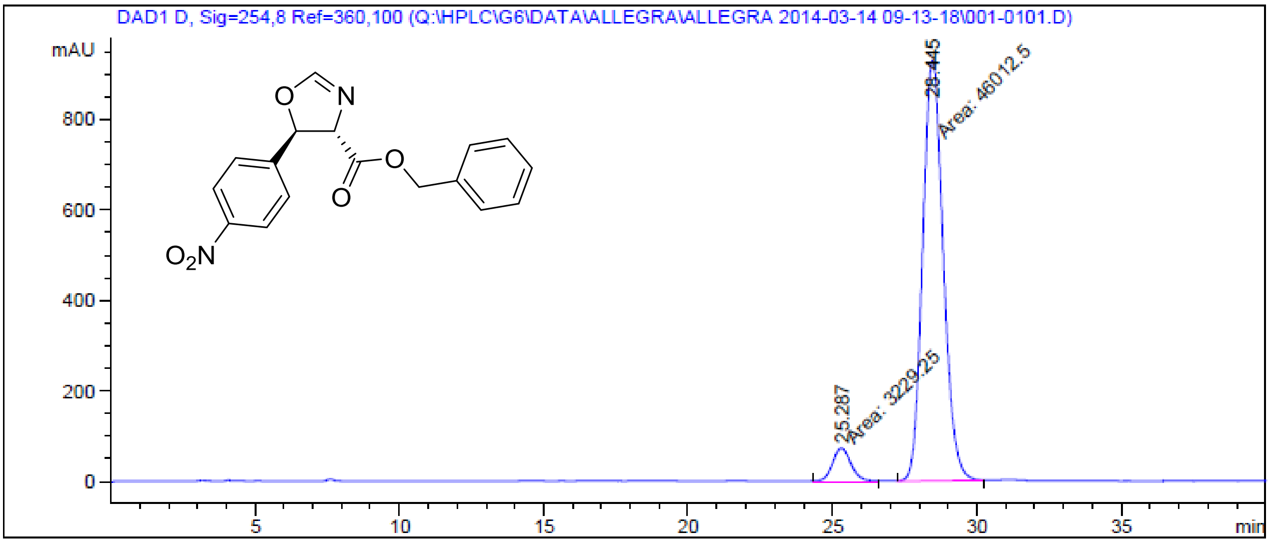

Peak #	RetTime [min]	Type	Width [min]	Area [mAU*s]	Height [mAU]	Area %
1	25.287	MM	0.7370	3229.25269	73.02599	6.5580
2	28.445	MM	0.8240	4.60125e4	930.69653	93.4420

(4-Methoxyphenyl)methyl (4*S*,5*R*)-5-(4-nitrophenyl)-4,5-dihydro-1,3-oxazole-4-carboxylate (6d)

Chiralpak AD column, hexane/*i*-PrOH 80:20, 1.0 mL/min

Racemic

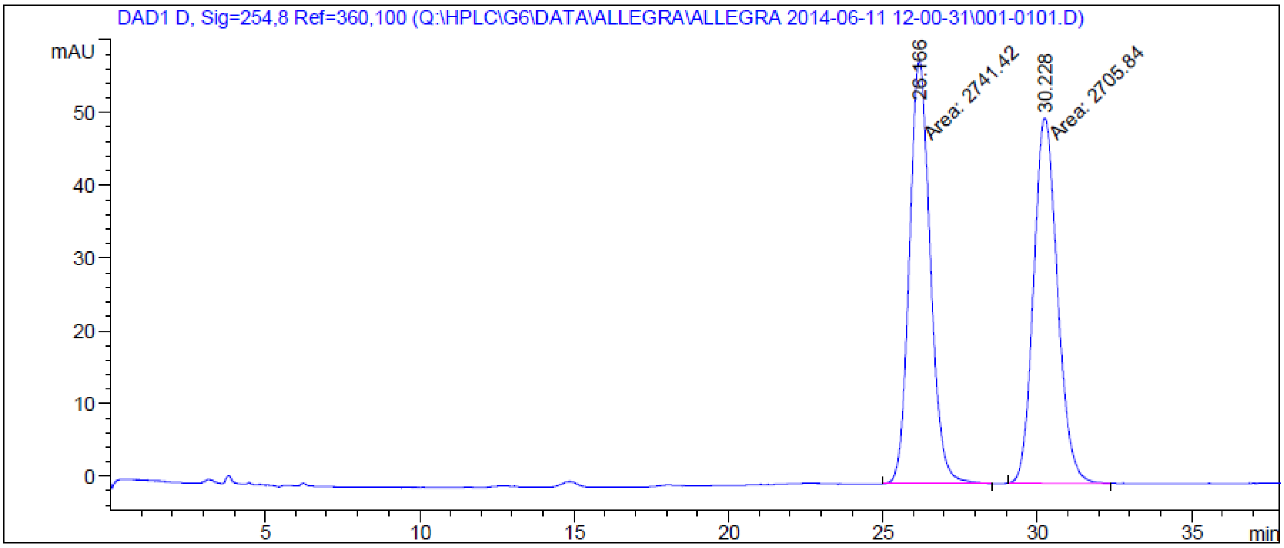

Peak #	RetTime [min]	Type	Width [min]	Area [mAU*s]	Height [mAU]	Area %
1	26.166	MM	0.7856	2741.42114	58.15964	50.3266
2	30.228	MM	0.8968	2705.84351	50.28980	49.6734

Enantiomerically enriched (86% ee)

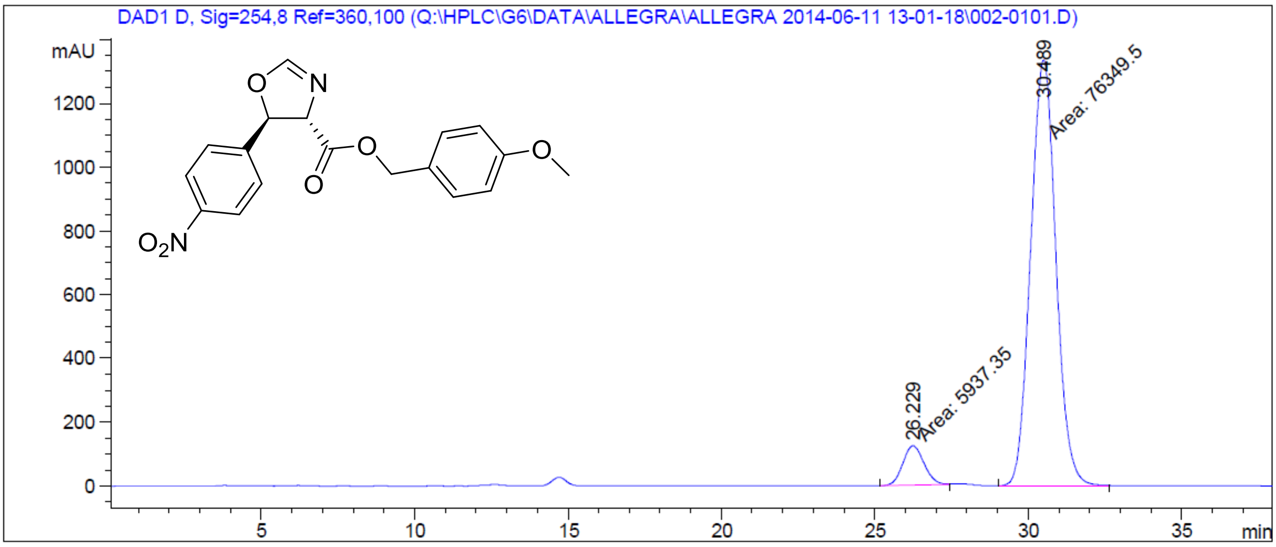

Peak #	RetTime [min]	Type	Width [min]	Area [mAU*s]	Height [mAU]	Area %
1	26.229	MM	0.8026	5937.35059	123.29778	7.2154
2	30.489	MM	0.9533	7.63495e4	1334.82520	92.7846

[3,5-Bis(trifluoromethyl)phenyl]methyl (4*S*,5*R*)-5-(4-nitrophenyl)-4,5-dihydro-1,3-oxazole-4-carboxylate (6e)

Chiralpak AD column, hexane/*i*-PrOH 80:20, 1.0 mL/min

Racemic

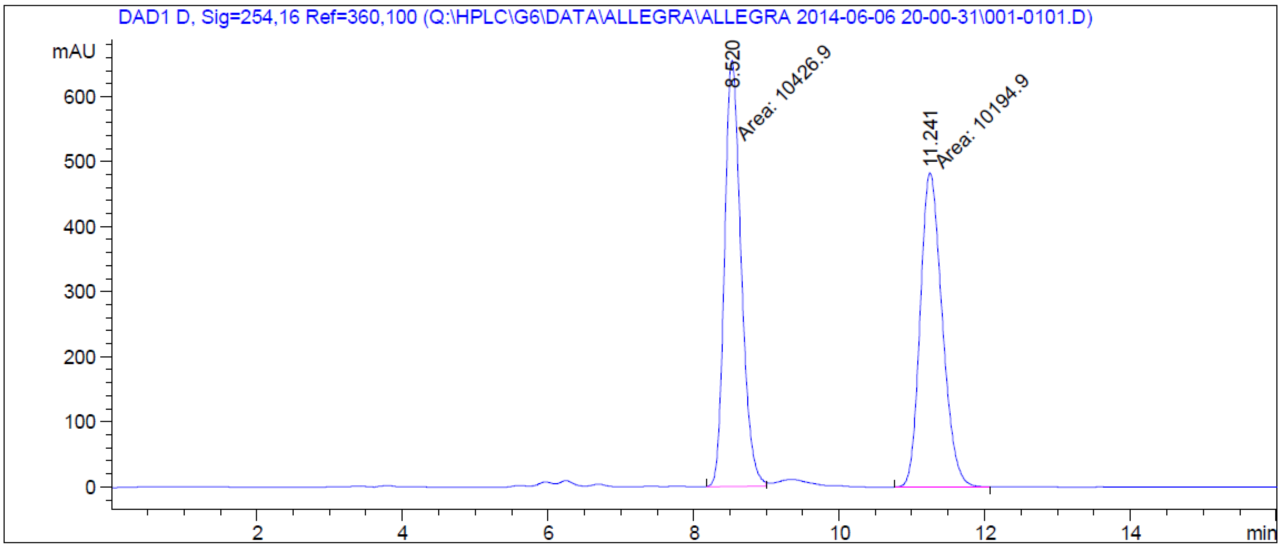

Peak #	RetTime [min]	Type	Width [min]	Area [mAU*s]	Height [mAU]	Area %
1	8.520	MM	0.2651	1.04269e4	655.57812	50.5626
2	11.241	MM	0.3513	1.01949e4	483.64023	49.4374

Enantiomerically enriched (84% ee)

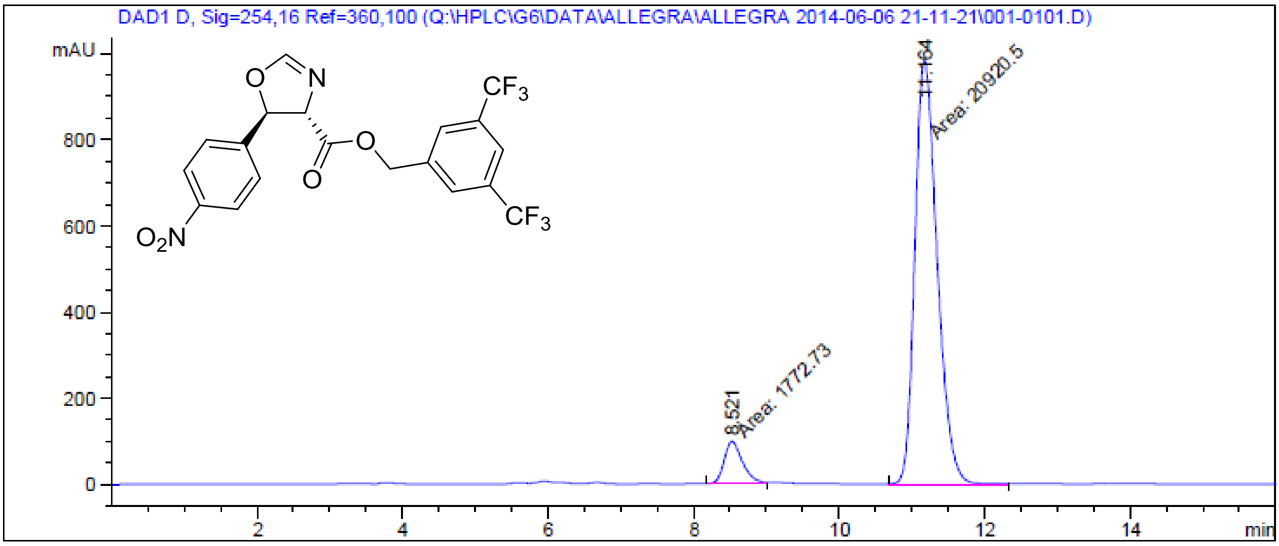

Peak #	RetTime [min]	Type	Width [min]	Area [mAU*s]	Height [mAU]	Area %
1	8.521	MF	0.2991	1772.73328	98.78858	7.8117
2	11.164	MM	0.3540	2.09205e4	984.97302	92.1883

Diphenylmethyl (4*S*,5*R*)-5-(4-nitrophenyl)-4,5-dihydro-1,3-oxazole-4-carboxylate (4*S*,5*R*)-(6f)

Chiralpak OD column, hexane/*i*-PrOH 85:15, 1.0 mL/min

Racemic

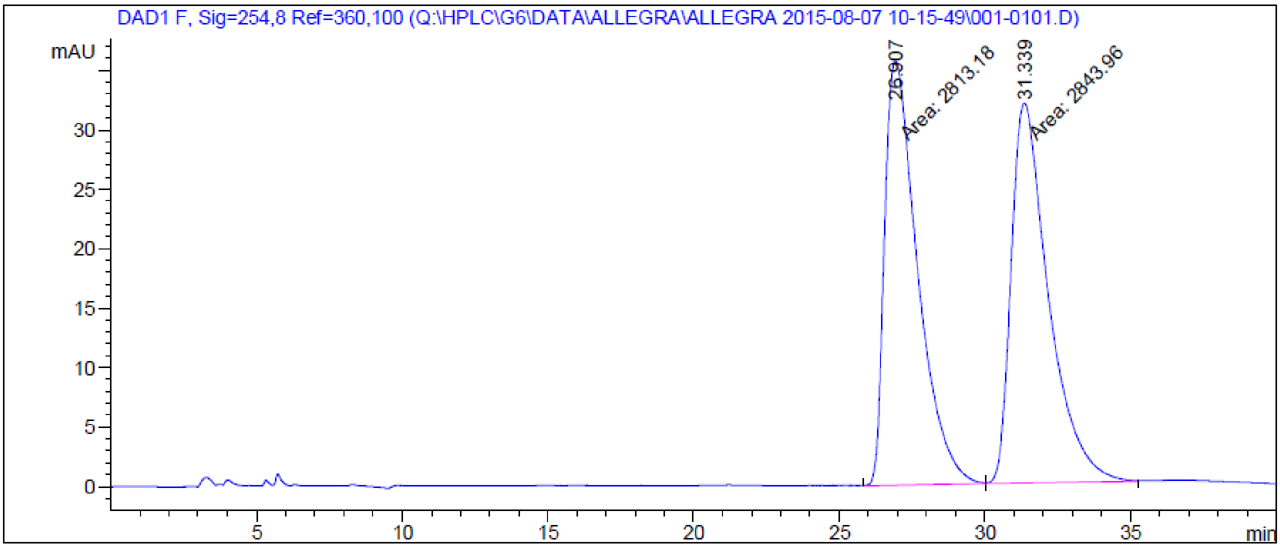

Peak #	RetTime [min]	Type	Width [min]	Area [mAU*s]	Height [mAU]	Area %
1	26.907	MM	1.3131	2813.17822	35.70642	49.7280
2	31.339	MM	1.4836	2843.95801	31.94887	50.2720

Enantiomerically enriched (89% ee)

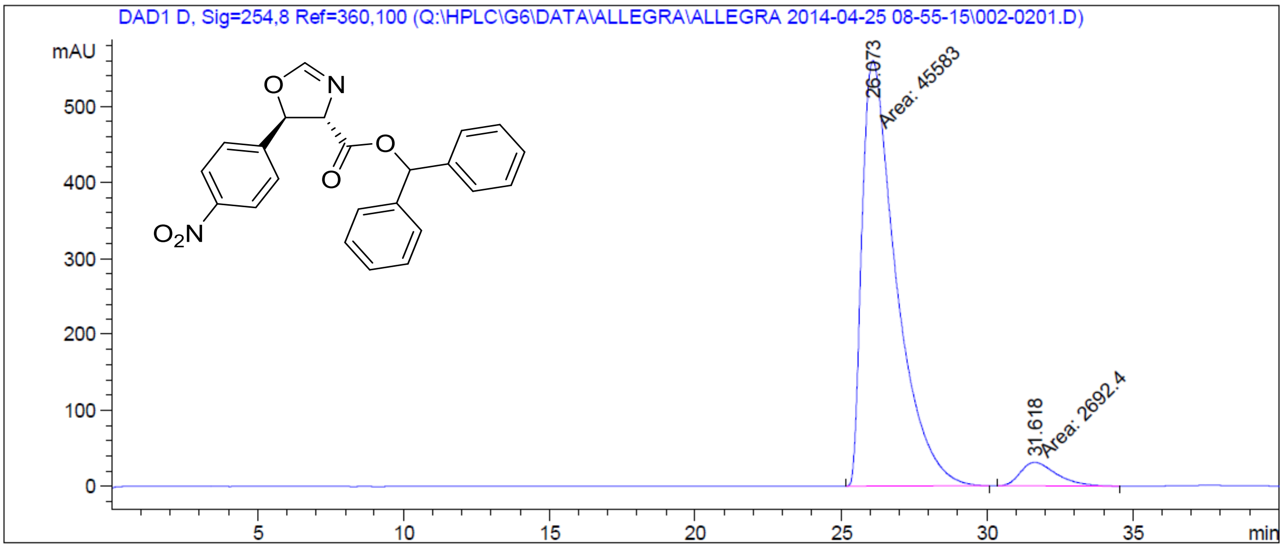

Peak #	RetTime [min]	Type	Width [min]	Area [mAU*s]	Height [mAU]	Area %
1	26.073	MM	1.3563	4.55830e4	560.15430	94.4228
2	31.618	MM	1.4420	2692.39966	31.11789	5.5772

Diphenylmethyl (4*R*,5*S*)-5-(4-nitrophenyl)-4,5-dihydro-1,3-oxazole-4-carboxylate (4*R*,5*S*)-(6f)

Chiralpak OD column, hexane/*i*-PrOH 85:15, 1.0 mL/min

Racemic

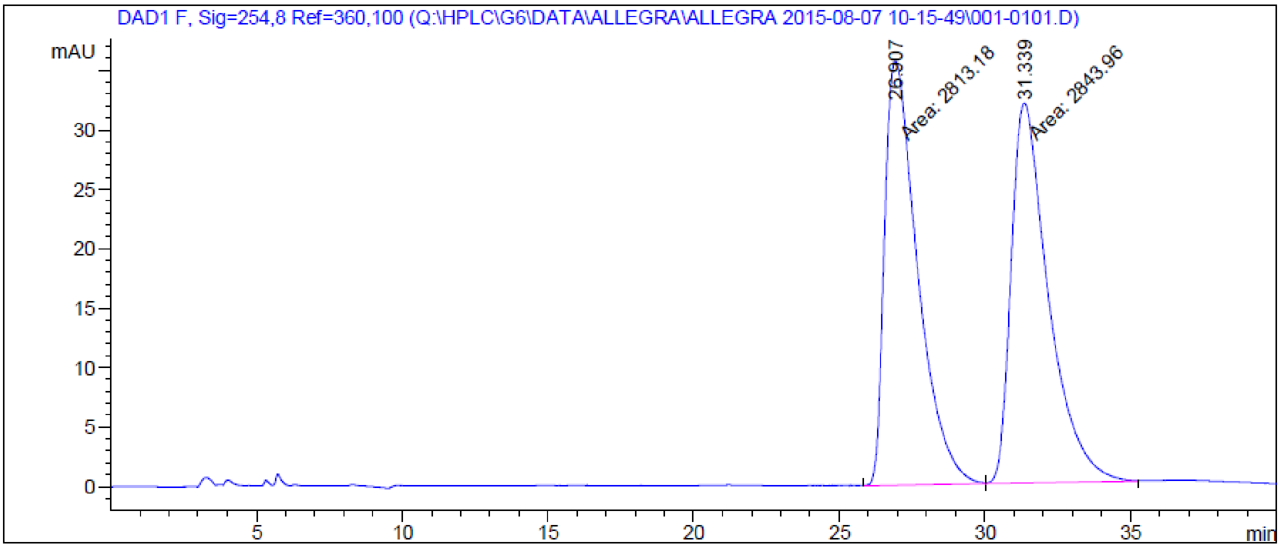

Peak #	RetTime [min]	Type	Width [min]	Area [mAU*s]	Height [mAU]	Area %
1	26.907	MM	1.3131	2813.17822	35.70642	49.7280
2	31.339	MM	1.4836	2843.95801	31.94887	50.2720

Enantiomerically enriched (93% ee)

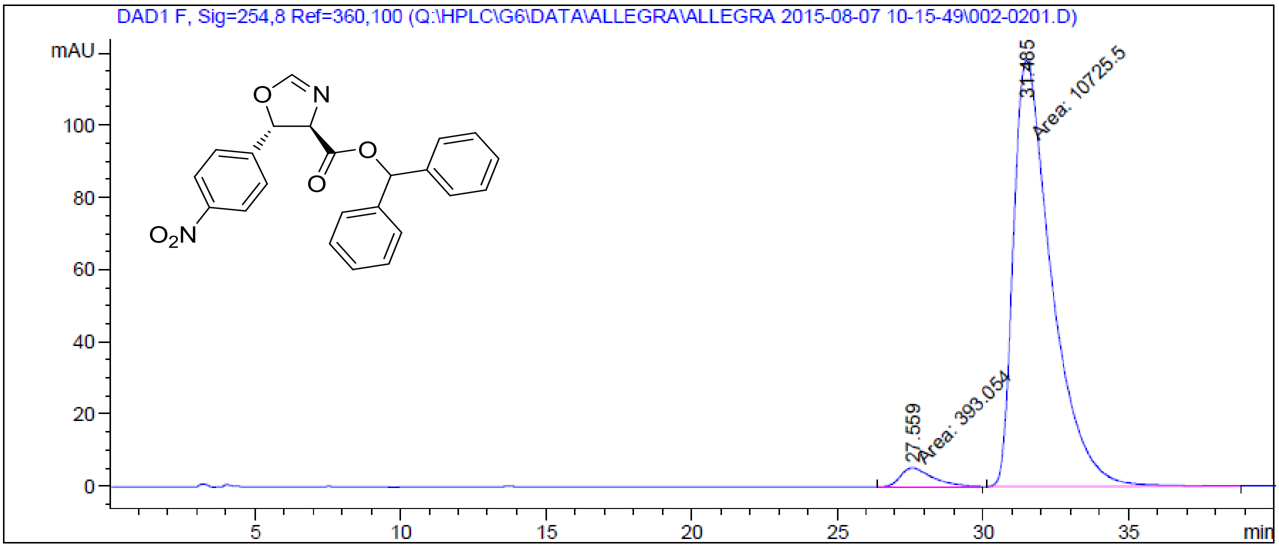

Peak #	RetTime [min]	Type	Width [min]	Area [mAU*s]	Height [mAU]	Area %
1	27.559	MM	1.2665	393.05353	5.17231	3.5351
2	31.485	MM	1.5141	1.07255e4	118.06521	96.4649

Diphenylmethyl (2*S*,3*R*)-2-amino-3-hydroxy-3-(4-nitrophenyl)propanoate (2*S*,3*R*)-(5)

Chiralpak AD column, hexane/*i*-PrOH 70:30, 0.8 mL/min

Racemic

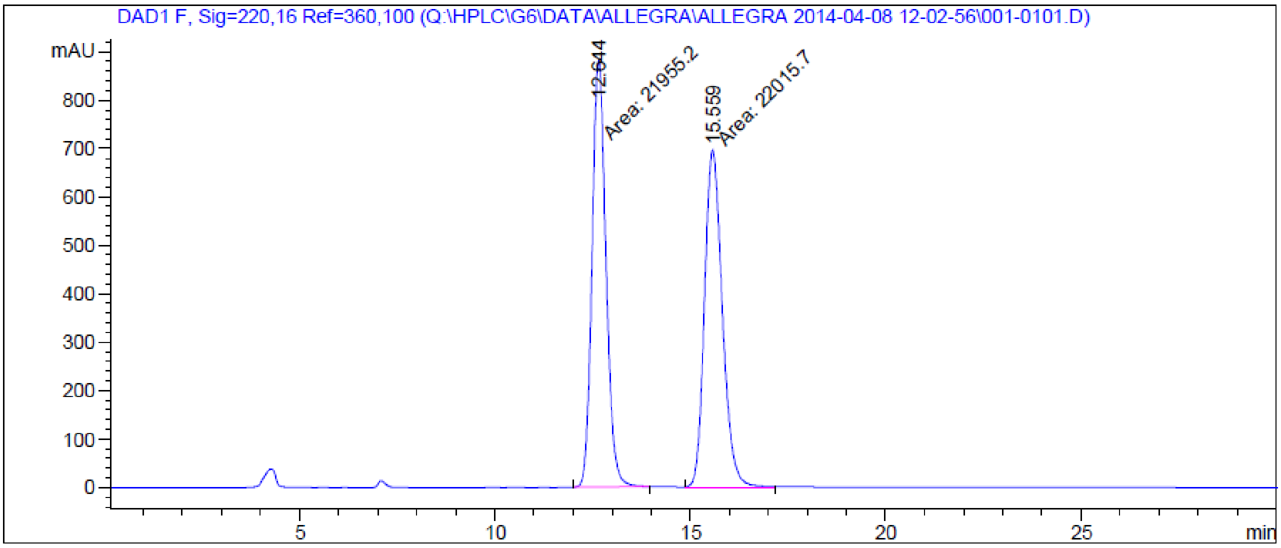

Peak #	RetTime [min]	Type	Width [min]	Area [mAU*s]	Height [mAU]	Area %
1	12.644	MM	0.4150	2.19552e4	881.81708	49.9312
2	15.559	MM	0.5267	2.20157e4	696.66559	50.0688

Enantiomerically enriched (98% ee)

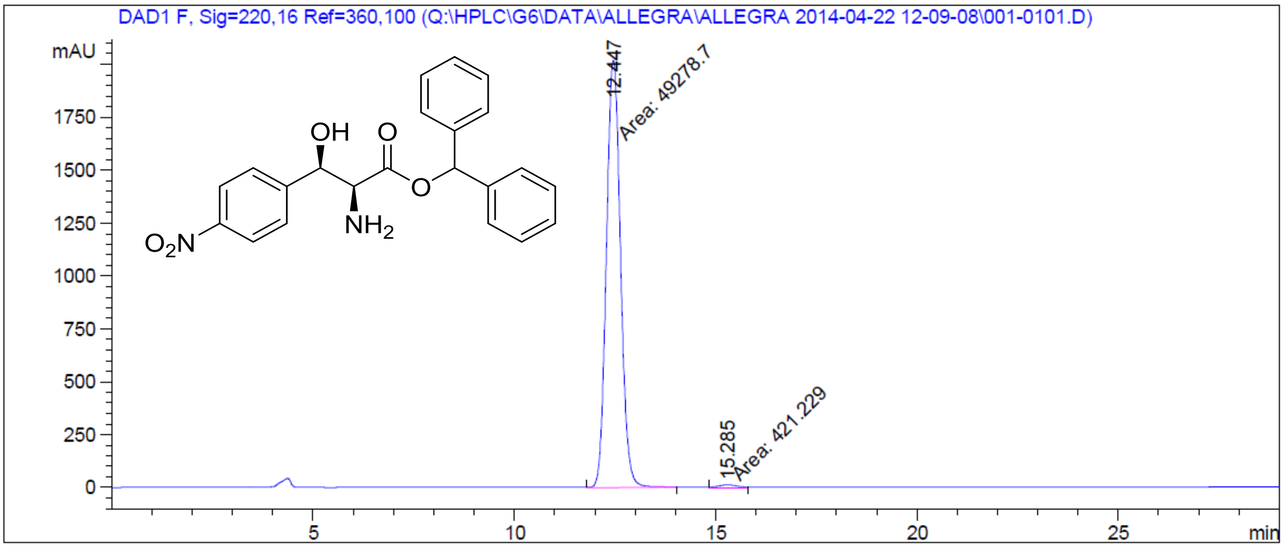

Peak #	RetTime [min]	Type	Width [min]	Area [mAU*s]	Height [mAU]	Area %
1	12.447	MM	0.4066	4.92787e4	2020.15015	99.1525
2	15.285	MM	0.5268	421.22940	13.32771	0.8475

Diphenylmethyl (2*R*,3*S*)-2-amino-3-hydroxy-3-(4-nitrophenyl)propanoate (2*R*,3*S*)-(5)

Chiralpak AD column, hexane/*i*-PrOH 70:30, 0.8 mL/min

Racemic

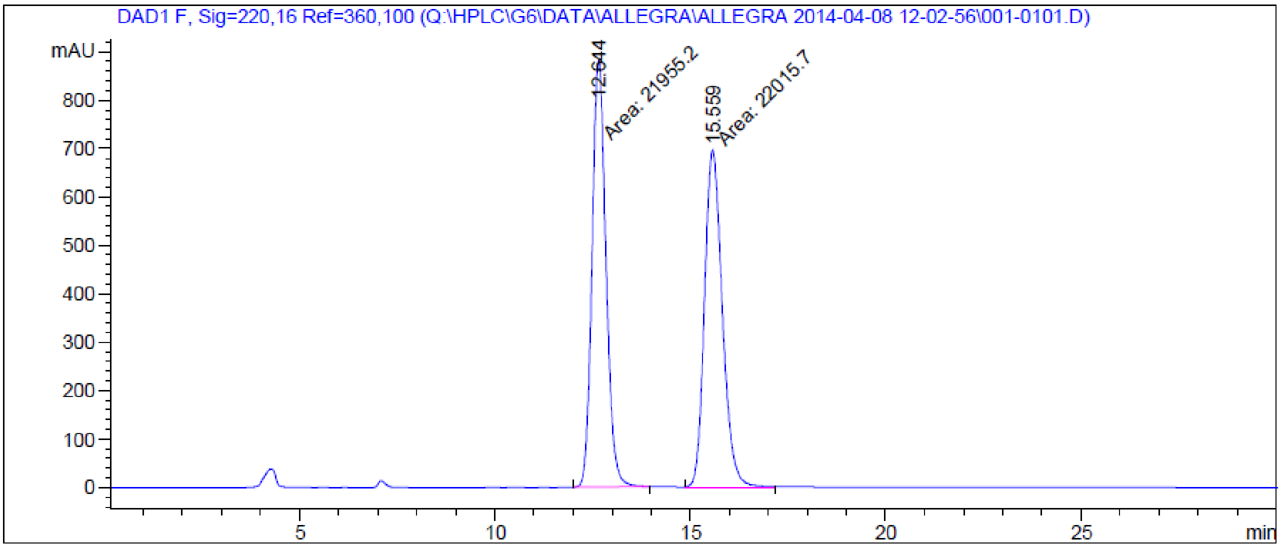

Peak #	RetTime [min]	Type	Width [min]	Area [mAU*s]	Height [mAU]	Area %
1	12.644	MM	0.4150	2.19552e4	881.81708	49.9312
2	15.559	MM	0.5267	2.20157e4	696.66559	50.0688

Enantiomerically enriched (94% ee)

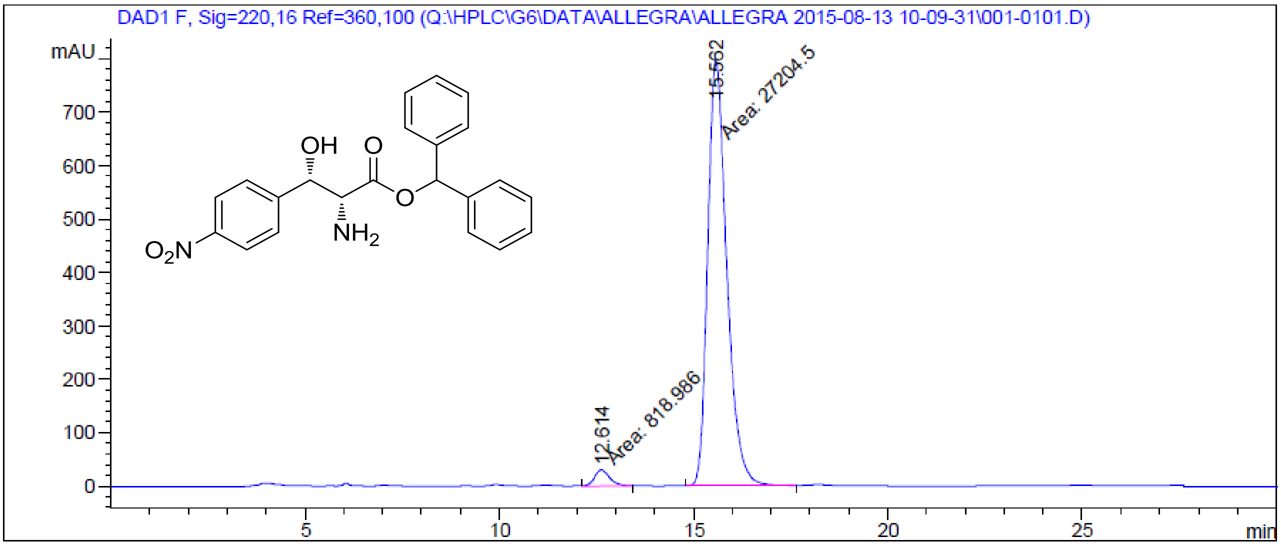

Peak #	RetTime [min]	Type	Width [min]	Area [mAU*s]	Height [mAU]	Area %
1	12.614	MM	0.4463	818.98645	30.58410	2.9225
2	15.562	MM	0.5696	2.72045e4	795.97314	97.0775

Diphenylmethyl (2*S*,3*R*)-2-(2,2-dichloroacetamido)-3-hydroxy-3-(4-nitrophenyl)propanoate (2*S*,3*R*)-(9)

Chiralpak AD column, hexane/*i*-PrOH 70:30, 0.8 mL/min

Racemic

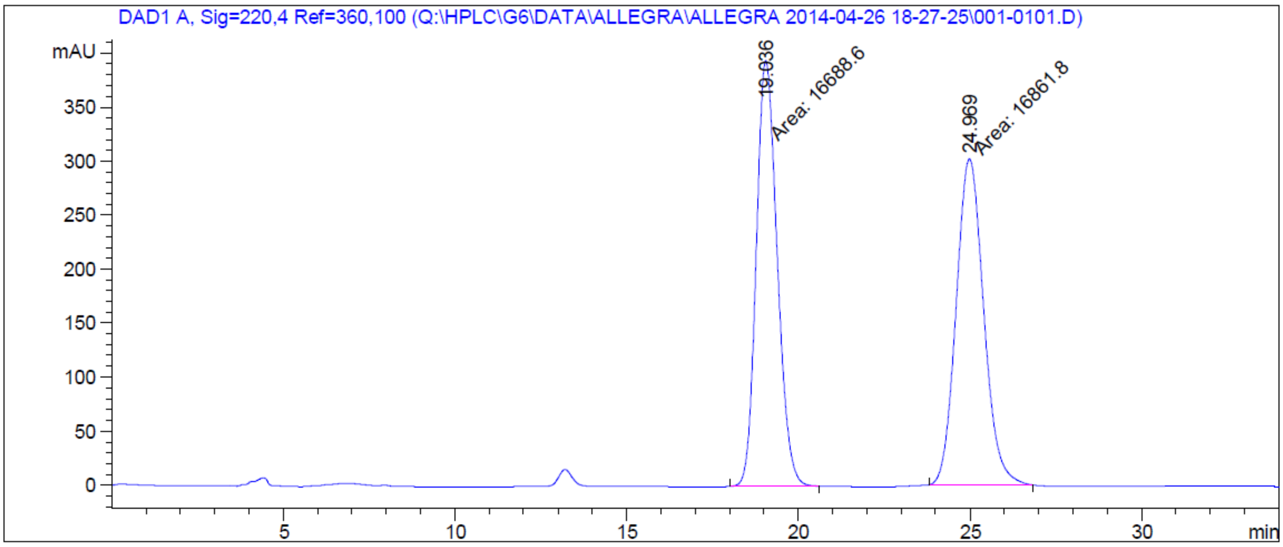

Peak #	RetTime [min]	Type	Width [min]	Area [mAU*s]	Height [mAU]	Area %
1	19.036	MM	0.7055	1.66886e4	394.22455	49.7419
2	24.969	MM	0.9305	1.68618e4	302.02881	50.2581

Enantiomerically enriched (98% ee)

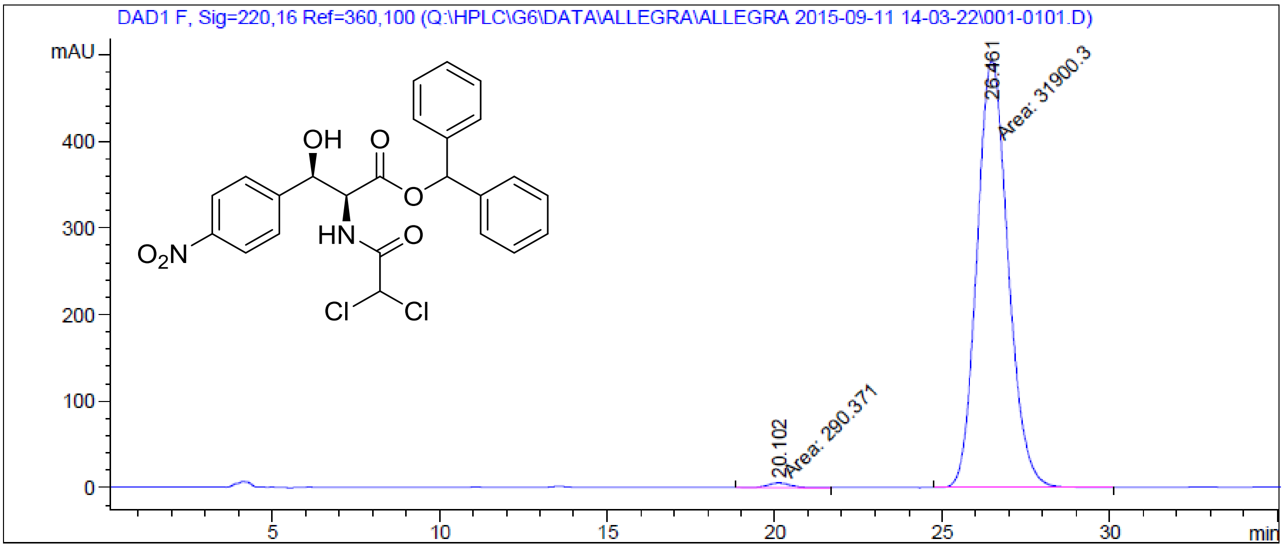

Peak #	RetTime [min]	Type	Width [min]	Area [mAU*s]	Height [mAU]	Area %
1	20.102	MM	0.8492	290.37076	5.69907	0.9020
2	26.461	MM	1.0785	3.19003e4	492.96762	99.0980

Diphenylmethyl (2*R*,3*S*)-2-(2,2-dichloroacetamido)-3-hydroxy-3-(4-nitrophenyl)propanoate (2*R*,3*S*)-(9)

Chiralpak AD column, hexane/*i*-PrOH 70:30, 0.8 mL/min

Racemic

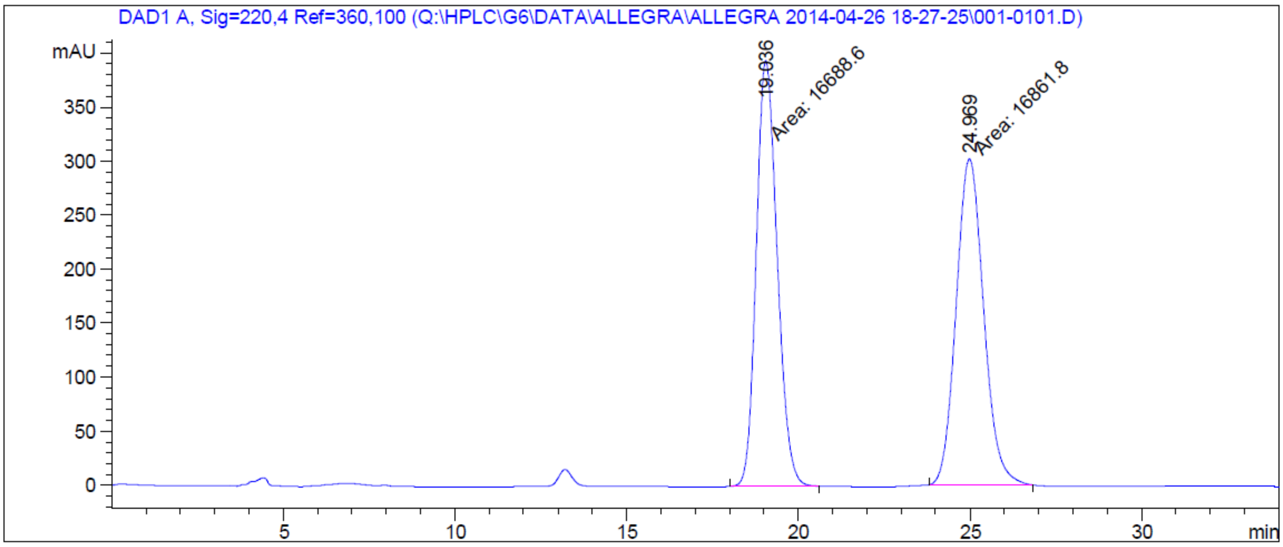

Peak #	RetTime [min]	Type	Width [min]	Area [mAU*s]	Height [mAU]	Area %
1	19.036	MM	0.7055	1.66886e4	394.22455	49.7419
2	24.969	MM	0.9305	1.68618e4	302.02881	50.2581

Enantiomerically enriched (93% ee)

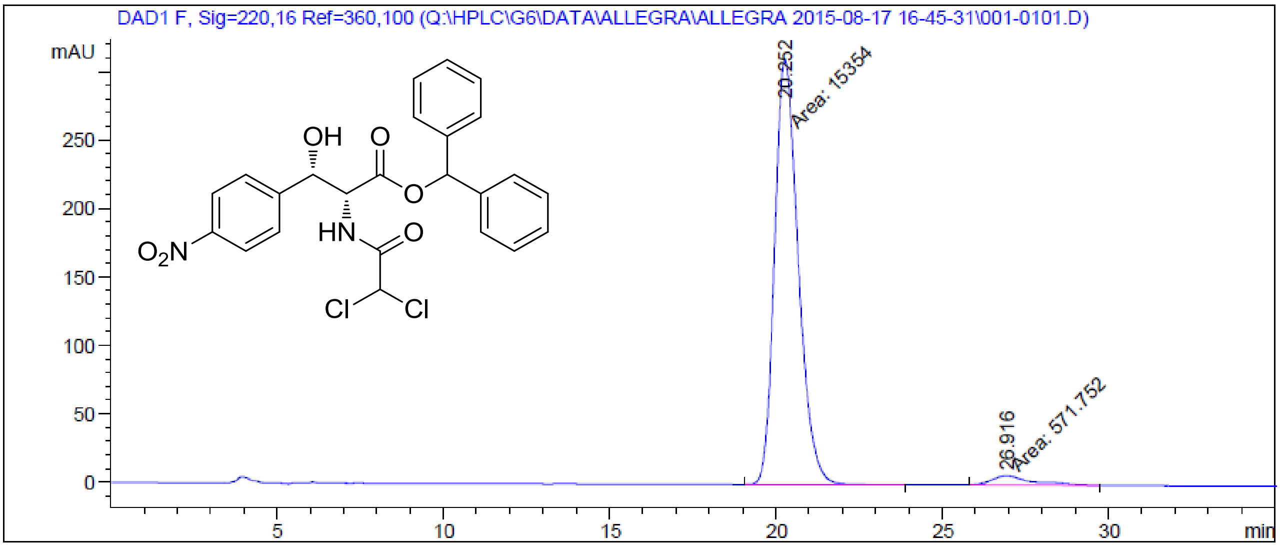

Peak #	RetTime [min]	Type	Width [min]	Area [mAU*s]	Height [mAU]	Area %
1	20.252	MM	0.8255	1.53540e4	309.98349	96.4099
2	26.916	MM	1.3998	571.75244	6.80745	3.5901

(−)-Chloramphenicol (−)-(1)
2,2-dichloro-*N*-[(1*R*,2*R*)-1,3-dihydroxy-1-(4-nitrophenyl)propan-2-yl]acetamide

Chiralpak AD-H column, hexane/*i*-PrOH 90:10, 1.0 mL/min

Racemic

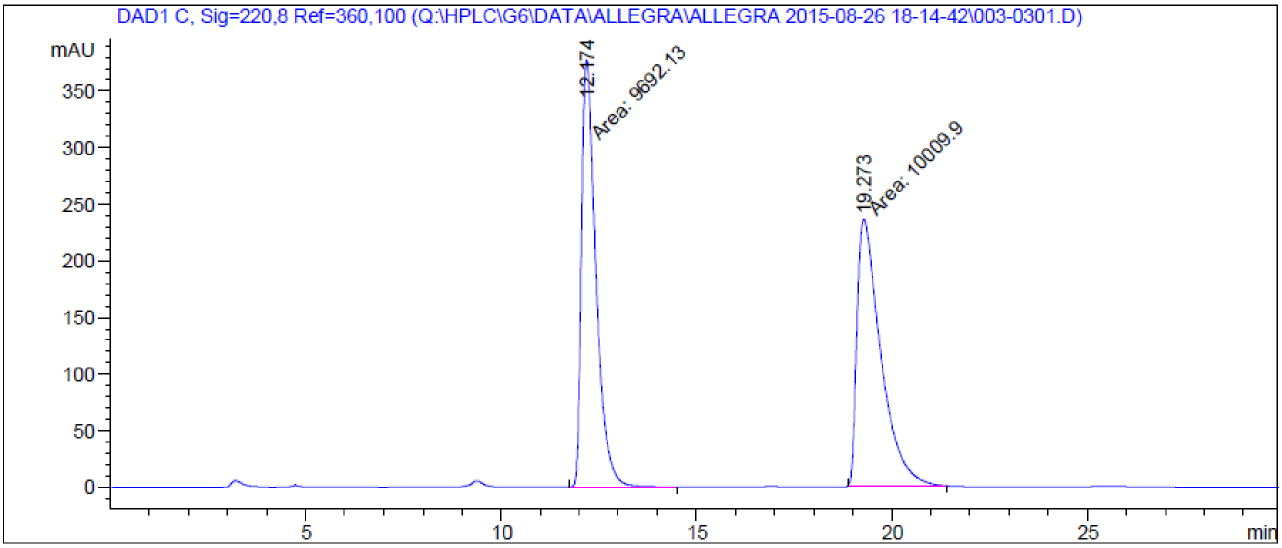

Peak #	RetTime [min]	Type	Width [min]	Area [mAU*s]	Height [mAU]	Area %
1	12.174	MM	0.4283	9692.13184	377.17380	49.1936
2	19.273	MM	0.7076	1.00099e4	235.76247	50.8064

Enantiomerically enriched (99% ee)

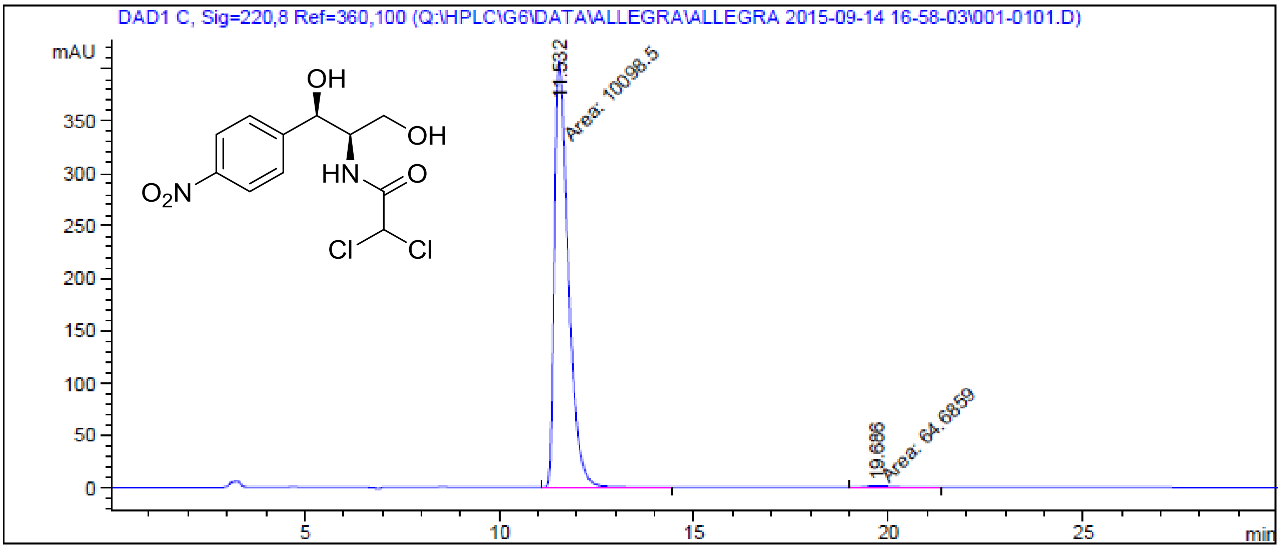

Peak #	RetTime [min]	Type	Width [min]	Area [mAU*s]	Height [mAU]	Area %
1	11.532	MM	0.4134	1.00985e4	407.15997	99.3635
2	19.686	MM	0.7680	64.68589	1.40372	0.6365

(+)-Chloramphenicol (+)-(1)
2,2-dichloro-*N*-[(1*S*,2*S*)-1,3-dihydroxy-1-(4-nitrophenyl)propan-2-yl]acetamide

Chiralpak AD-H column, hexane/*i*-PrOH 90:10, 1.0 mL/min

Racemic

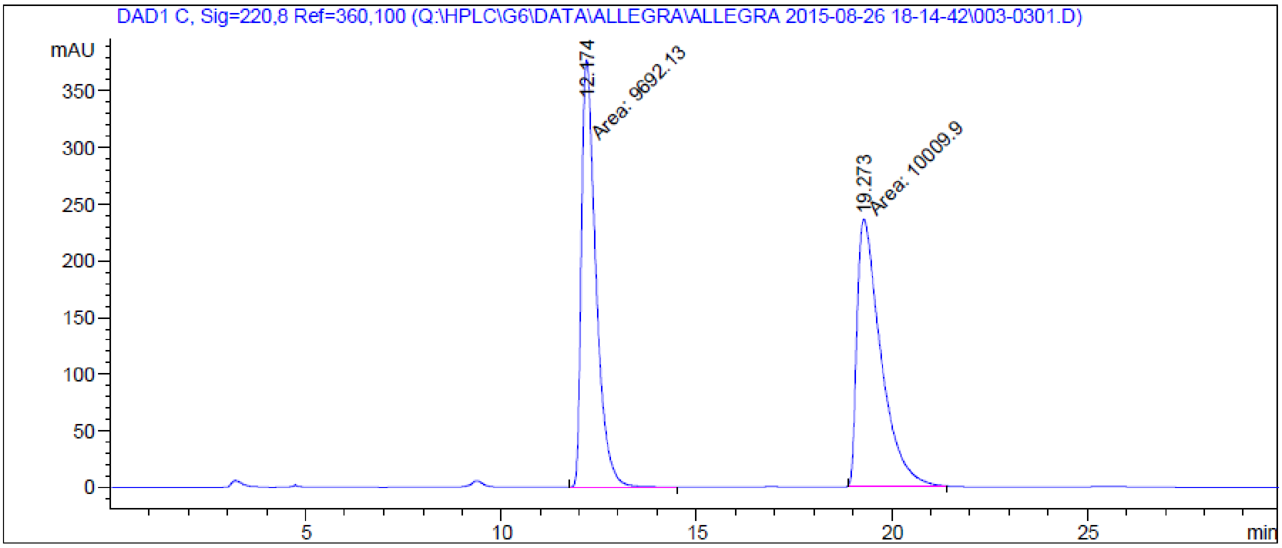

Peak #	RetTime [min]	Type	Width [min]	Area [mAU*s]	Height [mAU]	Area %
1	12.174	MM	0.4283	9692.13184	377.17380	49.1936
2	19.273	MM	0.7076	1.00099e4	235.76247	50.8064

Enantiomerically enriched (96% ee)

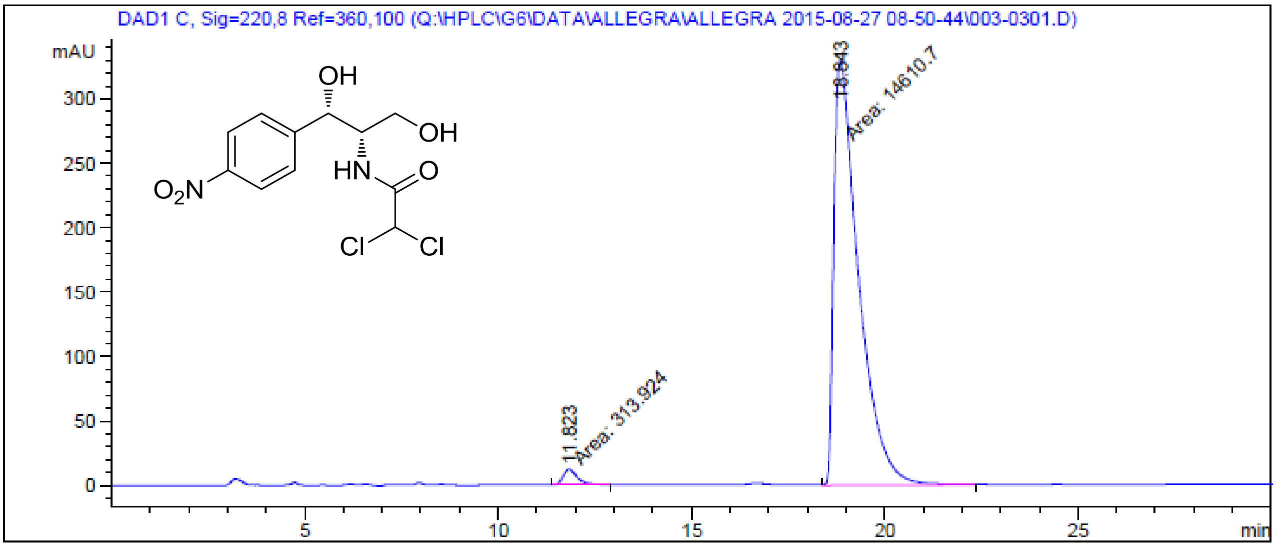

Peak #	RetTime [min]	Type	Width [min]	Area [mAU*s]	Height [mAU]	Area %
1	11.823	MM	0.4304	313.92361	12.15549	2.1034
2	18.843	MM	0.7399	1.46107e4	329.10623	97.8966

5. References

[1] Still, W. C.; Kahn, M.; Mitra, A. *J. Org. Chem.* **1978,** *43*, 2923.
[2] Fulmer, G. R.; Miller, A. J. M.; Sherden, N. H.; Gottlieb, H. E.; Nudelman, A.; Stoltz, B. M.; Bercaw, J. E.; Goldberg, K. I. *Organometallics* **2010**, *29*, 2176.
[3] Aizpurua, J. M.; Palomo, C. *Synth. Commun.* **1983,** *13*, 745.
[4] Sladojevich, F.; Trabocchi, A.; Guarna, A.; Dixon, D. J. *J. Am. Chem. Soc.* **2011,** *133*, 1710.
[5] De la Campa, R.; Ortín, I.; Dixon, D. J. *Angew. Chem. Int. Ed.* **2015,** *54*, 4895.
[6] Novak, B. H.; Lash, T. D. *J. Org Chem.* **1998,** *63*, 3998.
[7] Allen, C. L.; Atkinson, B. N.; Williams, J. M. J. *Angew. Chem. Int. Ed.* **2012,** *51*, 1383.
[8] Ortín, I.; Dixon, D. J. *Angew. Chem. Int. Ed.* **2014,** *53*, 3462.
[9] Burns, D. H.; Jabara, C. S.; Burden, M. W. *Synth. Commun.* **1995,** *25*, 379.
[10] Hoppe, D.; Schmincke, H.; Kleemann, H.-W. *Tetrahedron Lett.* **1989**, *45*, 687. Elemental analysis, ^{1}H NMR, IR and melting point are reported.
[11] Hayashi, T.; Sawamura, M.; Ito, Y. *Tetrahedron* **1992,** *48,* 1999.
[12] Chênevert, R.; Thiboutot, S. *Synthesis* **1989,** 444.
[13] George, S.; Narina, S. V.; Sudalai, A. *Tetrahedron* **2006,** *62*, 10202.
[14] Bartz, Q. R. *J. Biol. Chem.* **1948,** *172*, 445.
[15] Rebstock, M. C.; Crooks, H. M.; Controulis, J.; Bartz, Q. R. *J. Am. Chem. Soc.* **1949,** *71*, 2458.
[16] Controulis, J.; Rebstock, M. C.; Crooks, H. M *J. Am. Chem. Soc*. **1949,** *71*, 2463.
